# Computational Study of Asian Propolis Compounds as Potential Anti-Type 2 Diabetes Mellitus Agents by Using Inverse Virtual Screening with the DIA-DB Web Server, Tanimoto Similarity Analysis, and Molecular Dynamic Simulation

**DOI:** 10.3390/molecules27133972

**Published:** 2022-06-21

**Authors:** Putri Hawa Syaifie, Azza Hanif Harisna, Mochammad Arfin Fardiansyah Nasution, Adzani Gaisani Arda, Dwi Wahyu Nugroho, Muhammad Miftah Jauhar, Etik Mardliyati, Nurwenda Novan Maulana, Nurul Taufiqu Rochman, Alfian Noviyanto, Antonio J. Banegas-Luna, Horacio Pérez-Sánchez

**Affiliations:** 1 Nano Center Indonesia, South Tangerang 15314, Indonesia; putri@nano.or.id (P.H.S.); harisna@nano.or.id (A.H.H.); gaisani.arda@nano.or.id (A.G.A.); wahyu@nano.or.id (D.W.N.); mmiftahjauhar@nano.or.id (M.M.J.); novan@nano.or.id (N.N.M.); a.noviyanto@nano.or.id (A.N.); 2 Department of Chemistry, Faculty of Mathematics and Natural Sciences, Universitas Indonesia, Depok 16424, Indonesia; marfin.f@sci.ui.ac.id; 3 Research Center for Vaccine and Drug, National Research and Innovation Agency (BRIN), Bogor 16911, Indonesia; 4 Research Center for Advanced Material, National Research and Innovation Agency (BRIN), South Tangerang 15314, Indonesia; nurul@nano.or.id; 5 Department of Mechanical Engineering, Faculty of Engineering, Mercu Buana University, Jakarta 11650, Indonesia; 6 Structural Bioinformatics and High Performance Computing Research Group (BIO-HPC), Universidad Católica de Murcia, 30107 Murcia, Spain; ajbanegas@ucam.edu

**Keywords:** type-2 diabetes mellitus, propolis, DIA-DB, in silico, virtual screening, molecular dynamic

## Abstract

Propolis contains a wide range of pharmacological activities because of their various bioactive compounds. The beneficial effect of propolis is interesting for treating type-2 diabetes mellitus (T2DM) owing to dysregulation of multiple metabolic processes. In this study, 275 of 658 Asian propolis compounds were evaluated as potential anti-T2DM agents using the DIA-DB web server towards 18 known anti-diabetes protein targets. More than 20% of all compounds could bind to more than five diabetes targets with high binding affinity (<−9.0 kcal/mol). Filtering with physicochemical and pharmacokinetic properties, including ADMET parameters, 12 compounds were identified as potential anti-T2DM with favorable ADMET properties. Six of those compounds, (2*R*)-7,4′-dihydroxy-5-methoxy-8-methylflavone; (*RR*)-(+)-3′-senecioylkhellactone; 2′,4′,6′-trihydroxy chalcone; alpinetin; pinobanksin-3-*O*-butyrate; and pinocembrin-5-methyl ether were first reported as anti-T2DM agents. We identified the significant T2DM targets of Asian propolis, namely retinol-binding protein-4 (RBP4) and aldose reductase (AKR1B1) that have important roles in insulin sensitivity and diabetes complication, respectively. Molecular dynamic simulations showed stable interaction of selected propolis compounds in the active site of RBP4 and AKR1B1. These findings suggest that Asian propolis compound may be effective for treatment of T2DM by targeting RBP4 and AKR1B1.

## 1. Introduction

Type 2 diabetes mellitus (T2DM) is a common type of diabetes disease caused by insufficient insulin action to insulin-sensitive tissues (insulin resistance), which leads to dysfunction of beta-pancreatic cells to produce insulin and high blood glucose levels. More than 90% of diabetes cases were dominated by T2DM [1]. Worldwide cases of T2DM are predicted to reach 592 million in 2035, and Asia is the main area affected by this disease, especially in China and India [2,3]. This is likely caused by rapid changes in lifestyle behaviors in Asia. There is increasing carbohydrate and saturated fat intake and decreasing intake of quality protein, healthy fruits, and vegetables, which may lead to dysregulation of lipid, protein, and carbohydrate metabolism and an increased risk of T2DM [1,3].

There are some primary class of oral antidiabetic mediations as sulfonylureas, biguanides, DPP4 inhibitors, thiazolidinediones, sodium-glucoshydroxysteroid (SGLT2) inhibitors and others. A previous publication reported that the major side effect of sulfonylureas drugs is hypoglycemic and it was contraindicated for hepatic patients and pregnant women. Metformin as biguanides can cause folic acid and vitamin B1 deficiency. In clinical trials, the adverse effects of DPP4 inhibitors (sitagliptin, saxagliptin, vildagliptin, linagliptin, and alogliptin) are nasopharyngitis, upper respiratory tract infection, and headaches. Combined treatment with insulin and thiazolidinedione drugs may cause heart failure [4]. These undesirable side effects of synthetic medications have limited their application.

Considerable evidence indicates that the bioactive compounds in herbal medicine have less toxicity and adverse effects. Natural compounds are safer for daily consumption in the treatment of diseases than synthetic drugs. Herbal medicine has a wide range of biological effects and has a superior holistic quality of treatment. They have excellent antidiabetic properties in controlling glucose homeostasis, blood lipid regulation, anti-inflammatory, and antioxidant activities. Herbal medicine can be an excellent complementary and alternative treatment for T2DM through multi-targets pharmacological of action. The ability to regulate multiple targets might be more effective in treating T2DM because the disease is caused by dysregulation of numerous processes [5,6]. Propolis supplementation is an effective and promising natural product for diabetes, because it contains many bioactive compounds [7,8]. More than 300 bioactive compounds have been identified in propolis with a wide range of pharmacological effects [9,10].

Propolis collected by worker bees (*Apis mellifera*, *Trigona* sp., *Tetragonula* sp. and others). Their active compounds originated from various plants that produce resinous secretions to repair the beehive and protect the colony against infections. Propolis contains a mixture of polyphenols, flavonoids, terpenoids, aromatic acids, steroids, essential oils, aromatic oils, and other bioactive compounds. The bioactive compounds of propolis vary based on geographical origin, plant sources, bee genus and species, and subtropical/tropical areas. These propolis’ bioactive compounds have a wide range of pharmacological properties, including antidiabetic activities [9,10]. Recent studies showed that propolis has antidiabetic activity, as evaluated through in vitro, in vivo, and clinical studies [11,12,13,14,15]. Propolis compounds, such as chrysin and caffeic acid phenyl ester, exhibit excellent potential antidiabetic activity by reducing blood glucose levels on proliferator diabetic rats [13]. Six randomized controlled trials showed that propolis consumption significantly reduced fasting plasma glucose in diabetic patients [16]. Another clinical study showed that a daily consumption of 900 mg propolis supplement improves glycemic and serum lipid levels in T2DM patients within 12 weeks [11]. Chinese propolis have been used as supplementations for T2DM patient in 6 months consumption with the condition of reducing hemoglobin (Hb) A1c, fasting plasma glucose (FPG), and improving periodontal parameters [17]. However, the mechanism of superior antidiabetic activity of propolis is still rare and there is limited information on the compounds that play an essential role in antidiabetic.

T2DM is a complex chronic disorder of glucose homeostasis and regulation of lipids and insulin secretion and sensitivity. Multiple antidiabetic agents from rich contains of natural compounds are required. The exploration of propolis as a potential natural medicine for targeting multiple diabetes targets has been rarely reported. Therefore, screening propolis compounds with antidiabetic activity is needed to explore the potential antidiabetic compounds and predict the mechanism. This study used an in silico investigation, a molecular docking approach, using a developed method (DIA-DB webserver) to screen compounds with potential antidiabetic activity against 18 T2DM targets. The DIA-DB web server (http://bio-hpc.eu/software/dia-db/, accessed on 2 September 2020). The DIA-DB web server (http://bio-hpc.eu/software/dia-db/, accessed on 2 September 2020) uses two different approaches: inverse virtual screening of the input compounds against 18 T2DM targets and comparison by shape similarity against a curated database of approved antidiabetic drugs [18,19]. Previous reports have identified 28 anti-T2DM compounds from 867 compounds in African medicinal plants by DIA-DB web server [20]. Two classes of bioactive compounds in herbs and spices: sesquiterpenoids and flavonoids have been reported as a significant ingredient for anti-diabetic based on in silico inverse virtual screening method by using DIA-DB web server evaluation [20]. The aim of this study was to evaluate Asian propolis compounds and their anti-T2DM activity with multiple diabetes targets. The similarity of the Asian propolis compounds to FDA-approved drugs was also thoroughly examined. The predicted physicochemical, pharmacokinetic, and toxicity properties of the Asian propolis compounds were analyzed using the ADMETlab web server. Molecular dynamic simulation of selected compounds also used to define the stability of compounds in binding site of diabetic protein targets.

## 2. Results

### 2.1. Inverse Virtual Screening and Identification of Propolis Compounds with Potential Anti-T2DM Activity

A total of 658 Asian propolis compounds was collected and listed in Table S1 (Supplementary Materials) [21,22,23,24,25,26,27,28,29,30,31,32,33,34,35,36,37,38,39,40,41,42,43,44,45,46,47,48,49,50,51,52,53,54,55,56,57,58,59,60,61,62,63,64,65,66,67,68,69,70,71,72,73,74,75,76,77,78,79,80,81,82,83,84,85,86,87,88,89,90,91,92,93,94,95,96,97,98,99,100,101,102,103,104,105,106,107,108,109,110,111,112,113,114,115,116,117]. These compounds were screened by inverse virtual docking against 18 protein tin argents of T2DM using the DIA-DB web server [20]. These target proteins are involved in the three types of T2DM mechanisms of action, that is, regulation of insulin secretion and sensitivity, regulation of glucose metabolism, and regulation of lipid metabolism, as summarized in Table 1.

**Table 1:** Summary of inverse virtual screening of Asian propolis compounds.

Mode of Action	Protein Target	Function	PDB Code	Percentage of Potential Compounds (Total of Potential Compounds/Total of Test Compounds)	Test Compounds with the Lowest Energy (Kcal/mol)	Test Compound Name with the Lowest Binding Energy
Regulation of insulin secretion and sensitivity	DPP4	Cleaves and inactivates glucagon-like peptide-1 that stimulates insulin secretion and inhibits glucagon secretion [118,119]	4A5S	10.3% (68/658)	−10.90	Retusapurpurin A
	FFAR1	G-protein-coupled receptors bind with a free fatty acid or its specific agonist to support beta-cell function for insulin secretion [120,121]	4PHU	11.1% (73/658)	−10.40	TB1 and isonymphaeol-B
	HSD11B1	Catalyzes the interconversion of inactive glucocorticoid (cortisone) to active glucocorticoid (cortisol) that stimulates insulin resistance [122,123]	4K1L	20.8% (137/658)	−11.90	23-hydroxymangiferonic acid
	INSR	Expressed in insulin-responsive cells and acts as the initial point of insulin signaling [124]	3EKN	0.9% (6/658)	−10.10	Taraxasterol
	PTPN9	A negative regulator of insulin signaling via catalyzing the rapid dephosphorylation of insulin receptor resulting in insulin resistance [124,125]	4GE6	0.3% (2/658)	−9.40	Garcinone B
	RBP4	An adipocyte-secreted molecule that activates innate immune responses and induces inflammation resulting in insulin resistance [126]	2WR6	12.5% (82/658)	−12.40	10-hydroxybenzo[j]fluoranthene
Regulation of glucose metabolism	AKR1B1	Catalyzes the reduction of glucose to sorbitol in the polyol pathway, thus contributing to diabetes complications [127,128]	3G5E	24.9% (164/658)	−11.30	TB2
	AMY2A	Catalyzes the hydrolysis of the α-1,4-d-glycobsidic bond of starch to produce glucose [129]	4GQR	8.7% (57/658)	−10.90	24-(*Z*)-3-oxolanosta-1,7,24-trien-26-oic acid
	FBP1	A major regulator that catalyzes glucose production in the second last step of gluconeogenesis [130]	2JJK	0%	-	
	GCK	Regulates glucose homeostasis and synthesizes glucose 6-phosphate from glucose in the glycolytic pathway [131]	3IMX	15% (98/658)	−10.40	6-cinnamylchrysin
	MGAM	Catalyzes the last step of starch digestion via hydrolysis of 1,4-α bonds in starch to produce glucose [132]	3L4Y	1.2% (8/658)	−9.80	24-(*Z*)-3-oxolanosta-1,7,24-trien-26-oic acid
	PDK2	Inhibits pyruvate dehydrogenase activity through a phosphorylation reaction which causes glycolytic disruption and glucose oxidation [131]	4MPC	0.8% (5/658)	−9.30	24-(*Z*)-3-oxolanosta-1,7,24-trien-26-oic acid
	PYGL	Catalyze the phosphorolysis of α-1,4-glycosidic bonds in glycogen to glucose-1-phosphat [133]	3DDS	0.6% (4/658)	−9.60	Acetoxymangiferonic acid
Regulation of lipid metabolism	NR5A2	Regulates the expression of genes involved in steroidogenesis, bile acid metabolism, and cholesterol synthesis [134]	4DOR	0.5% (3/658)	−9.70	Retusapurpurin A
	PPARA	Regulates the expression of genes involved in uptake, binding, and oxidation of fatty acids in the liver as well as lipoprotein assembly and lipid transport [135]	3FEI	1.2% (8/658)	−9.50	Isonymphaeol-B
	PPARD	Regulates fatty acid catabolism in skeletal muscle [135]	3PEQ	18.4% (121/658)	−11.80	Amphidinolide X
	PPARG	Regulates the expression of genes involved in adipogenesis and lipid metabolism, particularly fatty acid transport, lipid droplet formation, triacylglycerol metabolism, as well as lipolysis of triglycerides [135]	2FVJ	13.2% (87/658)	−12.00	Retusapurpurin A
	RXRA	Mediates gene transcription by forming heterodimers with PPAR [136]	1FM9	9.9% (65/658)	−12.00	Lespeol

Note: dipeptidyl peptidase-4 (DPP4), free fatty acid receptor 1 (FFAR1), dehydrogenase 1 (HSD11B1), hydroxysteroid 11-beta insulin receptor (INSR), protein tyrosine phosphatase non-receptor type 9 (PTPN9), retinol-binding protein 4 (RBP4), Aldose reductase (AKR1B1), pancreatic α-amylase (AMY2A), fructose-1,6-bisphosphatase (FBP1), glucokinase (GCK), intestinal maltase-glucoamylase (MGAM), pyruvate dehydrogenase kinase isoform 2 (PDK2), liver glycogen phosphorylase (PYGL), liver receptor homolog-1 (NR5A2), peroxisome proliferator-activated receptor α (PPARA), peroxisome proliferator-activated receptor delta (PPARD), peroxisome proliferator-activated receptor gamma (PPARG), retinoid X receptor α (RXRA).

This study used −9.0 kcal/mol as the cut-off docking score to distinguish between potential and non-potential antidiabetic compounds. Among all the test compounds, 275 compounds were observed for having a binding score lower than −9.0 kcal/mol and were considered as potential anti-T2DM compounds. The cut-off docking score set is deemed a reasonable average docking score covering the top 10% of test compounds for protein targets [20,137].

A total of 193 compounds could potentially bind to 2–11 T2DM protein targets, while 83 compounds act on a single target. Table 1 summarizes three categories of metabolism in T2DM. First, regulation of insulin secretion and sensitivity. There are 168 compounds were potential as dipeptidyl peptidase-4 (DPP4) inhibitors, whereas retusapurpurin A had the lowest docking energy (−10.9 kcal/mol). DPP4 is involved in inactivating glucagon-like peptide 1 (GLP-1), which plays an imperative role in the treatment of T2DM [119,138]. DPP4 is commonly used as a target for the treatment of T2DM. Retinol binding protein-4 (RBP4) and hydroxysteroid 11-beta dehydrogenase (HSD11B1) play an essential role in insulin resistance. A total of 82 Asian propolis compounds are potential inhibitors of RBP4, which could prevent insulin resistance. There are more than 20% of all Asian propolis compounds (137 of 658) were screened as potential inhibitors of HSD11B1. Second, regulation of glucose metabolism. AKR1B1 can prevent diabetes complications in T2DM by catalyze the reduction of glucose to sorbitol [128]. Notably, AKR1B1 can bind to 24.9% of all test compounds (164 of 658), and it had the highest percentage of all binding Asian propolis compounds to diabetes targets. Third, regulation of lipid metabolism, 87 propolis compounds bind to PPARG, 121 compounds bind to PPARD, and 65 compounds bind to RXRA. These proteins facilitate gene transcription by regulating lipids, insulin sensitivity, and glucose homeostasis. The proteins mentioned earlier are used as diabetes targets to treat T2DM [136]. PPAR protein isomer (PPARA, PPARD, PPARG) activity is interconnected to regulate other protein targets in different categories, such as GCK and HSD11B1 by PPARG and PDK2 by PPARD and PPARA [18].

**Figure 1:**
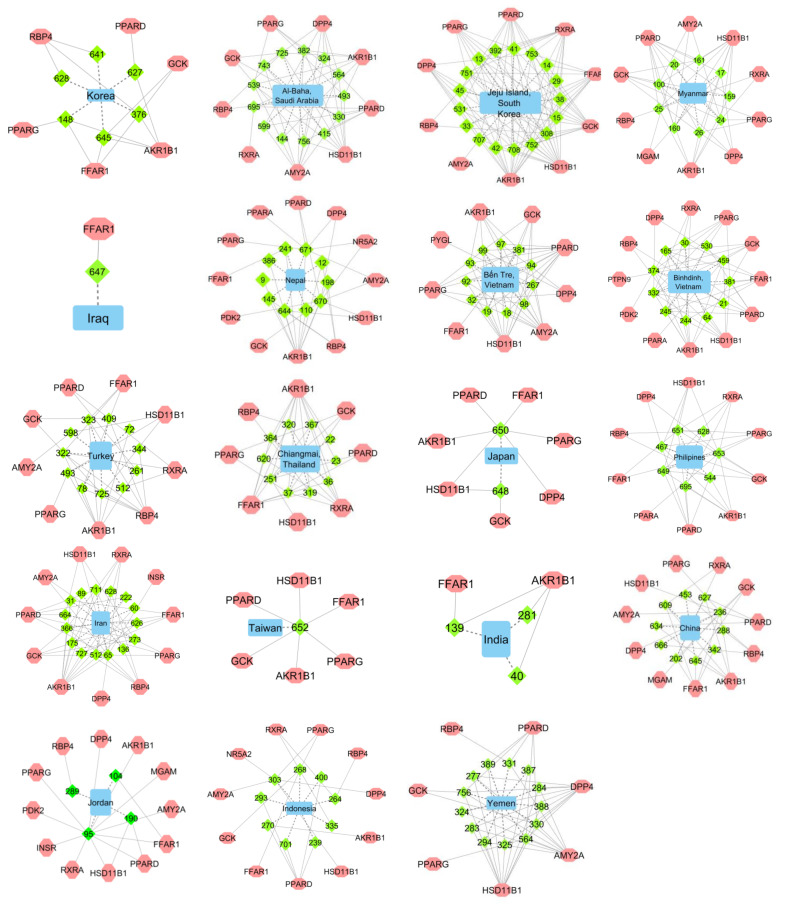
Nineteen Asian countries identified as propolis origins that produce propolis with novel potential anti-T2DM compounds. Potential compounds of propolis are represented by the number codes that are denoted with green diamond shapes (Table S2). Predicted T2DM targets are denoted with pink octagon shapes. The potential compounds have no previous literature on their antidiabetic potential. Dashed edges represent the edges connecting the propolis origin with their novel anti-T2DM compounds; solid edges represent the edge connecting the novel compounds with their predicted T2DM targets.

Figure 1 shows the origin of propolis compounds with their T2DM protein targets. There are 19 networks designed to identify Asian countries as a new rich source of anti-T2DM propolis compounds. The pink octagon shape denotes the T2DM target name, while the blue diamond shape denotes the code number of propolis compounds, as summarized in Table S2.

Propolis from China, Indonesia, Iran, Jeju Island–South Korea, Myanmar, Nepal, Philippines, and Vietnam can bind to more than 10 T2DM protein targets (Figure 1). Chinese propolis has been reported to inhibit the increase in fasting blood glucose and triglyceride levels in T2DM rats and improve insulin sensitivity, thereby controlling blood glucose level, lipid metabolism, and insulin sensitivity in T2DM rats [15]. In this study, we identified several novel Chinese propolis compounds that have antidiabetic activity, which are caffeic acid isoprenyl ester, pinobanksin-3-*O*-pentanoate *p*-coumaric acid benzyl ester, methoxy-cinnamic acid cinnamyl ester, 6-cinnamylchrysin, pinobanksin 3-*O*-hexanoate, and alpinetin. These compounds can bind to more than 10 T2DM protein targets, indicating that they undergo different cellular mechanisms.

On the other hand, triterpenoid compounds from Indonesian propolis have high inhibitory activity against α-glucosidase. These triterpenoid compounds include mangiferolic acid, cycloartenol, ambonic acid, mangiferonic acid, and ambolic acid [139]. The present study identified actinopyrone A, arisugacin E, 3-ketone, amphidinolide X, and acalycixeniolide K from the bee genera Trigona and 6-epiangustifolin, adhyperforin, and deoxypodophyllotoxin from the bee genus *Tetragonula* aff. *biroi*, and sulabiroins A and broussoflavonol from the bee genus Tetragonula [132,140] can bind to more than 10 T2DM protein targets.

In this study, Iranian propolis can bind to more than 10 T2DM protein targets. They are 10-hydroxybenzo[j]fluoranthene and 1,4-dihydrophenanthrene from *Apis mellifera* in Ardabil, Iran [48]; quercetin-7 methyl ether and pinobanksin 5,7-dimethyl ether from the source plants poplar and *Ferula ovina* [47], and pinobanksin 3-butanoate from brown propolis in Lalehzar Kerman province, Iran [49]. Furthermore, Iranian propolis has been tested in a randomized double-blind clinical trial in patients with T2DM [16]. The results showed that Iranian propolis could reduce HbA1C levels, post-prandial glucose levels, fasting blood glucose levels, serum insulin levels, insulin resistance, and levels of inflammatory cytokines.

### 2.2. Molecular Similarity Evaluation of Selected Propolis Compounds with Approved Anti-T2DM Drugs

**Figure 2:**
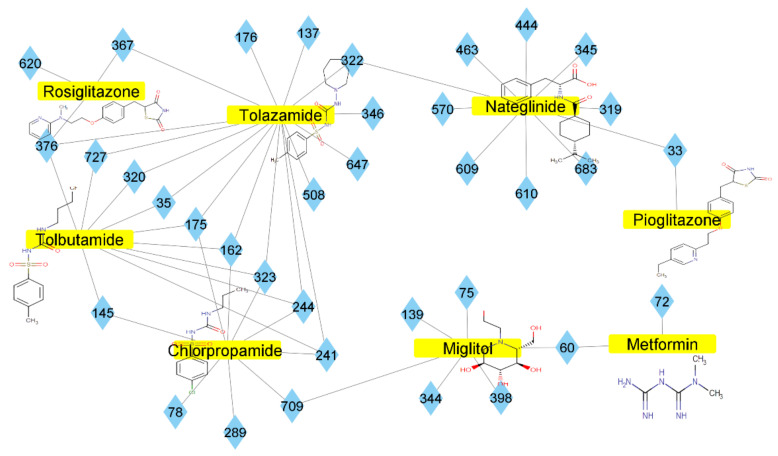
Molecular similarity analysis of potential anti-T2DM Asian propolis compounds to FDA-approved antidiabetic drugs. The similarity was performed on the extended connectivity fingerprint 4 (ECFP4) molecular fingerprints of compounds with a Tanimoto similarity cut-off score of 80%. Blue diamond shape denotes the number code of potential compounds that correspond to the compounds in Table S2.

Similarity of the selected compounds was evaluated using Tanimoto similarity analysis. In this study, we used eight FDA-approved drugs for treating T2DM, that is, chlorpropamide, metformin, miglitol, pioglitazone, nateglinide, tolazamide, tolbutamide, and rosiglitazone [141]. We found that 36 potential compounds of propolis were similar to eight FDA-approved anti-T2DM drugs (Figure 2). Interestingly, many of them are similar to other anti-T2DM drugs, namely, salsalate, vildagliptin, ipragliflozin, omarigliptin, and serotonin (Table 2) that have been reported to be novel agents for treating T2DM [142].

A total of nine potential compounds were similar to sulfonylurea drugs such as tolazamide, tolbutamide, and chlorpropamide (Figure 2). These compounds include phenylethyl *trans*-4-coumarate (175), benzyl cinnamate (323), 7,4′-dihydroxy-homo isoflavane (244), 6-hydroxy-3-methoxy-6-(3-phenyl-2-propenyl)-2-cyclohexane-1-one (241), and 3-deoxysappanol (162). The other two compounds from Iranian propolis and Turkish propolis, respectively, have similarities of more than 80% to metformin, namely, 1,4-dihydrophenanthrene (60) and 1-methyl-4-azafluorenone (72) [109,143]. Metformin is primarily used for the treatment of T2DM and is consumed by more than 150 million people each year owing to its high efficacy in therapy and affordable price. This FDA-approved drug has recently been evaluated for its complex mechanism of action through hepatic glucose production and metformin-associated AMPK activation [144,145].

Another anti-T2DM drug, pioglitazone, is a selective agonist of PPARG in target tissues for insulin action [145]. (*E*)-1-(5-Bromothiophen-2-yl)-3-[4-(dimethylamino)phenyl]prop-2-en-1-one (33) was the only compound with the closest similarity to pioglitazone (Table 2). The other ten compounds were observed to be similar to nateglinide, whereas neobavaisoflavone (570) and benzyl caffeate (322) were the most similar. Neobavaisoflavone is a potential active compound with seven T2DM targets: FFAR1, HSD11B1, PPARD, GCK, RBP4, PPARG, and RXRA (Table S3). The antidiabetic activity of neobavaisoflavone is unknown. However, the anti-SARS-CoV-2 activity of neobavaisoflavone through inhibition of the main protease (MPro) and spike glycoprotein (S2) of SARS-CoV-2 has been reported [115]. Our results indicate that neobavaisoflavone is an interesting compound that can bind to multiple anti-T2DM targets and is similar to an FDA anti-T2DM drug nateglinide.

### 2.3. Summary of Absorption, Distribution, Metabolism, Excretion, and Toxicity (ADMET) Parameters of Selected Propolis Compounds

ADMET analysis was performed to determine the physicochemical properties and oral bioavailability of propolis. This analysis is considered a cheap and time-saving alternative for screening potential therapeutic drugs compared to in vivo testing [146]. The Lipinski rules, also known as the rule of five (RO5), are the first consideration in this analysis, where a compound can be considered as an orally active if it fulfills or only violate one of these conditions: no more than five hydrogen bond (H-bond) donors, no more than 10 H-bond acceptors, molecular weight less than 500 g/mol, and the octanol-water coefficient is less than 5.0 [147]. In addition, as stated by the Veber rule, the compound has a good bioavailability for oral consumption if it has appropriate values for such as number rotatable bonds less than 10, topological polar surface area (TPSA) less than 140, and H-bond acceptor/donor less than 12 [148]. A compound that does not fulfill these criteria is considered to have poor absorption or permeation properties.

Table 3 lists that 90% of the potential compounds passed the rule of five (RO5) and Veber rules, which indicates the possibility of orally active compounds. The log S value indicates the solubility in an aqueous solution. As summarized in Table 3, 45.5% of the compounds have high solubility, while the other 54.5% have less [149,150]. This is not surprising, as many identified compounds in propolis are non-polar compounds such as terpenoids, sesquiterpenes, triterpene hydrocarbons, steroid hydrocarbons, aliphatic acids, aliphatic esters, fatty acids, and others.

Caco-2 permeability and human intestinal absorption are correlated with the predicted permeability through the major barrier intestinal epithelium and drug absorption. The permeability and HIA criteria were fulfilled by 74.5% and 95.6% of potential compounds, respectively. Meanwhile, the drug distribution is represented by PPB and the blood-brain barrier (BBB) permeability. Only 87 compounds of propolis were able to bind protein plasma. A total of 201 compounds are classified as BBB-positive, and these compounds can have neurodegenerative therapeutic applications.

A total of 60% of compounds had low acute toxicity (LD50), 54.2% did not affect the liver (hepatotoxicity), and 80.7% and 100% did not cause mutagenicity and carcinogenicity, as listed in Table 3. Moreover, 73.8% of compounds were identified as Ether-a-go-go Related-Gene (hERG) non-blockers. This is an essential property of the herbal compound, as the hERG blockade may induce “torsade de pointes” arrhythmias and sudden death. Our data show that most of the components of Asian propolis are safe for consumption. However, these data need to be validated by further studies at the in vitro and in vivo levels.

Only 12 potential compounds were found to have favorable ADMET properties (Table 4, Table 5 and Table 6). The physicochemical properties of the potential compounds are listed in Table 4, which shows no violated Lipinski’s and Veber’s drug-likeness. There were six compounds that were moderately soluble in water, based on log S less than 50 ug/mL, namely, (2*R*)-7,4′-dihydroxy-5-methoxy-8-methylflavane, (*RR*)-(+)-3′-senecioylkhellactone, alpinetin, chrysin, pinobanksin-3-*O*-butyrate, and pinocembrin-5-methyl ether, and the other six compounds were highly soluble in water [151]. Other parameters such as molecular weight, lipophilicity, H-bond acceptors, H-bond donors, rotatable bonds, and TPSA were within the acceptable range and met the criteria for potential compounds with favorable physicochemical properties [149].

Table 5 summarizes the pharmacokinetic properties of the compounds. Almost all 12 compounds are easily absorbed in the intestinal wall based on their HIA of more than 0.3 or >30% and their Caco-2 permeability of more than −5.15 log unit. Only catechin is not permeant to Caco-2 cells (−6.25) but can easily be absorbed in the human intestinal wall (HIA: 0.401). The plasma protein binding (PPB) properties of all compounds were more than 80%, and only (2*R*)-7,4′-dihydroxy-5-methoxy-8-methylflavane and catechin had protein-binding properties of more than 90%. This means that higher PPB probabilities would increase the protein-bound fraction of the compound in the human drug distribution [152].

There were no compounds with positive hepatotoxicity, AMES mutagenicity, or potential carcinogenicity, as listed in Table 6. All compounds have an LD50 of acute toxicity of more than 500 mg/kg, indicating that they have low toxicity properties. Only (*RR*)-(+)-3′-senecioylkhellactone had a low tumorigenic effect. In addition, all the compounds were non-tumorigenic. Therefore, these 12 potential compounds have favorable ADMET properties, suggesting the potential antidiabetic effects of Asian propolis compounds.

Table 7 shows 12 compounds with potential anti-T2DM activity and favorable ADMET properties for further drug design and development. There are six of twelve compounds that have been known as anti-diabetic agents, namely, naringenin, catechin, chrysin, hesperetin, pinocembrin, and sakuranetin. This in silico study revealed that the six known compounds have good inhibitory action to T2DM targets, namely, RBP4 and AKR1B1, which contribute in improving insulin sensitivity and prevent diabetes complication, respectively. Theses finding were match with previous reports of compounds. Sakuranetin alleviate the insulin response in diabetic patient and improve glucose hemeostatis [151,153], while naringenin and pinocembrin improve insulin sensitivity [154,155]. Chrysin ameliorate insulin resistance [156]. Moreover, clinical report of catechin supplementation showed an improving blood glucose level and recover insulin sensitivity in T2DM patients [157].

It should be noted that the other six compounds were firstly reported as potential anti-T2DM agents in this study, they are (*RR*)-(+)-3′-senecioylkhellactone; 2′,4′,6′-trihydroxy chalcone; (2*R*)-7,4′-dihydroxy-5-methoxy-8-methylflavane; pinobanksin-3-*O*-butyrate; alpinetin; and pinocembrin-5-methyl ether. (*RR*)-(+)-3′-senecioylkhellactone [91] is a South Korean propolis compound with potential inhibitory action on six anti-T2DM targets, while 2′,4′,6′-trihydroxy chalcone (pinocembrin chalcone) is a potential inhibitor of two anti-T2DM targets (Table S3). (2*R*)-7,4′-dihydroxy-5-methoxy-8-methylflavane; alpinetin; pinobanksin-3-*O*-butyrate; pinocembrin-5-methyl ether; and sakuranetin act on a single target of RBP4. RBP4 and AKR1B1 are major targets for all of the 12 potential compounds.

### 2.4. Analyzing the Molecular Docking Simulation Results of Asian Propolis Compounds with RBP4 and AKR1B1

**Figure 3:**
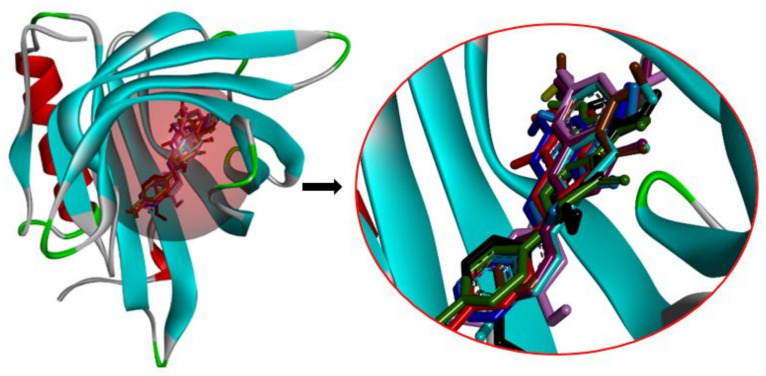
Binding positions of nine potential anti-T2DM Asian propolis compounds in Table 7 (RBP4) (PDB ID: 2WR6). The compounds are shown in various ligand colors; (color—number code of compound) red—21, blue—288, black—353, violet—359, light purple—482, tosca—482, brown—640, light blue—641, and dark green—681.

**Figure 4:**
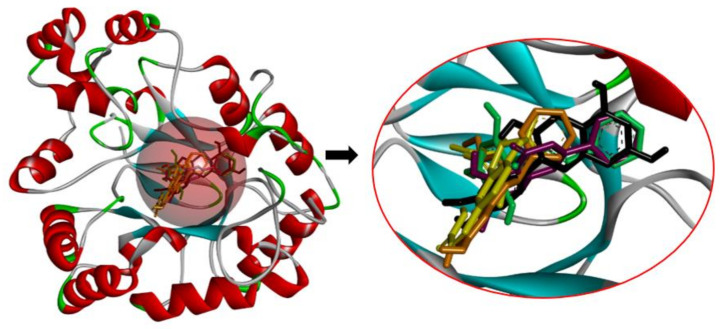
Binding positions of five potential anti-T2DM Asian propolis compounds in the active site of aldose reductase (AKR1B1) (PDB ID: 3G5E). The compounds are shown in various ligand colors; (color—number code of compound) yellow—41, green—51, black—353, violet—359, orange—633.

The predicted molecular interaction results of 12 potential Asian propolis compounds with two major targets (RBP4 and AKR1B1) are summarized in Table 8 with the number of amino acid residues in the active site that bind to the compound. Furthermore, the physical and chemical interactions between the compound and the respective target proteins were observed and analyzed. The table has only shown the potential compounds with a binding affinity lower than −9.0 kcal/mol to each primary target (Table 7), which means that the docking results of three ligands, namely, (*RR*)-(+)-3′-senecioylkhellactone; 2′,4′,6′-trihydroxy chalcone; and pinobanksin-3-*O*-butyrate for their interaction with RBP4 are not considered and discussed in this study, whereas the results for their interaction with AKR1B1 are discussed. According to Table 7; (2*R*)-7,4′-dihydroxy-5-methoxy-8-methylflavane and narigenin are the most potential Asian propolis compounds to inhibit RBP4 function with a binding affinity of −9.7 kcal/mol, followed by chrysin, hesperetin, pinocembrin, and sakuranetin with a binding affinity of −9.6 kcal/mol, while pinocembrin-5-methyl ether, alpinetin, and catechin have a lower binding affinity than the other six ligands at −9.4, −9.3, and −9.0 kcal/mol, respectively. The binding positions of all potential compounds to RBP4 and AKR1B1 are shown in Figure 3 and Figure 4, respectively. It was shown that all ligands bind to the target in a similar position, and this is related to the similar amino acid residues of several compounds in Table 8.

Based on our study, these 12 compounds are the most potent Asian propolis compounds as anti-T2DM agents against two major T2DM targets (RBP4 and AKR1B1). Retinol-binding protein 4 (RBP4) contributes to insulin resistance in T2DM. Thus, lowering RBP4 levels or reducing the activity of RBP4 could be a strategy for improving insulin sensitivity in T2DM [158]. Aldose reductase (AKR1B1) inhibitors have been evaluated for the management of diabetic complications [159]. Hesperetin and naringenin showed hydrogen bond interactions with key amino acid residues on the RBP4 binding site. The other five potential compounds showed a π-interaction with both key amino acid residues on the AKR1B1 binding site. (*RR*)-(+)-3′-Senecioylkhellactone and pinobanksin-3-*O*-butyrate form π-interaction to key residue of Phe122 and Tyr209. Meanwhile, (2*R*)-7,4′-dihydroxy-5-methoxy-8-methylflavane and naringenin bind to the same key residues in the active site of RBP4 and they have the lowest binding affinity than other Asian propolis compounds. The selected compounds were run to molecular dynamic simulation to analyze the stability of interaction.

### 2.5. Molecular Dynamic Simulations of Selected Asian Propolis Compounds

As the interaction between protein and the ligand in the docking simulation may not be stable under dynamic conditions, we performed molecular dynamics simulation to validate the stability of the interaction. This simulation generates a variety of particle trajectories, coordinates, velocities, and energies, which are then statistically analyzed to produce the result. The MD simulation was run for 40 ns to investigate the stability and conformational changes of the AKR1B1 and RBP4 structures when bound to the ligand. The Root Mean Square Deviation (RMSD), Radius Mean Square Fluctuation (RMSF), Radius of Gyration (Rg), and Solvent Accessible Surface Area (SASA) were all calculated.

**Figure 5:**
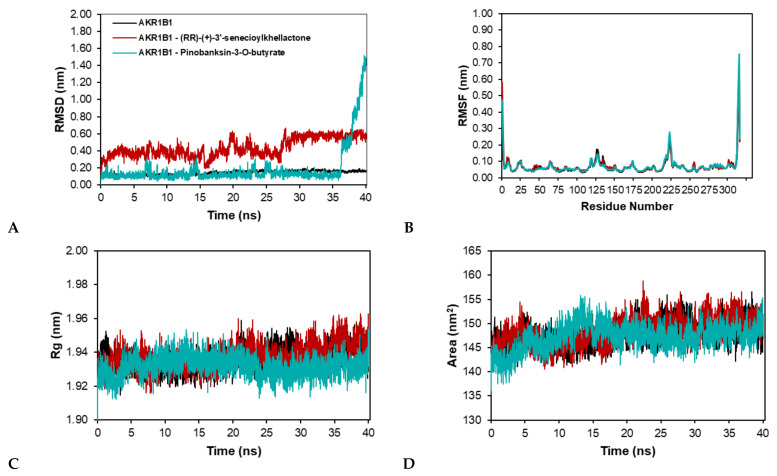
Molecular dynamic simulation trajectory analysis of AKR1B1, and AKR1B1-ligand complexes during 40 ns simulation. (**A**) RMSD of backbone atoms for AKR1B1-ligand complex. (**B**) RMSF of Cα AKR1B1-ligand complex. (**C**) The radius of gyration (Rg) of backbone atoms. (**D**) SASA of AKR1B1-ligand complex.

The RMSD value was investigated to assess the difference in frame deviation of the reference structure’s initial conformation and to measure differences between the structured samples and the reference structure during the simulation period [160]. A zero RMSD value indicates identical conformation structure, and a high value indicates dissimilarity [161]. As depicted in Figure 5A, except for the AKR1B1-pinobanksin-3-*O*-butyrate (pinobanksin) complex, the RMSD of protein AKR1B1 and the AKR1B1-(*RR*)-(+)-3′-senecioylkhellactone (senecioyl) complex are almost stable during molecular dynamic simulation. AKR1B1, AKR1B1-senecioyl, and AKR1B1-pinobanksin had mean RMSD values of 0.14, 0.44, and 0.18 nm, respectively. Although the average RMSD value of AKR1B1-pinobanksin-3-*O*-butyrate was lower than AKR1B1-senecioyl, the RMSD value of this protein–ligand complex rose to 1.4 nm after 36 ns.

The high RMSD value revealed the conformational change of the ligand. Meanwhile, the AKR1B1 and AKR1B1-senecioyl fluctuate less than 0.6 nm, as shown by the time plot of the RMSD values of the protein backbone. AKR1B1 and AKR1B1-senecioyl achieved stability with low RMSD fluctuation compared to AKR1B1-pinobanksin-3-*O*-butyrate. Thus, it suggests that AKR1B1 and AKR1B1-senecioyl were the most stable throughout the simulation.

The RMSF value revealed the flexibility of key residues throughout the trajectories. The RMSF profile of AKR1B1 and complexes was calculated using the Cα residue index. The fluctuation with max 0.23 nm located in Glu223 residue was found in the three systems, indicating flexible amino acid residue of AKR1B1. The highest instability in the AKR1B1-pinobanksin was found in residue Phe315 with an RMSF value of 0.75 nm, as shown in Figure 5B. Interestingly, the critical binding site residues Val47, Trp111, and Cys298 had stable fluctuation with RMSF < 0.2 nm for the entire 40 ns simulation. As a result, the findings strongly implied that the protein–ligand complexes were stable at their binding site.

Further radius gyration (Rg) analysis accounts for the complex compactness during simulation. The Low Rg value indicated the investigated molecule’s sustained compactness, with a plateau around the average [162,163]. As shown in Figure 5C, the AKR1B1 and AKR1B1-ligand complex Rg patterns are nearly identical. AKR1B1, AKR1B1-senecioyl, and AKR1B1-pinobanksin-3-*O*-butyrate had mean Rg values of 1.94, 1.94, and 1.93 nm, respectively. Despite having a slightly lower Rg value than AKR1B1 and AKR1B1-senecioyl, the RMSD value of this complex rose after a 36 ns simulation run.

The solvent-accessible surface area (SASA) was also calculated to analyze and measure the solvent behavior of the complexes [164,165]. Figure 5D shows that the average SASA value of ligand bound to AKR1B1 was 148 nm^2^ (senecioyl) and 147 nm^2^ (pinobanksin), while the AKR1B1 itself had a SASA value of 148 nm^2^. The SASA value of the AKR1B1-pinobanksin complex fluctuates rapidly at 14 ns and gradually stabilizes around 150 nm^2^ until the end of the simulation time. The AKR1B1 and AKR1B1-senecioyl fluctuate to almost stable around 150 nm^2^ during the simulation period.

**Figure 6:**
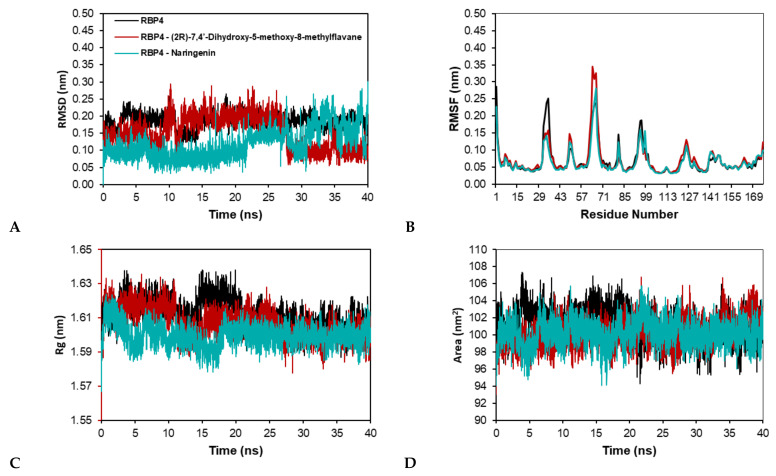
Molecular dynamic simulation trajectory analysis of RBP4, and RBP4-ligand complexes during 40 ns simulation. Molecular dynamic simulation trajectory analysis of RBP4-ligand complexes during 40 ns simulation. (**A**) RMSD of backbone atoms for RBP4-ligand complex (**B**) RMSF of Cα RBP4-ligand complex. (**C**) The radius of gyration (Rg) of backbone atoms. (**D**) SASA of RBP4-ligand complex.

During the RBP4 and RBP4 complexes simulation, as shown in Figure 6A, the RMSD value of RBP4-7,4′-dihydroxy-5-methoxy-8-methylflavane and RBP4-naringenin tend to fluctuate than RBP4. The RBP4 were almost stable in the whole simulation run with average RMSD value 0.19 nm. Meanwhile in the RBP4-7,4′-dihydroxy-5-methoxy-8-methylflavane, the protein–ligand complex fluctuates at ten ns with maximum RMSD values 0.29 nm, gradually decreases to stable after 28 ns with a mean RMSD of 0.15 nm. The RBP4-naringenin complex, on the other hand, highly fluctuates at the starting point of 28 ns with a maximum RMSD value of 0.29 nm. This complex has an average RMSD of 0.12 nm. Although the RBP4-naringenin underwent fluctuation during the periods between 10 and 28 ns, it is less volatile than RBP4-naringenin complex. These results also indicate that the RBP4 and RBP4-7,4′-dihydroxy-5-methoxy-8-methylflavane reached a steady state at the end of the simulation.

As seen in Figure 6B, the RMSF value of the RBP4 had fluctuation in the amino acid residue Leu35 with value 0.25 nm. Meanwhile in the RBP4-7,4′-dihydroxy-5-methoxy-8-methylflavane had greater fluctuation than the other 2 systems in the residue Leu64 (0.34 nm) and Asn66 (0.33 nm). Similar to RBP4-7,4′-dihydroxy-5-methoxy-8-methylflavane, the RMSF value of the RBP4-naringenin also had a large fluctuation in the residue Asn66 with values 0.28 nm.

The radius of gyration of the RBP4 and RBP4 complex is presented in Figure 6C. The Rg of the three systems shows a similar pattern with the fluctuations in the initial simulation and slowly downward until 17 ns. This fall trend also happens in the RBP4 and RBP4-7,4′-dihydroxy-5-methoxy-8-methylflavane around 16 ns and 14 ns. Meanwhile, in the RBP4-naringenin complex, the Rg shows a constant state after 18 ns. These complexes have average RMSD value 1.61 nm (RBP4), 1.60 (RBP4-7,4′-dihydroxy-5-methoxy-8-methylflavane), and 1.60 nm (RBP4-naringenin). However, the Rg value of the RBP4-naringenin shows a constant trend after 20 ns.

As shown in Figure 6D, the SASA of the RBP4 showed a slight increasing trend in the initial 4 ns simulation, then having a downfall to stable after 19 ns simulation time. However, in compare to the SASA pattern of RBP4-7,4′-dihydroxy-5-methoxy-8-methylflavane and RBP4-naringenin, these complexes tend to constant in the whole simulation time. RBP4, RBP4-7,4′-dihydroxy-5-methoxy-8-methylflavane, and RBP4-naringenin had average SASA values of 101 nm^2^, 100 nm^2^, and 100 nm^2^.

## 3. Materials and Methods

### 3.1. Collection of All Propolis Compounds in Asian Countries from All of the Previous Reports Related to Asian Propolis

Collection of the information on all propolis compounds in Asian countries was conduct-ed with Google Scholar (https://scholar.google.com, accessed on 13 July 2020), PubMed (https://pubmed.ncbi.nlm.nih.gov/, accessed on 14 July 2020), and ScienceDirect (https://www.sciencedirect.com/, accessed on 15 July 2020) using the following search terms: “propolis” together with Asian country names, for example “Chinese propolis”, “Indonesian propolis”, “Iranian Propolis”, “Malaysian propolis”, “South Korean propolis”, “Turkish propolis”, “Arabian propolis”, etc. There are 48 countries in Asia, and some do not report the bioactive components of propolis. Therefore, we collected information on all the original compounds of propolis from only 22 countries: Bangladesh, China, Cyprus, India, Indonesia, Iran, Iraq, Japan, Jordan, Leba-non, Malaysia, Myanmar, Nepal, Oman, Philippines, Saudi Arabia, South Korea, Taiwan, Thailand, Turkey, Uzbekistan, Vietnam, and Yemen. The total number of published papers correlated to the keywords was 100 papers. Then, we recorded all the chemical compositions of propolis in those papers together with their geographical origin, the solvent used in propolis extraction, and the extraction method (Table S1), whereas the same chemical compounds were eliminated. Last, 658 Asian propolis compounds were collected, complete with their chemical names.

### 3.2. Preparation of Compounds Structure and Inverse Virtual Screening of Potential Anti-T2DM Agents and Novel Compounds Analysis

The two-dimensional structure of compounds was obtained from the PubChem database (https://pubchem.ncbi.nlm.nih.gov/, accessed on 4 August 2020) and saved in sdf format. All the SMILES notation of compounds were recorded into data collection (Table S2). For compounds not found in Pubchem, we created their two-dimensional structure by using Advanced Chemistry Development (ACD)/ChemSketch freeware version 2019 (Advanced Chemistry Development, Inc, Toronto, ON, Canada) and generated their SMILES notations.

Structure based virtual screening (molecular docking) of 658 propolis compounds to diabetes targets was done by high performance computing (HPC) server via DIA-DB system in DIA-DB web server (https://bio-hpc.ucam.edu/dia-db/index.php, accessed on 2 September 2020). The SMILES notation of each compound (ligands) was submitted to the DIA-DB web server for inverse virtual screening [18,19]. These set of ligands were recorded in the main server and forwarded to database server. The database server registered the request for docking to the main server, which connected to HPC server. Then, molecular docking for inverse virtual screening was done in HPC server by using Autodock Vina [166,167]. A total of 658 Asian propolis compounds were docked against 18 T2DM targets: AKR1B1, DPP4, FBP1, GCK, HSD11B1, INSR, MGAM, PYGL, NR5A2, AMY2A, FPB1, PPARA, PPARD, PPARG, PTPN9, PDK2, RXRA, and RBP4 [18,19]. The high binding affinity of ligand to protein binding site indicated a potential anti-T2DM compound. Therefore, binding affinity of −9.0 kcal/mol was used to distinguish between potential and non-potential anti-T2DM compounds [20].

### 3.3. Similarity Studies of Potential Compounds with Approved Anti-T2DM Drugs

The known/experimental antidiabetic drugs were sourced from Defronzo et al. (2014) and Gougari et al. (2017) [141,142]. SMILES representations were obtained from PubChem software (National Center for Biotechnology Information, U.S. National Library of Medicine, Rockville Pike, MD, USA). The molecular similarity network was generated using the Cytoscape Application version 3.8.2 [168]. Molecular similarity was determined using the Tanimoto similarity metric on the calculated ECFP4 molecular fingerprints of the compounds. A Tanimoto score of 0.8 (80%) or greater was chosen as the threshold of molecular similarity.

### 3.4. Studies on Oral Bioavailability and Absorption, Distribution, Excretion, and Toxicity (ADMET) Properties of All Potential Compounds

The physicochemical descriptors of molecular weight, lipophilicity (XlogP), hydrogen bond acceptors, hydrogen bond donors, number of rotatable bonds, and topological polar surface area were calculated from SMILES code compounds using the Swiss ADME web server [48]. For ADMETLab, the three-dimensional structures of the compounds were gen-erated using Open Babel Gui version 2.3.1 (Chemistry Central Ltd., Pittsburgh, PA, USA) [149,169] and optimized with Data Warrior (Actelion Ltd., Allschwil, Switzerland) [170]. The data were then saved in sdf file format. ADMETLab was used to calculate solubility (LogS), caco-2 permeability, human intestinal absorption (HIA), protein plasma binding (PPB), blood–brain barrier (BBB), Ames mutagenicity, acute rat oral lethal dose 50 (LD50), hepatotoxicity, and HerG blockage [149]. Toxtree v3.1.0.1851 (Ideaconsult, Sofia, Bulgaria) and DataWarriors v05.02.01 (Actelion Ltd., Allschwil, Switzerland) were used to predict the potential carcinogens, tumorigenic compounds, reproductive effects, and irritants from the three-dimensional structures of potential compounds in sdf files [171]. All potential compounds that passed the acceptance criteria of all ADMET parameters, were further analyzed for their interaction with major T2DM targets.

### 3.5. Molecular Interaction Analysis among T2DM Targets and Potential Compounds of Propolis with Favorable ADMET Properties

The interaction between the target protein and potential compounds was determined using BIOVIA Discovery Studio 2020 (Dassault Systems, San Diego, CA, USA) [172]. Potential compounds with favorable ADMET properties were used to analyze the interactions at the binding site of the major protein targets. Molecular interactions are shown in 2D and 3D interactions.

### 3.6. Molecular Dynamic Simulation of Selected Compounds of Asian Propolis to Two Major Diabetes Targets

Molecular dynamics (MD) simulations were done using GROMACS 2020.6 [173]. Two ligands of each protein that has the lowest binding affinity were used in the simulation. The ligand and the corresponding protein were input in pdf format. The protein topology was generated in the Gromacspdb2gmx module using Charmm36 [174] and the ligand topology was generated in the SwissParam web server [175]. A cubic box was prepared for the protein–ligand complex, positioned at least 1.0 nm from the edge of the box. For the protein–ligand complex, a cubic box was prepared at least 1.0 nm from the edge of the box. Sodium and chloride were added to neutralize the charge. The steepest descent 4000 steps minimization algorithm is used to minimize the energy system. Then, the solvent and the ion were equilibrated in two restrained phases. The temperature used was 300 K for the NVT (isothermal-isochoric) ensemble, along with 1.0 bar pressure for the subsequent NPT (isothermal-isobaric) ensemble. The unrestrained 40 ns MD simulations were performed with the leap frog-integrator used with a step size of 2 fs. The LINCS (Linear Constraint Solver) algorithm was used to constrain the covalent bond and the electrostatic interaction was calculated using the Particle Mesh Ewald (PME) method [176,177].

## 4. Conclusions

Asian propolis was identified as an abundance compounds with potential anti-T2DM activity through inverse virtual screening using the DIA-DB web server. A total of 275 compounds from Asian propolis were found to be potential anti-T2DM agents because of their interaction with T2DM protein targets. A total of 192 compounds could bind to more than one T2DM target, indicating that Asian propolis has great potential as an anti-T2DM agent. Complex chemical compounds act on multiple diabetes targets in three main regulations of T2DM (regulation of glucose and lipid and insulin secretion/sensitivity). This action is superior to that of synthetic anti-T2DM drugs, which only act on a single target. A total of 12 potential compounds were identified as having favorable ADMET properties, and it should be noted that six compounds were identified as novel potential multiple-targeted anti-T2DM compounds, namely (2*R*)-7,4′-dihydroxy-5-methoxy-8-methylflavane; (*RR*)-(+)-3′-senecioylkhellactone; 2′,4′,6′-trihydroxy chalcone (pinocembrin chal-cone), alpinetin, pinobanksin-3-*O*-butyrate, and pinocembrin-5-methyl ether. RBP4 and AKR1B1 are major target of potential propolis compounds in T2DM.

(*RR*)-(+)-3′-senecioylkhellactone and pinobanksin-3-*O*-butyrat have stable interactions with the active site of AKR1B1 in dynamic condition. Moreover, (2*R*)-7,4′-dihydroxy-5-methoxy-8-methylflavane and naringenin also form stable binding in the active site of RBP4. Asian propolis compounds are good inhibitors of RBP4 levels, possibly decreasing insulin resistance in T2DM patients.

## Figures and Tables

**Table 2:** Summary of similarity analysis of selected Asian propolis compounds with diabetes drugs.

No.	Drug Name	Total Similar Compounds	Potential Compound with the Highest Similarity (Percentage of Similarity)	T2DM Target (Docking Score, kcal/mol)
1	Chlorpropamide 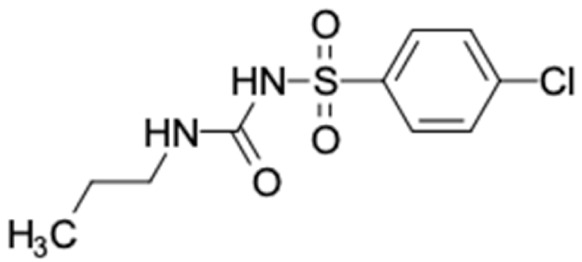	9	3′-Deoxysappanol (85.17%) 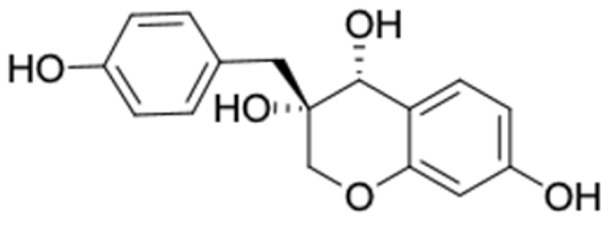	GCK (−9.4), AKR1B1 (−9.4)
2	Metformin 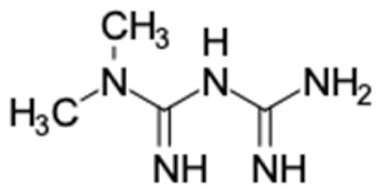	2	1,4-Dihydrophenanthrene (84%) 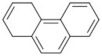	AKR1B1 (−10.3)
3	Miglitol 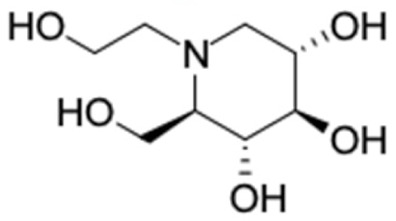	6	Calamenene (85.35%) 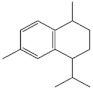	RBP4 (−9.0)
4	Pioglitazone 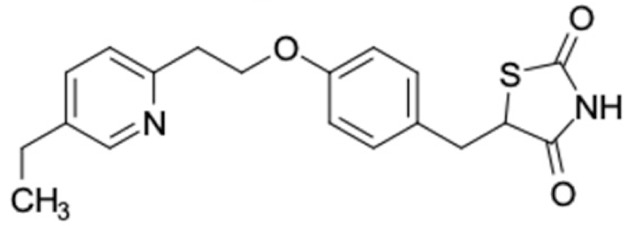	1	TB-5 (81.2%) 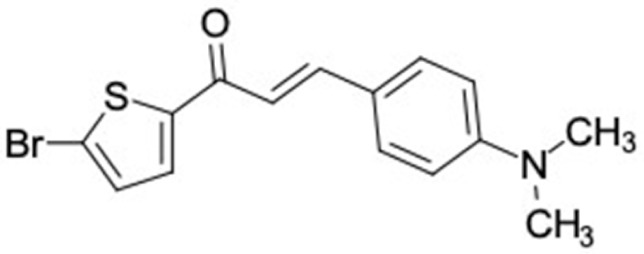	FFAR1 (−9.6), HSD11B1 (−9.4), DPP4 (−9.4), PPARD (−9.3), GCK (−10.0), AKR1B1 (−11.3), PPARG (−9.4), RXRA (−9.9)
5	Nateglinide 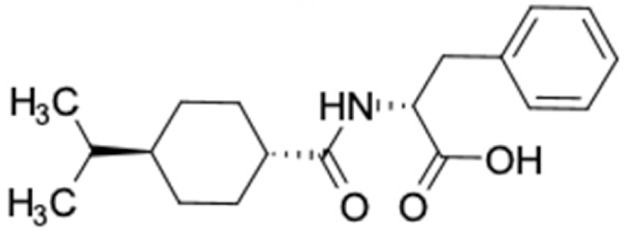	9	Neobavaisoflavone (84.4%) 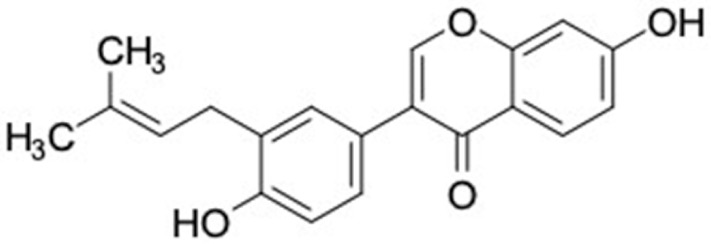	FFAR1 (−9.8), HSDB11B1 (−9.9), PPARD (−9.3), GCK (−9.9), RBP4 (−10.4), PPARG (−9.4), PPARG (−9.1)
6	Tolazamide 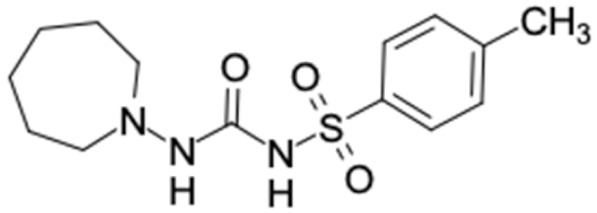	11	4-hydroxy-1-(2-hydroxyphenyl)-4-phenylbut-2-en-1-one (85.17%) 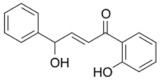	FFAR1 (−9.9), AKR1B1 (−9.5)
7	Tolbutamide 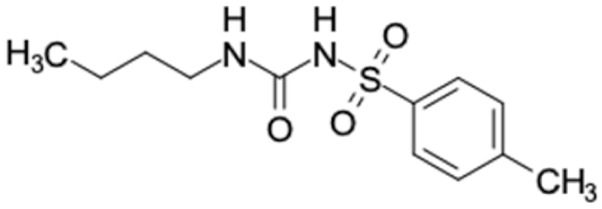	10	6-hydroxy-3-methoxy-6-(3-phenyl-2-propenyl)-2-cyclehexane-1-one (84.03%) 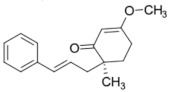	FFAR1 (−9.2), AKR1B1 (−9.4)
8	Rosiglitazone 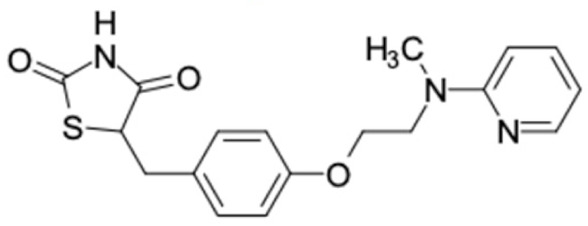	3	Phenethyl ferulate (81.5%) 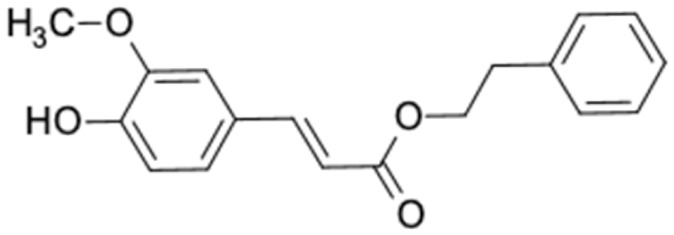	FFAR1 (−9.3), AKR1B1 (−9.8), RXRA (−9.3)
9	Gliclazide 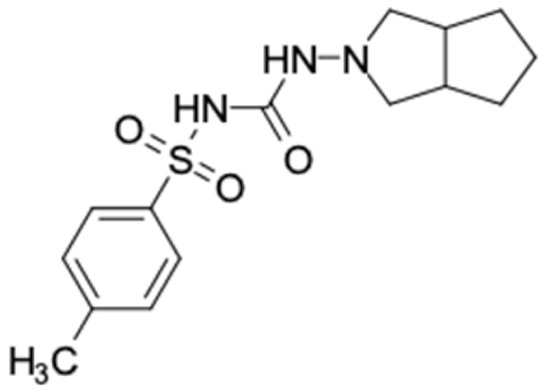	17	Benzyl caffeate (86.5%) 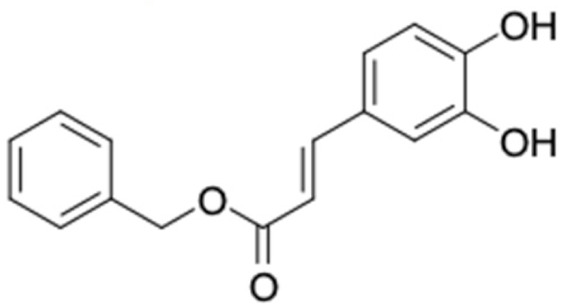	FFAR1 (−9.5), AKR1B1 (−9.8), RBP4 (−9.6), RXRA (−9.0)
10	Salsalate 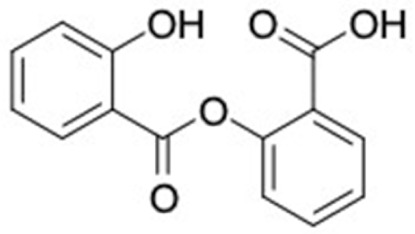	40	1-phenanthrenecarboxylic acid (87.84%) 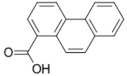	AKR1B1 (−10.1), RBP4 (−9.8)
11	Lipoic acid 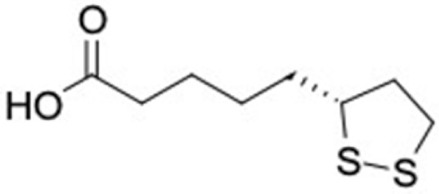	18	*trans*-chalcone (90.41%) 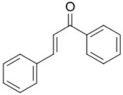	FFAR1 (−9.7), AKR1B1 (−9.4)
12	Vildagliptin 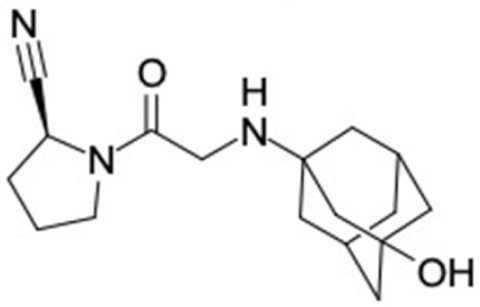	3	Tschimganine (84.18%) 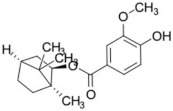	AKR1B1 (−9.3), RBP4 (−9.6)
13	Glymidine 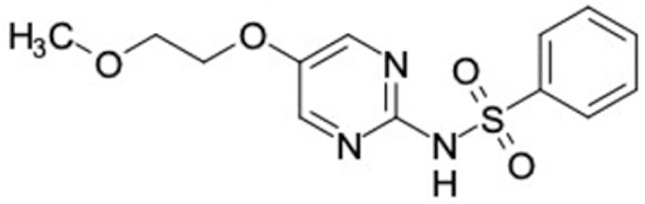	3	Phenethyl caffeate (81.12%) 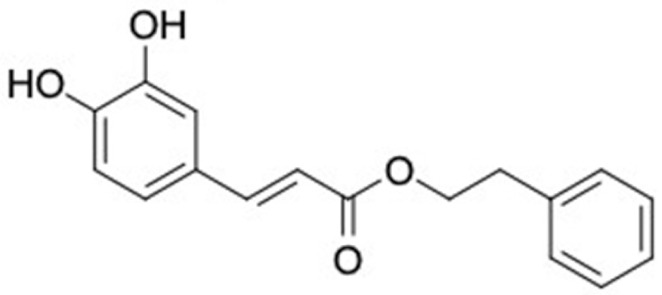	FFAR1 (−9.4), GCK (−9.2), AKR1B1 (−10.3), RBP4 (−9.8), RXRA (−9.1)
14	Ipragliflozin 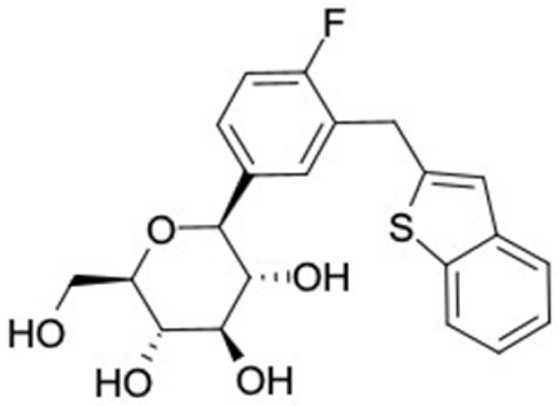	1	Phenethyl ferulate (81%) 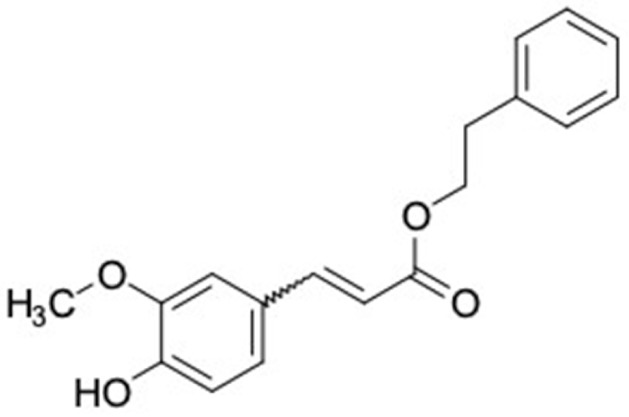	FFAR1 (−9.3), AKR1B1 (−9.8), RXRA (−9.3)
15	Voglibose 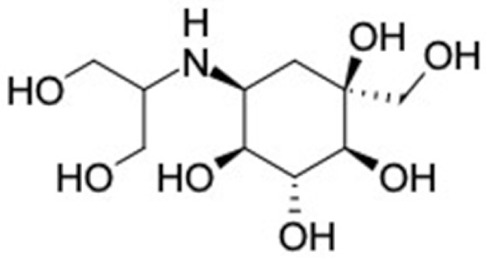	5	Delta-cadinene (84.54%) 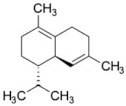	RBP4 (−9.0)
16	Omarigliptin 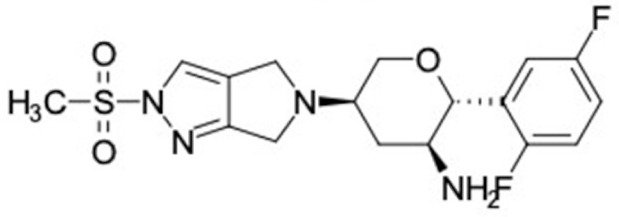	2	Rosmarinic acid (81%) 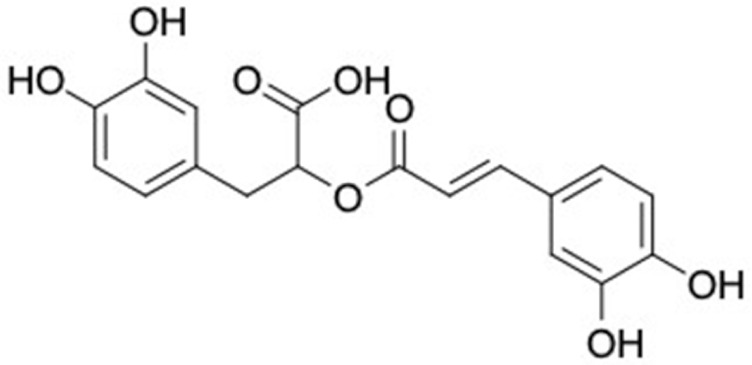	AKR1B1 (−10.5), PPARG (−9.1)
17	Serotonin 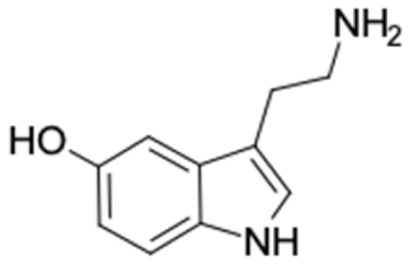	12	1,4-Dihydrophenanthrene (87.9%) 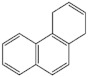	AKR1B1 (−10.3)
18	Mitiglinide 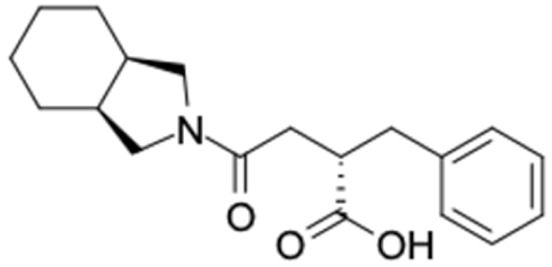	10	Ferulic acid benzyl ester (83.4%) 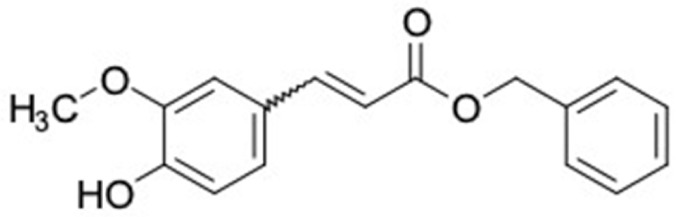	FFAR1 (−9.5), AKR1B1 (−9.7)

**Table 3:** Summary of ADMET parameters for potential compounds.

ADMET Property	Potential Compounds (%)	Ratio of Potential Compounds	Acceptable Range/Criteria
Lipinski’s rules	91.6	252/275	0–1 violation
Veber’s rules	92.4	254/275	0 violation
Solubility (Log S)	45.4	124/275	>10 µg/mL
Caco-2 permeability	74.5	205/275	>−5.15 log unit
Protein plasma binding	31.6	87/275	>90%
Blood–brain barrier (BBB)	73.1	201/275	Category 1: BBB+
Human intestinal absorption (HIA)	95.6	263/275	>30%; Category 1 HIA+
LD50 of acute toxicity	60.0	165/275	>500 mg/kg
Human hepatotoxicity (H-HT)	54.2	126/275	Category 0: H-HT negative
Ames Mutagenicity	80.7	222/275	Category 0: Ames negative
Potential carcinogen	100	275/275	No
hERG blockers	73.8	202/275	Category 0: Non-blockers

**Table 4:** Predicted physicochemical properties of potential compounds from Asian propolis.

Potential Compounds	Lipinski’s Rules (Violations)	Veber’s Rules (Violations)	MW (g/mol)	XLOGP3	H-Bond Acceptors	H-Bond Donors	Rotatable Bonds	TPSA (Å)	logS (µg/mL)
(2*R*)-7,4′-Dihydroxy-5-methoxy-8-methylflavane	0	0	286.32	3.44	4	2	2	58.92	16.36
(*RR*)-(+)-3′-senecioylkhellactone	0	0	218.33	3.94	1	1	1	20.23	35.65
2′,4′,6′-Trihydroxy chalcone (pinocembrin chalcone)	0	0	256.25	3.18	4	3	3	77.76	82.54
Alpinetin	0	0	270.28	2.65	4	1	2	55.76	32.57
Catechin	0	0	290.27	0.36	6	5	1	110.38	273.40
Chrysin	0	0	254.24	3.52	4	2	1	70.67	49.69
Hesperetin	0	0	302.28	2.60	6	3	2	96.22	94.93
Naringenin	0	0	272.25	2.52	5	3	1	86.99	83.94
Pinobanksin-3-*O*-butyrate	0	0	342.34	3.76	6	2	5	93.06	34.87
Pinocembrin	0	0	256.25	2.88	4	2	1	66.76	51.25
Pinocembrin-5-methyl ether	0	0	270.28	2.65	4	1	2	55.76	32.57
Sakuranetin	0	0	286.28	2.85	5	2	2	75.99	58.18

**Table 5:** Predicted pharmacokinetic properties of potential compounds from Asian propolis.

Potential Compounds	Caco-2 Permeability (Log Unit)	Human Intestinal Absorption	Blood–Brain Barrier	Protein Plasma Binding (PPB)%
(2*R*)-7,4′-Dihydroxy-5-methoxy-8-methylflavane	−4.854	+	+	90.46
(*RR*)-(+)-3′-senecioylkhellactone	−4.335	+	+	85.05
2′,4′,6′-Trihydroxy chalcone (pinocembrin chalcone)	−4.904	+	+	88.97
Alpinetin	−4.644	+	+	88.56
Catechin	−6.250	+	+	93.86
Chrysin	−4.973	+	+	89.57
Hesperetin	−4.876	+	+	88.06
Naringenin	−4.781	+	+	89.09
Pinobanksin-3-*O*-butyrate	−4.987	+	+	89.10
Pinocembrin	−4.882	+	+	87.44
Pinocembrin-5-methyl ether	−4.644	+	+	88.56
Sakuranetin	−4.830	+	+	89.56

**Table 6:** Predicted toxicity properties of potential compounds from Asian propolis.

Potential Compounds	LD50 Acute Toxicity (mg/kg)	Hepatotoxicity	Ames Mutagenicity	hERG Blockers	Tumorigenic	Potential Carcinogen Based on QSAR
(2*R*)-7,4′-Dihydroxy-5-methoxy-8-methylflavane	784.98	No	No	No	No	No
(*RR*)-(+)-3′-senecioylkhellactone	1535.08	No	No	No	low	No
2′,4′,6′-Trihydroxy chalcone (pinocembrin chalcone)	1975.49	No	No	No	No	No
Alpinetin	1293.66	No	No	No	No	No
Catechin	860.6	No	No	No	No	No
Chrysin	1054.98	No	No	No	No	No
Hesperetin	857.85	No	No	No	No	No
Naringenin	995.35	No	No	No	No	No
Pinobanksin-3-*O*-butyrate	599.05	No	No	No	No	No
Pinocembrin	1311.22	No	No	No	No	No
Pinocembrin-5-methyl ether	1293.66	No	No	No	No	No
Sakuranetin	872.56	No	No	No	No	No

**Table 7:** Potential compounds of Asian propolis with favorable ADMET properties.

Compound	Predicted Targets (Docking Score in kcal/mol)	Potential Antidiabetic Effect	Country of Origin
(2*R*)-7,4′-Dihydroxy-5-methoxy-8-methylflavane 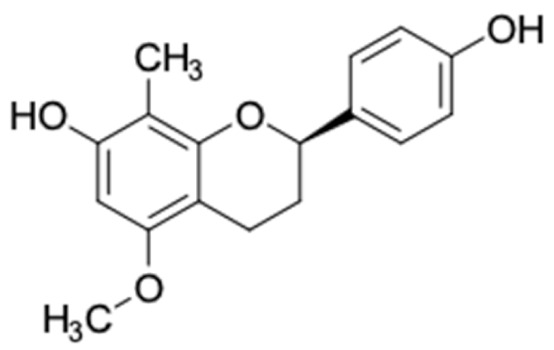	RBP4 (−9.7)	Regulation of insulin secretion and sensitivity	Vietnam
(*RR*)-(+)-3′-senecioylkhellactone 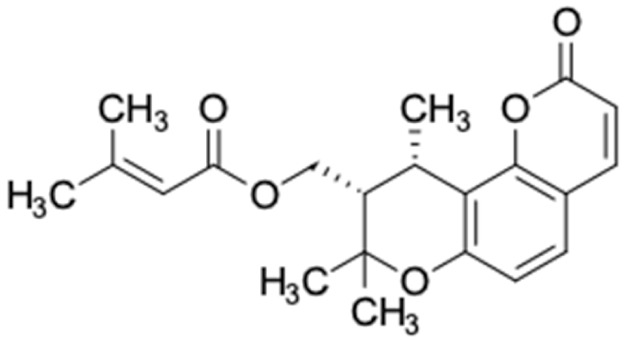	HSD11B1 (−9.5), PPARD (−10.0), GCK (−9.4), AKR1B1 (−9.4), PPARG (−9.1), RXRA (−9.4)	Regulation of insulin secretion and sensitivity, regulation of glucose and lipid metabolism	South Korea
2′,4′,6′-Trihydroxy chalcone (pinocembrin chalcone) 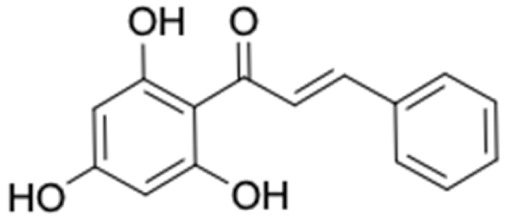	FFAR1 (−9.4), AKR1B1 (−9.0)	Regulation of insulin secretion/sensitivity and glucose metabolism	Iran
Alpinetin 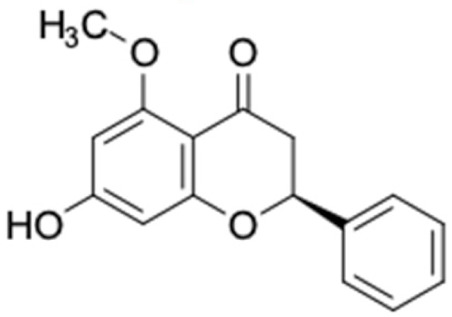	RBP4 (−9.3)	Regulation of insulin secretion and sensitivity	Beijing, China
Catechin 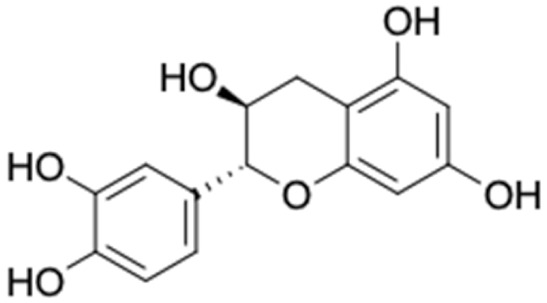	AKR1B1 (−9.0),RBP4 (−9.0)	Regulation of insulin secretion/sensitivity and glucose metabolism	China
Chrysin 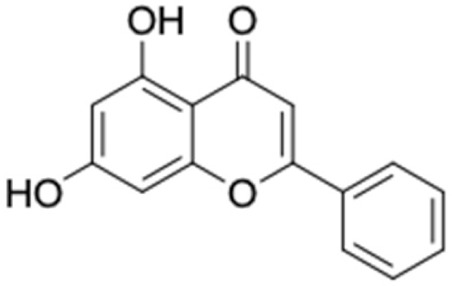	AKR1B1 (−9.0), RBP4 (−9.6), RXRA (−9.2)	Regulation of insulin secretion/sensitivity and glucose metabolism	Jordan, China, Thailand, Turkey, Iraq, Indonesia, Iran, Lebanon, South Korea, Uzbekistan
Hesperetin 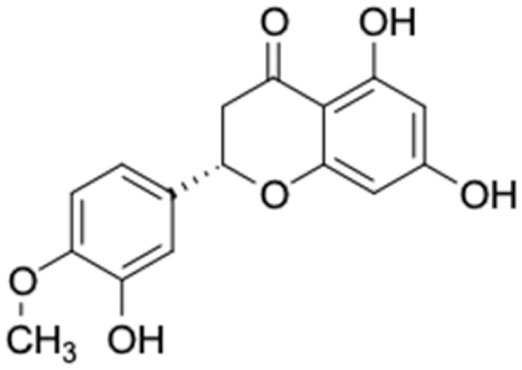	FFAR1 (−9.0), HSD11B1 (−9.3), RBP4 (−9.6)	Regulation of insulin secretion/sensitivity and glucose metabolism	Iraq, China
Naringenin 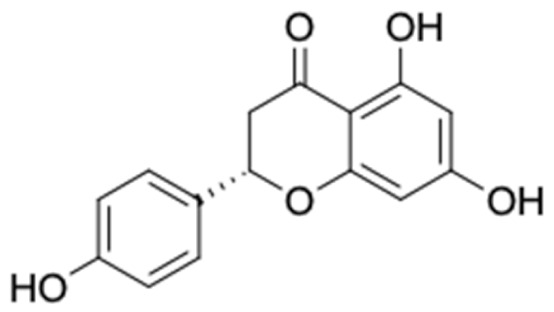	RBP4 (−9.7)	Regulation of insulin secretion/sensitivity	Jordan, Turkey, Iran, Iraq, China,
Pinobanksin-3-*O*-butyrate 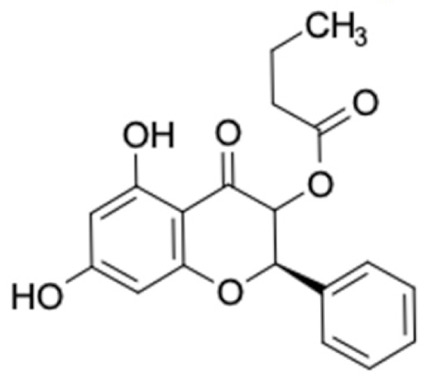	AKR1B1 (−9.0)	Regulation of glucose metabolism	China, Iran
Pinocembrin 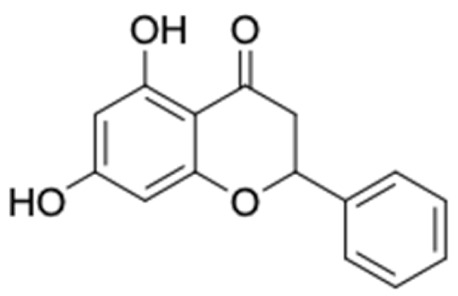	RBP4 (−9.6)	Regulation of insulin secretion/sensitivity	India, Lebanon, South Korea, Uzbekistan, Jordan, Iraq, Iran, China, Turkey, Nepal, Thailand
Pinocembrin-5-methyl ether 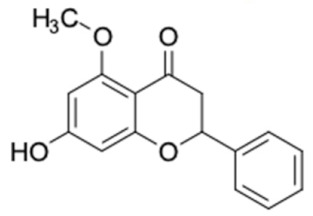	RBP4 (−9.4)	Regulation of insulin secretion/sensitivity	China
Sakuranetin 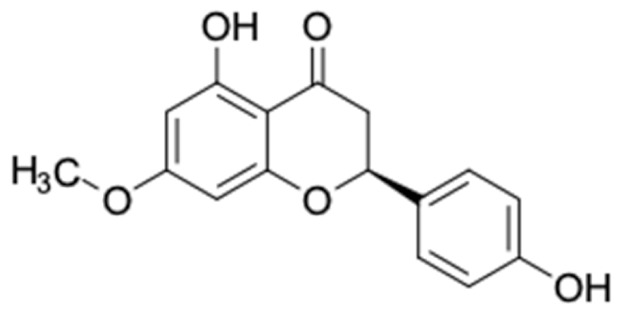	RBP4 (−9.6)	Regulation of insulin secretion/sensitivity	Iraq, Turkey, China

**Table 8:** Docking results of the interaction of 12 potentially active compounds of Asian propolis with favorable ADMET properties with protein targets related to T2DM.

Potential Compounds (Number Code)	RBP4	AKR1B1
(2*R*)-7,4′-Dihydroxy-5-methoxy-8-methylflavane (21)	Ala55 (C-H-bond), Arg121 (π-Cation), Met88 (π-Sulfur, Alkyl), Tyr90 (π-π T-Shaped, π-Alkyl interaction), Ala57 (π-Alkyl, Alkyl interaction), Leu37 (π-Alkyl, Alkyl interaction) Total: 6 residues	(Binding score > −9.0 Kcal/mol)
(*RR*)-(+)-3′-senecioylkhellactone (41)	(Binding score > −9.0 Kcal/mol)	Phe122 (π-π Stacked), Tyr209 (π-Sigma, π-Alkyl) Total: 2 residues
2′,4′,6′-Trihydroxy chalcone (pinocembrin chalcone) (91)	(Binding score > −9.0 Kcal/mol)	Cys303 (π-Sulfur), Cys80 (π-Sulfur), Leu300 (π-alkyl), Trp111 (π-π Stacked) Trp20 (π-π Stacked, π-π T-shaped), Cys298 (π-Sulfur) Total: 6 residues
Alpinetin (288)	Tyr133 (π-π Stacked), Arg121 (π-cation interaction), Pro32 (π-Alkyl) Leu37 (π-Alkyl), Met88 (π-sulfur), Ala57 (π-Alkyl, Alkyl interaction), Ala55 (Alkyl interaction) Total: 7 residues	(Binding score > −9.0 Kcal/mol)
Catechin (353)	Lys29 (H-bond), Pro32 (C-H bond), Tyr133 (π-π Stacked) Leu37 (π-Alkyl), Ala55 (π-Alkyl), Met88 (π-Sulfur) Ala57 (π-Alkyl) Total: 7 residues	Trp20 (π-π T-shaped), Cys80 (π-Sulfur), Leu300 (π-alkyl), Val47 (π-alkyl) Trp111 (π-π Stacked), Cys303 (π-alkyl) Total: 6 residues
Crysin (359)	Tyr90 (π-π T-shaped), Leu37 (π-Alkyl), Met73 (π-alkyl), Ala43 (π-alkyl), Ala57 (π-alkyl), Ala55 (π-alkyl), Met88 (π-sulfur) Total: 7 residues	Cys80 (π-sulfur), Cys303 (π-sulfur), Trp111 (H-Bond, π-π Stacked), Phe122 (π-π T-shaped), Leu300 (π-Alkyl) Total: 5 residues
Hesperetin (482)	Tyr90 (H-bond, π-π T-shaped), Arg121 (2 H-bond) Pro32 (Alkyl-interaction), Asp102 (π-Anion), Leu37 (π-Alkyl), Tyr133 (π-π Stacked), Ala43 (π-Alkyl) Ala57 (π-Alkyl), Ala 55 (H-bond, π-Alkyl) Total: 9 residues	(Binding score > −9.0 Kcal/mol)
Naringenin (568)	Arg121 (H-bond), Leu37 (π-Alkyl), Tyr90 (π-π T-shaped), Asp102 (π-anion) Thr56 (C-H Bond), Met88 (π-sulfur), Ala43 (π-alkyl), Ala55 (π-alkyl), Ala57 (π-alkyl) Total: 9 residues	(Binding score > −9.0 Kcal/mol)
Pinobanksin-3-*O*-butyrate (633)	(Binding score > −9.0 Kcal/mol)	Phe122 (π-π stacked), Val47 (H-bond, π-Alkyl), Trp20 (C-H bond, π-π stacked, π-Alkyl), Cys298 (H-bond), Tyr209 (π-sigma), Trp111 (H-bond, π-π stacked), Leu300 (π-alkyl) Total: 7 residues
Pinocembrin (640)	Arg121 (π-cation), Leu37 (π-alkyl), Tyr90 (π-π T-Shaped), Ala55 (π-alkyl), Ala43 (π-alkyl), Ala57 (π-alkyl), Met88 (π-sulfur) Total: 7 residues	(Binding score > −9.0 Kcal/mol)
Pinocembrin-5-methyl ether (641)	Pro32 (π-alkyl), Leu37 (π-alkyl), Arg121 (π-cation), Met88 (π-sulfur), His104 (C-H bond), Ala57 (π-alkyl, alkyl interaction), Ala55 (C-H bond), Tyr133 (π-π stacked) Total: 7 residues	(Binding score > −9.0 Kcal/mol)
Sakuranetin (681)	Lys29 (H-bond), Phe137 (π-alkyl), Ala57 (π-alkyl), Phe45 (π-alkyl), Ala43 (Alkyl interaction), Arg121 (π-cation), Leu37 (π-alkyl), His104 (π-alkyl), Met88 (π-sulfur) Total: 9 residues	(Binding score > −9.0 Kcal/mol)

## Data Availability

The data presented in this study are available within the article.

## Supplementary Materials

The following supporting information can be downloaded at: https://www.mdpi.com/article/10.3390/molecules27133972/s1, Table S1: Chemical composition of Asian Propolis, Table S2: SMILES codes of all test compounds, Table S3: Potential compounds of Asian propolis with protein targets and source countries.
