# Supplementary material for: Computational Study of Asian Propolis Compounds as Potential Anti-Type 2 Diabetes Mellitus Agents by Using Inverse Virtual Screening with the DIA-DB Web Server, Tanimoto Similarity Analysis, and Molecular Dynamic Simulation
Source: Molecules. 2022 Jun 21;27(13):3972. doi: 10.3390/molecules27133972 (PMC9268573; doi:10.3390/molecules27133972)
Supplement: Supplementary file 1 [file molecules-27-03972-s001.zip › molecules-1748383-supplementary.pdf]

SUPPLEMENTARY MATERIALS

Computational Study of Asian Propolis Compounds as Potential Anti-Type 2 Diabetes Mellitus Agents by Using Inverse Virtual Screening with the DIA-DB Web Server, Tanimoto Similarity Analysis, and Molecular Dynamic Simulation

Table S1. Chemical Composition of Asian Propolis

No.	Geographical Origin	Propolis Extract	Compound identified	Analytical method	References
**1.**	Manikgonj, Dhaka division, Bangladesh	Ethanolic extract	Tannic acid, gallic acid, (+)(-)catechin, pyrogallol, naringin, vanillic acid, rutin, benzoic acid, trans-cinnamic acid, quercetin, salicylic acid	HPLC-UV	[1]
**2.**	China	Ethanolic extract	protochatechuic acid, chlorogenic acid, gentistic acid, caffeic acid, rutin, p-coumaric acid, quercetin 3-O-galaktoside (Hyperoside), ellagic acid, apigenin 7-O-apioglucoside (Apiin), ferulic acid, kaempferol 3-O-glucoside (Astragalin), Luteolin 7-O-glucoside (Cynaroside), Apigenin 7-O-glucoside (Apigetrin), resveratrol, hesperetin, naringenin, apigenin, genistin, kaempferol, chrysin, pinocembrin, galangin	UHPLC-MS	[2]

**3.**	Hebei, China<br>Shanxi, China<br>Beijing, China<br>Henan, China<br>Shandong, China<br>Shaanxi, China<br>Gansu, China<br>Jiangsu, China<br>Ningxia, China<br>Anhui, China<br>Heilongjiang, China<br>Jilin, China<br>Liaoning, China	Ethanolic Extract	Chrysin, Apigenin, Kaempferol, Galangin, Pinocembrin,Pinostrobin, Cinnamic acid, 3,4-Dimethoxycinnamic acid, p-Coumaric acid, Caffeic acid, Ferulic acid, isoFerulic acid, Cinnamyl caffeate, benzyl caffeate, vanilin, phenethyl caffeate	H NMR	[3]
**4.**	China	Ethanolic Extract	chlorogenic acid, caffeic acid, isochlorogeni A, isochlorogenic acid C, myricetin, querce kaempferol, apigenin, pinocem- brin, caffei phenethyl ester, galangin, artepillin C	HPLC-UV	[4]
**5.**	Raohe, Heilongjiang, China<br>Mudanjiang, Jinlin, China<br>Shijiazhuang, Hebei, China<br>Cangzhou, Hebei, China<br>Zhongwei, Ningxia,	Ethanolic extract or Water extract	O-Xylene, Benzoic acid, D-Limonene, β-Cedrene, Tiglic-acid, Hexanoic acid, 3-Phenyl-1-propanol, α-Bisabolol, Linalool, δ-Cadinene, Cinnamyl alcohol , α-Eudesmol, Palmitic acid, P-Xylene, (E)-Cinnamaldehyde, Octanal, Decanal, nonanal, α-Cedrene, β-Eudesmol, Acetophenone, 3,4-Dimethoxystyrene, 2-Methoxy-4-vinylphenol, α-Curcumene,	GC-MS	[5]

China	Benzyl benzoate, Phenethyl alcohol, Benzaldehyde, Benzyl alcohol, Cedrol, γ-Eudesmol, Guaiol
Jining, Shandong, China	
Tianshui, Gansu, China	
Yili , Xinjiang, China	
Wulanhaote, Neimenggu, China	
Huzhu, Qinghai, China	
Xianyang, Shanxi, China	
Xi’an, Shanxi, China	
Nanjing, Jiangsu, China	
Longchang, Sichuan, China	
Huangshan, Anhui, China	
Zunyi, Guizhou, China	
Zunyi, Guizhou, China	
Yongzhou, Hunan, China	
Fuzhou, Fujian, China	
Lechang, Guangdong, China	
Lijiang, Yunnan, China	

	Maoming, Guangdong, China Xishuangbanna, Yunnan, China				
**6.**	Fuzhou, Fujian, China	Ethanol Extract	p-Hydroxybenzoic acid, Caffeic acid, p-Coumaric acid, Isoferulic acid, Apigenin-7-O-β-D-glucopyranoside, Kaempferol-methyl ether, Pinobanksin-methyl ether (b) Quercetin-dimethyl ether, Pinobanksin-5-methyl ether, Luteolin, Quercetin, Pinobanksin-methyl ether isomer, Quercetin-3-methyl ether, Chrysin-5-methyl ether, Pinobanksin, Kaempferol, Pinocembrin-5-methyl ether, Isorhamnetin, Kaempferol-3-methyl ether, Quercetin-dimethyl ether isomer, Galangin-5-methyl ether, Pinobanksin-5-methyl ether-3-O-acetate, Kaempferol-7-methyl ether, Quercetin-dimethyl ether isomer, Quercetin-dimethyl ether isomer,Caffeic acid isoprenyl ester, Quercetin-trimethyl ether, Chrysin, Caffeic acid isoprenyl ester isomer, Caffeic acid benzyl ester, Pinocembrin, Galangin, Pinobanksin-3-O-acetate, Phenethyl caffeate, Acacetin, p-Coumaric acid benzyl ester, Pinobanksin-3-O-	UPLC-ESI-MS	[6]

			acetate-5-O-phydroxyphenylpropionate, Caffeic acid cinnamyl ester, Pinobanksin-3-O-propionate, p-Coumaric acid benzyl ethyl ester, p-Coumaric cinnamyl ester, Pinobanksin-3-O-butyrate, Pinobanksin-3-O–pentanoate, Pinobanksin-3-O-acetate-5-O-phydroxyphenylpropionate, Pinobanksin-3-O-benzoate, Pinobanksin-3-O-pentanoate, 2-methylbutyrate, Pinobanksin-O-hexenoate, Pinobanksin-3-O-phenylpropionate, Pinobanksin-3-O-hexanoate		
**7.**	Jixi, Heilongjiang, China Mudanjiang, Heilongjiang, China Shuangyashan, Heilongjiang, China Qiqihaer, Heilongjiang, China Qitaihe, Heilongjiang, China Haerbin, Heilongjiang, China ; Jiilin, Jilin Jiilin, China Dunhua, Jilin, China	Ethanol extract	caffeic acid, p-coumaric acid ferulic acid, isoferulic acid, 3,4-dimethoxycinnamic acid pinobanksin, naringenin quercetin, kaempferol apigenin, pinocembrin benzyl caffeate, 3-O-acetylpinobanksin chrysin, CAPE galangin	HPLC and HPLC-ESI/MS Analysis	[7]

**8.**	China	Ethanol extract	Kaempferol, Fumaric acid, Pyrogallol, p-OH benzoic acid,Caffeic acid, t-ferulic acid, Quercetin, Ellagic acid, Isorhamnetin, Chlorogenic Acid, Rosmarinic acid, Rutin Hydrate, Gallic acid	LC-MS/MS	[8]
			Galangin, Chrysin, Pinobanksin, Pinocembrin, Pinostrobin, Apigenin, Luteolin, t-cinnamic acid, Quercetin dihydrate	HPLC	
**9.**	Shandong, China	Water extract	Protocatechuic acid, Vanillic acid, Caffeic acid, Syringic acid, 7-Hydroxycoumarin, P-Coumaric acid, Ferulic acid, Isoferulic acid, Rutin, 3,4-Dimethoxycinnamic acid, Myricetin, Trans-cinnamic, Quercetin, Pinobanksin, Luteolin, Kaempferol, Apigenin, Pinocembrin, Chrysin, CAPE, Galangin, Curcumin, Artepillin C, α-Mangostin	HPLC-DAD/Q-TOF-MS analysis	[9]
**10.**	Shandong, China	Ethanol extract	3,4-dimethoxycinnamic acid, pinobanksin, benzyl caffeate, chrysin, caffeic acid phenethyl ester (CAPE), cinnamyl caffeate	HPLC	[10]

**11.**	Shandong, China	Ethyl acetate extract	caffeic acid, p-Coumaric acid, ferulic Acid, isoferulic acid, 3,4-dihydroxybenzoic acid, trans-Cinnamic acid, phenethyl caffeate, apigenin, chrysin, quercetin, kaempferol, galangin, pinocembrin, pinobanksin, luteolin	HPLC-DAD/Q-TOF-MS analysis	[11]
**12.**	Shandong, China	Ethanol extract	Vanillic acid, Caffeic acid, p-Coumaric acid, Ferulic acid, Isoferulic acid, 3,4-Dimethoxycinnamic Rutin, Cinnamic acid, Myricetin, Pinobanksin, Naringenin, Quercetin, Luteolin, Kaempferol, Apigenin, Pinocembrin, 3-O-acetylpinobanksin, Chrysin, Caffeic acid phenylethyl ester, Galangin.	HPLC	[12]
**13.**	Lianhu District, Xian, China	Methanol Extract and Ethanol extract	cinnamyl alchol, cinnamic acid, 4-hydrocinnamic acid, 3,4-dimethoxy cinnamic acid, 4-hydroxycinnamic acid, caffeic acid, ferulic acid, pinostrobin, naringenin, sakuranetin, pinobanksin, 3-O-acetyl pinobanksin, chrysin, tectochrysin, genkwanin, galangin, kaempferol, isorhamnetin	GC-MS and LC-GC-MS	[13]
**14.**	Shandong, China	water extract	caffeic acid, ferulic acid, isoferulic acid, 3,4-dimethoxy cinnamic acid,	HPLC	[14]

			pinobanksin, caffeic acid benzyl ester, caffeic acid phenethyl ester, apigenin, pinocembrin, chrysin, galangin		
**15.**	Shandong, China	Ethanol extract	Vanillic acid, caffeic acid, p-coumaric acid, ferulic acid, isoferulic acid, 3,4-dimethoxycinnamic acid, rutin, cinnamic acid, pinobanksin, naringenin, quercetin, luteolin, kaempferol, apigenin, pinocembrin, 3-O-acetylpinobanksin, chrysin, CAPE, galangin, Chlorogenic acid, kaempferide, artepillin	HPLC	[15]
**16.**	Shandong, China	Water extract	caffeic acid, ferulic acid, isoferulic acid, 3,4-dimethoxycinnamic acid, pinobanksin, caffeic acid benzyl ester, caffeic acid phenethyl ester, apigenin, pinocembrin, chrysin, galangin.	HPLC	[16]
**17.**	Taishan Mountain, Shandong, China Huangshan, Anhui, China	Ethanol extract	gallic acid, catechin, epicathecin, caffeic acid, alpha catechin, p-coumaric acid, ferulic acid, rutin, myricetin, 3,4-dimethoxycinnamic acid, fisetin, morin, cinnamic acid, quercetin, naringenin, luteolin, genistein, kaempferol, apigenin, isorhamnetin, baicalin, pinocembrin, chrysin	RP-HPLC	[17]

**18.**	17 PROVINCE IN CHINA	Ethanol extract<br>Phenolic extract	Caffeic acid, p-Coumaric acid, Ferulic acid, Isoferulic acid, 3,4-dimethoxycinnamic acid, Cinnamic acid, Pinobanksin, Naringenin, Quercetin, Kaempferol, Apigenin, Pinocembrin, 3-O-Acetyl pinobanksin, Chrysin, CAPE, Galangin	HPLC	[18]
**19.**	Shandong, China	Ethanol extract	Rutin, myricetin, quercetin, kaempferol, apigenin, pinocembrin, chrysin, galangin	HPLC	[19]
**20.**	Shandong, Jilin, Anhui, Zhejiang, Jiangsu, Jiangxi and Henan provinces of China		galangin, chrysin	HPLC	[20]
**21.**	China	Methanol extract	Quercetin, pinobanksin-5-methyl ether, quercetin-3-methyl ether, apigenin, pinobanksin, isorhamnetin, quercetin-X-methyl ether, pinocembrin-5-methyl ether, pinocembrin-5-methyl ether, luteolin-5-methyl ether, quercetin-5,7-dimethyl ether, galangin-5-methyl ether, quercetin-7-methyl-x-methyl ether, chrysin, caffeic acid isoprenyl ester, pinobanksin-7-methyl-ether,	UPLC-Q-TOF-MS	[21]

			pinocembrin, galangin, pinobanksin-3-o-acetate, cape, hydroxy-cinnamic acid benzyl ester, p-coumaric acid benzyl ester, pinobanksin-3-o-propionate, chrysin-7-methyl ether, p-mehtoxy-cinnamic acid cinnamyl ester, pinobanksin-3-o-(butyrate-or isobutyrate), pinobanksin-3-o-(pentanoate or 2-methylbutyrate), methoxycinnamic acid cinnamyl ester		
**22.**	Anhui, China	chloroform, ethyl acetate and *n*-butanol extract	caffeic acid, (E)-p-coumaric acid, isoferulic acid, 3,4-dimethylcaffeic acid, pinobanksin-5-methyl ether, cinnamic acid, 4-methoxycinnamic acid, pinobanksin, rhamnocitrin, isopent-3-enyl caffeate, 3,3-dimethylallyl caffeate, 2-methyl-2-butenyl caffeate, chrysin, pinocembrin, galangin, phenethyl caffeate, cinnamyl caffeate, benzyl caffeate, quercetin-3,3'-dimethyl ether, tectochrysin, 3-acetylpinobanksin-7-methyl ether, galangin-7-methyl ether	HPLC	[22]
**23.**	Maoming, Guangdong, China Xishuangbanna,	Methanol extract	Ethyl acetate, Pentanal, α-Pinene, Toluene, 2-Methyl-3-buten-2-ol, Hexanal, Camphene, Butyl acetate Ethyl butanoate,	GC-O and GC-MS	[23]

Yunnan, China<br>Lechang, Guangdong, China<br>Lijiang, Yunnan, China<br>Fuzhou, Fujian, China<br>Fuzhou College of Bee Science, Fujian, China<br>Yongle, Hunan, China<br>Zunyi-Loudi, Guizhou, China<br>Zunyi-Shibangqiao, Guizhou<br>Longchang, Sichuan, China<br>Huangshan, Anhui, China<br>Nanjing, Jiangsu, China<br>Xi'an, Shannxi, China<br>Tianshui, Gansu, China<br>Jining, Shandong, China<br>Hui Nationality Autonomous Region, Zhongwei, Ningxia<br>Shannxi, China	3-Carene, β-Myrcene D-Limonene, β-Phellandrene, Eucalyptol, Ethyl hexanoate, 3-Methyl-1-butanol, Styrene, 3-Methyl-3-buten-1-ol, Octanal, 2-Methyl-2-buten-1-ol 21 1-Pentan-3-ol, 1-Hexanol, Dipropyl disulfide, Nonanal, Acetic acid, 1-Octen-3-ol, Furfural, α-Cubebene, Propanoic acid, Benzaldehyde, Dihydro-5-methyl-2(3H)-furanone, Butanoic acid, 1,2,3,5,6,8a-Hexahydro-4,7-dimethyl-naphthalene, butyrolactone, Naphthalene, 2-Hydroxy-benzoic acid formate, 2-Phenylethyl acetate, 2-Methyl-2-butenoic acid ,1-Methyl-naphthalene, Benzyl alcohol, Phenylethyl alcohol, Phenol, Guaiol

	Changzhou, Hebei<br>Shijiazhuang, Hebei<br>Yili, Xinjiang<br>Jilin, Jilin<br>Huzhu, Qinghai, China<br>W Raohe, Heilongjiang, China				
**24.**	Changge, Henan, China	Ethanol extract	8-[(E)-4-phenylprop-2-en-1-one]-(2R,3S)-2-(3,5-dihydroxyphe- nyl)-3,4-dihydro-2H-2-be-nzopyran-5-methoxyl-3,7-diol ; 8-[(E)-4-phenylprop-2-en-1-one]- (2S,3R)-2-(3,5-dihydroxyphenyl)-3,4-dihydro-2H-2-benzopyran-5- methoxyl-3,7-diol; rhamnetin, galangin, isoferulic acid, pinocembrin, chrysin, 5-methoxy-3,7-dihydroxyflavanone, apigenin, isorhamnetin.	HPLC	[24]
**25.**	Shenyang, Liaoning, China<br>Shijiazhuang, Hebei, China<br>Jinan, Shandong, China<br>Zhenzhou, Henan, China	Ethanol extract	caffeic acid, isoferulic acid, 3,4-dimethoxycinnamic acid, pinobanksin 5-methyl ether, pinocembrin, benzyl caffeate, chrysin, galangin.	HPLC-DAD	[25]

Changchun, Jiling, China
Xinxiang, Henan, China
Kunming, Yunnan, China
Hanzhong, Shanxi, China
Qindao, Shandong, China
Nanchang, Jiangxi, China
Chengdou, Sichuan, China
Baoding, Hebei, China
Dalian, Liaoning, China
Datong, Shanxi, China
Jining, Shandong, China
Luoyang, Henan, China
Beijing, China
Guiyang, Guizhou, China
Changge, Henan, China

**26.**	China	Methanol extract	3-O-[(S)-2-methylbu- tyroyl]pinobanksin, 6-cinnamylchrysin, chrysin,galangin, izalpinin, apigenin, techtochrysin, pinostrobin, pinocembrin, isoferulic acid, 3,4- dimethoxycinnamic acid, benzyl ferulate, benzyl caffeate, phenethyl caffeate.	GC, COSY, HMQC, and HMBC spectra	[26]
**27.**	Cyprus, Greeze	Hydro-alcoholic extract	Anthraquinones, emodin, chrysin, galangin, pinocembrin, pinobanksin, pinobanksin-3-O- acetate, isocupressic acid, isopimaric acid, imbricatoloic acid, agathadiol, totarol, 13-epi-torulosal, abietic acid, dehydroabietic acid.	GC/MS	[27]
**28.**	India	Ethanolic extract	Gallic acid, Naringin, Caffeic acid, P-coumaric acid, Ferulic acid, Quercetin, Cinnamic acid, Kaempferol, Chrysin, Phenethyl caffeate, Galangin.	HPLC and NMR	[28]
**29**	Bharatpur, Rajasthan, India	Ethanolic extract	CAPE: caffeic acid phenethyl ester; CA: caffeic acid; GAL: galangin; LUT: luteolin; CUR: curcumin; API: apigenin; PINO: pinocembrin; QUR: quercetin.	HPTLC	[29]

**30.**	Karjat in Raigad District, Maharashtra, India	Ethanolic extract	Nitromethane, 3,3-dimethylpentane 2,5-Dihyrothiophene 3-Hydroxybutanoic acid Ethyl diazoacetate, 2-(Methylenecyclopropyl) ethyl ester acrylic acid 5-Phenoxymethyl-1,3,4-thiadiazol-2-amine Acetate, 3-cyclohexen-1-ol, Boron (methanamine)tris(trifluromethyl), Ethylvinylacetylene, 2-Methyl, 1-penten-3-yne, 2-(3-Bromo-3-buten-1-yl)-1,3-dioxolane (SS)- or (RR)-2,3-hexanediol, 1,2,3,5-Tetrakis-O, arabinofuranose, 1,3,4,5,6-Pentakis-O-, O-methyloxime, d-fructose, 1,2,3,4,5-Pentakis-O- ribitol 3,3-Diphenyl-cyclopropene, Alpha-[p-bromophenyl]-O-amino cinnamic acid Hydroxysteric acid phenacyl ester 4-Ethylformanilide 1,2-Dihydro-4,6-dimethyl-2-oxo, nicotinic acid 1,2,-Benzenedicarboxylic acid 11-Dien-2-one, 4.beta.H,5.alpha.-ermophila-1(10) (RR)-(+), 3,3,4-trimethyl-4-p-tolyl-cyclopentanol.	GC-MS	[30]
**31.**	Mahabaleshwar, Maharashtra, India	alkanes, alkenes, alkyl aldehydes, aryl alkyl	a-Pinene, linalool, undecane, limonene, tidecane, d-fenchone, pentadecane, b-caryophyllene, heptadecana, carvone,	GC and GC-MS	[31]

		ketones, alkanoic acid esters, aromatic acid esters, quinones, chromenes, phenols, steroids, and terpenes extract	geraniol, nonadecane, methyleugenol, heneicosane, eugenol, methyl palmitate, tricosane, tetracosane, (Z)-Ethyl cinnamate, pentacosane, hexacosane, myristic acid, heptacosane, parmitic acid, palmitoleic acid, catechol.		
**32.**	South Sulawesi, Indonesia	stingless bee Tetragonula aff. biroi extracte and ethanol extract	glyasperin A, broussoflavonol F, (2S)-5,7-dihydroxy-4′- methoxy-8-prenylflavanone, (1′S)-2-trans,4-trans-abscisic acid, and (1′S)-2-cis,4-trans-abscisic acid, Sulabiroin A, Sulabiroin B.	Silica gel column chromatograph y, RP-HPLC, NMR, Mass Spectrophotome try	[32]
**33.**	Isfahan, Iran	Ethanol extract	suberosin, tschimgin (bornyl p-hydroxybenzoate), tschimganin (bornyl vanillate), ferutinin (ferutinol p-hydroxybenzoate), and tefernin (ferutinol vanillate).	GC-MS, NMR	[33]
**34.**	Areas of Poplar and Ferula ovina plants in Hamadan (Moradbeig) and Taleghan (Dehdar) districts of Iran, respectively	Ethanol extract	Caffeic acid, Caffeic acid isoprenyl esther (isomer1), Caffeic acid isoprenyl esther (isomer 2), Ferrulic acid, Isoferrulic acid, P-coumaric acid, Quercetin, Quercetin -3 methyl ether, Quercetin -7 methyl ether, Kaempferol,	UPLC-MS	[34]

			Pinobanksin, Pinobanksin 5,7- dimethyl ether, Pinobanksin 3 methyl ether, Pinobanksin -3-O- acetate, Pinobanksin-3-O- proprionate, Pinobanksin—3-O-butyrate, Pinobanksin-3-O- pentanoate, Luteolin -5-methyl ether.		
**35.**	Sabalan Mountains, Ardabil, Iran<br>Alborz Mountains, Mazandaran, Iran	Ethanolic extract	Dimethyl sulfone, Benzeneethanol, Dihydrobenzofuran, 2-Methoxy-4-vinylphenol,2,2-Diethynylbut-2-ene-1,4-diol, -4-Methyl-2-(1-ethylethenyl)-1-cyclopentene-1-carboxaldehyde, 3-Ethyl-8-methyl-2-oxatetracyclo[4.4.0.0(1,4).0(6,8)]decane, Dihydro-.alpha.-terpineol, Rosifoliol, Trifluoroacetic acid, n-heptadecyl ester, 4-Hydroxycinnamic acid, Cinnamic acid, 4-hydroxy-3-methoxy-, 2,5 Dimethoxyterephthalic acid, Methyl 3-(4′-hydroxyphenyl)prop-2-enoate, Aniline, 2,4,6-trimethyl-3-nitro-, 1,4-Dihydrophenanthrene, Pinostrobin chalcone, 3-Methoxy-4,5-methylenedioxybenzaldehyde, Diethylmethylbenzyloxysilane, Caffeic acid, p-Pentyloxynitrobenzene, 5,7- -dihydroxy -dihydroflavone, Galangin flavanone, 1,2-Benzenedicarboxylic acid,	GC-MS	[35]

			Hydrocinnamic acid, Methyl phenylacetate, Benzeneacetic acid, methyl ester, 10-hydroxybenzo[j]fluoranthene, Tectochrysin, Naringenin, Chrysin,5-phenylthiazolidine.		
**36.**	village Lalehzar, Kerman, Iran<br>Hezar- Masjed, Khorasan Razavi, Iran	Water extract	3,7 dihydroxy 5 methoxy flavanones, pinobanksin 3 acetates, pinobanksin 3 butanoate, cinnamic acid, benzoic acid, butanedioic Quercetin, naringenin, cinnamic acid, benzoic acid, butanedioic, propionic acid.	GC-MS	[36]
**37.**	Tehran-Khojir, Iran	Ethanolic extract	2-Hydroxypropanoic acid (lactic acid); 2-Hydroxybutanedioic acid (malic acid); trans-1,4-Butenedioic acid (fumaric acid); Butanedioic acid (succinic acid); Nonanoic acid (pelargonic acid); Decanoic acid (capric acid); Dodecanoic acid (lauric acid); Hexadecanoic acid (palmitic acid); Oleic acid; Octadecanoic acid (stearic acid); 2-Hydroxy acetic acid; 2,3-Dihydroxypropanoic acid (glyceric acid); Tetradecanoic acid (myristic acid); Heptadecanoic acid; 11-Eicosanoic acid; 2,3,4-Trihydroxy butyric acid (tetronic acid); Octanoic acid; Palmitelaidic acid;		[37]

	9,12-Octadecadienoic acid; (Z,Z) a-Linolenic acid; Hydroxy malonic acid; 4-Hydroxybenzoic acid; Benzenepropanoic acid (hydrocinnamic acid); 3-Phenyl; 2-propenoic acid (trans-cinnamic acid); cis-4-Methoxy cinnamic acid; trans-4-Methoxycinnamic acid; cis-3(4-Hydroxyphenyl)-2-propenoic acid (cis-4-coumaric acid); trans-3(4-Hydroxyphenyl)-2-propenoic acid (trans-4-coumaric acid); 3(3,4-Dimethoxyphenyl)-2-propenoic acid (3,4-dimethoxy cinnamic acid; 3(3-Hydroxy-4-methoxyphenyl)-2-propenoic acid (iso ferulic acid); 3(3-Hydroxy-4-hydroxyphenyl)-2-propenoic acid (ferulic acid); 3(3,4-Dihydroxyphenyl)-2-propenoic acid (caffeic acid); 3-Methoxy; 4-hydroxybenzoic acid (isovanillic acid); 3(3-Methoxy, 4-hydroxyphenyl)-2-propenoic acid (isomer 1); Delta-9-tetra-hydrocannabinol acid; 3,4-Dihydroxybenzoic acid (protocatechuic acid); 3(3-Methoxy, 4-hydroxyphenyl)-2-propenoic acid (isomer 2); Hexadecanoic acid; ethyl ester (ethyl palmitate); 1,2-Benzenedicarboxylic acid; diethyl ester	

	(diethyl phthalate); Benzyl-trans-4 coumarate 1-Phenylethyl trans caffeate; Cinnamyl caffeate; 3-Methyl-3-butenyl-trans-ferulate; 3-Methyl-2-butenyl-trans-ferulate; 3-Methyl-3-butenyl-trans-iso ferulate; 3-Methyl-3-butenyl-trans-caffeate; 2-Methyl-2-butenyl-trans-caffeate; 3-Methyl-2-butenyl-trans-caffeate, 2-Methyl-2-butenyl-trans-4-coumarate; 3-Methyl-2-butenyl-trans-4-coumarate; Phenylethyl trans-4-coumarate; Linoleic acid ethyl ether (ethyl linoleate); 2-Propenoic acid; 3-phenyl-, ethyl ester (ethyl cinnamate); 3(3,4-Dimethoxyphenyl)-2-propenoic acid; methyl ester 3(4-Methoxy, 3-hydroxyphenyl)-2-propenoic acid; methyl ester 3(3-Methoxy, 4-hydroxyphenyl)-2-propenoic acid; methyl ester 3(3,4-Dihydroxyphenyl)-2-propenoic acid; ethyl ester (ethyl oleate) Stearic acid; ethyl ester (ethyl stearate); Flavonoids 5-Hydroxy-7-methoxy flavanone (pinostrobin); 5,7-Dihydroxy flavanone (pinocembrin); 2′,6′-Dihydroxy-4′-methoxydihydro chalcone; 5,7-Dihyroxy-3-acetyloxyflavanone (pinobanksin-3-	

acetate)(Isomer1); 5,7-Dihyroxy-3-acetyloxyflavanone (pinobanksin-3-acetate)(Isomer2); 5,7-Dihydroxy flavone (chrysin), 3,5,7-Trihydroxy flavanone (piobanksin), 3,5,7; Trihydroxy flavone (galangin); 2′,4′,6′-Trihydroxy chalcone (pinocembrin chalcone); 3 3,5,7,40-Tetrahydroxy flavone (kaempferol); 3,5,7,40-Tetrahydroxy flavone (kaempferol); 5,7-Dihydroxy flavone (chrysin); 5,7-Dihyroxy-3-propanoyloxyflavanone (pinobanksin-3-propanoate); 3,5,7-Trihydroxy flavone (galangin); 5,7-Dihyroxy-3-(iso)butanoyloxyflavanone (pinobanksin-3-isobutanoate); 5,7-Dihyroxy-3-(iso)pentanoyloxyflavanone (pinobankin-3-isopentanoate); 5,7,4′-Trihydroxy flavanone (naringenin); 3,5,7,30,40-Pentahydroxy flavone (quercetin); Quercetin methyl ether; 3,5,7,30,40-pentahydroxy flavone (quercetin); Myo inositol Sucrose; D-Fructose (isomer 1); D-Fructose (isomer 2); Sorbose; D-Glucitol; D-Glucose; a-D-Xylopyranose L-Gluconic acid; D-Galactose; Aliphatic hydrocarbons Heptadecane; 2-

			Nonadecanone Eicosane; Aldehydes 4-Hydroxybenzaldehyde; 3-Methoxy; 4-hydroxybenzaldehyde (vanillin); 3,4-Dihydroxybenzaldehyde; Sesquiterpenes trans; trans-farnesol a-Cedrol; 4-bH,5a-Eremophil-1 (10)-ene; ga-Cadinene; Isopimaric acid; 1,2,3-Propanetriol (glycerol); Phosphate; 1,4-Benzenediol (hydroquinone); Citric acid; 1-(5-ethenyltetrahydro-5-methyl-2-furanyl)- 1-methylethanol; trans 1-Phenyl-1-propen-3-ol 20-Hydroxyacetophenone		
**38.**	Urmia, Iran	Ethanol extract	2-Hydroxy-5-methylbenzaldehyde; 5-Hydroxy-7-methoxy flavanone (pinostrobin); 5,7,40-Trihydroxy flavanone (naringenin); 5,7-Dihydroxy flavone (chrysin); Dihydrochrysin; 3(3,4-Dihydroxyphenyl)-2-propenoic acid (caffeic acid); Cis-lanceol Caryophyllene oxide; Eudesmol; 6-Hydroxy-1-oxogermacr-4,10(15),11(13)-trien-12,8-olide; 3,12-Oleandione; 1-Heptatriacotanol; 1,5,5-Trimethyl-6-methylene-cyclohexene; Aromatic hydrocarbons; 2-Amino-1-(3-hydroxy-4-methoxyphenyl) ethanone; 1,3,8-	GC-MS	[38]

			trihydroxy-6-methylanthracene-9,10-dione		
**39.**	Baghdad, Iraq<br>Dahuk, Iraq<br>Mosul, Iraq<br>Salah ad-Din, Iraq	Methanol extracts	caffeic acid; coumaric acid; vanilin; methyl caffeate; ferulic acid; ferulic acid methyl ester; luteolin; quercetin; sakuranetin; methyl quercetin; cinnamic acid; tectochrysin; apigenin; naringenin; pinobanksin; kaempferol; kaempferide; bic-methylated quercetin; acacetin; hesperetin; prenyl caffeate; benzyl caffeate; chrysin; pinocembrin; galangin; caffeic acid phenethyl ester; pinobanksin-3-acetate; isopentyl caffeate; isoprenyl coumarate; isoprenyl ferulate; pinobanksin-3-propionate; clerodane diterpenoid I ; clerodane diterpenoid II; pinostrobin; clerodane diterpenoid dihydro; clerodane diterpenoid dehydrated; palmitic acid.	HPLC–ESI/MS	[39]
**40.**	Okinawa, Japan		Propolin A, Propolin B, Prokinawan, propolin E, Nymphaeol-B, iso-nymphaeol B, nymphaeol-A, 3’geranyl-narigenin, nymphaeol-C.	HPLC	[40]

**41.**	Okinawa, Japan	Ethanol extracts	nymphaeol-B; isonymphaeol-B; nymphaeol-A; 3′-geranyl-naringenin; nymphaeol-C.	HPLC	[41]
**42.**	Amman, Jordan	Methanolic extracts	cinnamyl cinnamate; tetracosanoic acid; tectochrysin; pinocembrin; pinobanksin-3-O-acetate; 3-methylethergalangin; chrysin; galangin; genkwanin; alpinone; naringenin; apigenin; cryptomeridiol; agathadiol; 24-(Z)-3-oxolanosta-1,7,24-trien-26-oic acid.	H-NMR, C-NMR, TLC	[42]
**43.**	Jarash, Jordan	Methanol, hexane, and ethyl acetate extracts	2H-2,4a-Ethanonaphthalene ,1,3,4,5,6,7-hexahydro-2,5,5- trimethyl; 2-methyl-, 2-phenylethyl ester; Phenethylamine; p-methoxy-α-methyl; Guaiol; 3-Methyl-4-isopropylphenol; Naphthalene; 1,2,4a,5,6,8a-hexahydro-4,7-dimethyl-1-(1-methy- lethyl); Naphthalene; 1,2,3,4,4a,5,6,8a-octahydro-4a,8-dimethyl-2-(1- methylethenyl); 2-Propenoic acid, 3-phenyl-, ethyl ester, (E); Longipinene; 2-Naphthalenemethanol; 1,2,3,4,4a,5,6,7-octahydro- α,α,4a,8-tetramethyl-(2R-cis); 2-Naphthalenemethanol; decahydro-alpha; 4a-trimethyl-8- methylene-; [2R-(2.alpha.,4a.alpha.,8a.beta.)]; 4,8,13-	GC-MS	[43]

			Cyclotetradecatriene-1,3-diol; 1,5,9-trimethyl-12-(1-meth- ylethyl).		
**44.**	South Lebanon	Ethanolic extract	Caffeic acid, Rutin, Ferulic acid, Quercetin, Daidzein, Genistein, Apigenin, Kaempferol, Chrysin, Pinocembrin, Galangin.	LC-MS/MS	[44]
**45.**	Pahang, Malaysia	Ethanol and ethyl acetate extracts	Quercetin, Luteolin, Kaempferol, Apigenin, Pinocembrin.	RP-HPLC	[45]
**46.**	Bharu, Kelantan, Malaysia	Ethanol extract	2,5-furandione or succinic anhydride; penylacetaldehyde; bezeneethanol; benzoic acid; 2-courmaranone; benzeneacetic acid; decanoic acid; isocaryophyllene; bela selinene; alpha copaene; alloaromadendrebe; alpha selinene; gamma cadinene; delta cadinene; lauric acid; spathulenol; caryophyllene oxide; hexadecanoic acid, methyl ester; hexadecanoic acid ethyl ester; 5-octadecene; 8-octadecenoic acid methyl ester; 9-octadecenoic acid (z) ethyl ester; 9-octadecen-1-09z; phenol,3-pentadecyl; hydroxymethyloethyl acid, bis (2-ethylhexyl) ester; 5-heptylresorcinol; 1,3-benzenediol,5-		[46]

			pentadecyl; lanosta-8,24-dien-3-ol (3 beta); 9,19-cyclolanostan-3-ol,24,methylene (3beta); 9, 19- Cyclo- 9 beta- lanostane- 3 beta 25 diol; 9, 19- Cyclolanostan- 3- ol, 24- methylene (3 beta)		
**47.**	Ywar Taw village, Shan, Myanmar	Methanolic extract	(22Z,24E)-3-oxocycloart-22,24-dien-26-oic acid, (24E)-3-oxo-27,28-dihydroxycycloart-24-en-26-oic acid, 28-hydroxymangiferonic acid, 27-hydroxymangiferonic acid, (24E)-3-oxo-23-hydroxycycloart-24-en-26-oic acid, (24E)-3 -hydroxycycloart-24-en-26-al, isomangiferolic acid, mangiferolic acid, (24E)-3R,27-dihydroxycycloart-24-en-26-oic acid, (24E)-3 ,27-dihydroxycycloart-24-en-26-oic acid, (24E)-3R,22-dihydroxycycloart-24-en-26-oic acid, (24E)-3 ,23-dihydroxycycloart-24-en-26-oic acid, (2S)-5,7-dihydroxy-4′-methoxy-8,3-diprenylflavanone, (2S)-5,7,4′-trihydroxy-8,3′-diprenylflavanone, (2S)-5,7-dihydroxy-4′-methoxy-8-prenylflavanone, (2S)-5,7,4′-trihydroxy-8-prenylflavanone	NMR spectra, silica gel column chromatography, FABMS AND HRFABMS measurements, MPLC, silica gel TLC	[47]

**48.**	Shan, Myanmar	Methanolic extract	isomangiferolic acid; mangiferolic acid; 3a ,27-dihydroxycycloart-24E-en-26-oic acid; 3b ,27-dihydroxycycloart-24E-en-26-oic acid; 3a ,22-dihydroxycycloart-24E-en-26-oic acid; 3b ,23-dihydroxycycloart-24Een-26-oic acid; (24)-3b -hydroxycycloart-24E-en-26-al; mangiferonic acid; 27-hydroxylmangiferonic acid; 3-oxo-23-hydroxycycloart-24E-en-26-oic acid; 28-hydroxymangiferonic acid; 3-oxo-27,28-dihydroxycycloart-24E-en-26-oic acid; 3-oxo-cycloart-22Z,24Edien-26-oic acid; 5,7-dihydroxy-4 -methoxy-8,3 -diprenylflavanone; (2S)-5,7,4 -trihydroxy-8,3 -diprenylflavanone; (2S)-5,7-dihydroxy-4 -methoxy-8-prenylflavanone; (2S)-5,7,4 -trihydroxy-8-prenylflavanone.	Mass Spectrophotometry	[48]
**49.**	Chitwan, Nepal	Methanolic extract	(S)-4-methoxydalbergione; cearoin; hydroxy-6,7-dimethoxydalbergiquinol; obtusaquinol; 2′,4,4′-trihydroxychalcone; medicarpin; (+)-vesticarpan; 4-hydroxymedicarpin.	H NMR, COSY, MNQC, HMBC, LC UV, LC-MS	[49]

**50.**	Chiwan, Nepal	Methanol and water extract	Chrysin; 4-methoxydalbergion; cearoin; 3′,4′-dihydroxy-4-methoxydalbergione; isoliquiritigenin; plathymenin; 7-hydroxyflavanone; (+)-medicarpin; 4-methoxydalbergion.	HPLC	[50]
**51.**	Chiwan, Nepal	Methanol and water extract	Isoliquiritigenin; 6-hydroxy-3-methoxy-6-(3-phenyl-2-propenyl)-2- cyclehexane-1-one; plathymenin; 7-hydroxyflavanone; chrysin; 3′,4′-dihydroxy-4-methoxydalbergione; 4-methoxydalbergion; cearoin; medicarpin; formononetin.	HPLC	[51]
**52.**	Korak village, Nepal	Ethanolic extracts	Plathymenin; Butin; 6,7-dihydroxyflavanon; 4′-Methoxy-2′,3, 7-trihydroxyisoflavanone; 2′-Hydroxyformononetin; Odoratin; Liquiritigenin; 2-(1-Phenylprop-2-enyl)benzene-1,4-diol; 2′,7-dihydroxy-5-methoxyisoflawan; Chalcone; Butein; Cearoin; Dalbergin; 7-Hydroxyflavanone; Isoliquiritigenin; Obtusaquinol; Medicarpin; Pinocembrin; 4-Metoxydalbergione.	HPLC-DAD-MS/MS analyses	[52]

**53.**	Chitwan, Nepal	Methanolic extract	Odoratin; (+)-Medicarpin; (+)-Vesticarpan; Plathymenin; (2S)-7-Methoxyflavanone; (2S)-7-Hydroxyflavanone; S-4-Methoxydalbergiquinol, (4S,6S)-4-Hydroxy-3-methoxy-6-(1-phenyl-2-propenyl)-2-cyclohexene-1-one; Cearoin; (1R,2S,4S,5S)-2-methoxy-5-[(1R)-1-phenyl-2-propenyl]-1,4-cyclohexanediol	PTLC, ODS HPLC, NMR	[53]
**54.**	Omani		8-prenyl-5,7-dihydroxy-3′-(3-hydroxy-3-methylbutyl)- 4′-methoxyflavanone, fisetinidol (-)-MOLLISACACIDIN, (+)-MOLLISACACIDIN, 7-O-methyl-8-prenylnaringenin, cardanol, 3′,8- diprenylnaringenin	GC-MS, NMR	[54]
**55.**	Phillipines	Ethanolic extract	artepillin C, cafeic acid phenethyl ester (CAPE), squalene	LC-MS/MS	[55]
**56.**	San Roque, Sorsogon, Philippines	Dichloromethane extracts	glyasperin A, propolin A, propolin E, propolin H, lupeol, α-amyrin and β-amyrin, urs12-en-3-one, olean-12-en-3-one and lup-12-en-3-one	NMR	[56]

**57.**	Southern Arabian peninsula, Asir, Saudi Arabia	Ethanol extract	propsiadin ((ent)-2-oxo-kaur-16-en-6,18-diol), 3,4-dihydro-2-(3,4-dihydroxyphenyl)-2H-chromene-3,7-diol, psiadiarabin, diterpene psiadin	LC–MS, 1D 2D NMR	[57]
**58.**	Riyadh and Al-Bahah, Saudi Arabia	Hexane extract	α-amyrones, β-amyrones, amyrins, amyryl acetates, tridecane, tetradecane, penthadecane, hexadecane, heptadecane, octadecane, nonadecane, eicosane, heneicosane, docosane, tricosane, tetracosane, penthacosane, hexacosane, heptacosane, octacosane, nonacosane, triacortane, hentriacortane, dotriacortane, tritriacortane, tetratriacortane, penthatriacortane, hexatriacortane, heptatriacortane, octatriacortane, nonatriacortane, tridecane, tetradecane, pentadecane, hexadecane, heptadecane, octadecane, nonadecane, eicosene, heneicosene, docosene, tricosene, tetracosene, pentacosene, hexacosene, haptacosene, octacosene, nonacosene, triacontene, hentriacontene, dotriacontene, tritiacontene, tetratriacontene, pentatriacontene, tetradecanal, eicosanal, docosanal, tetracosanal, hexacosanal,	GC-MS, PCA using SPSS	[58]

			octacosanal,triacontanal, methyl dodecannoate, methyl tetrdecanoate, methyl hexadecanoate, methyl octadecanoate, methyl octadecenoate, methyl eicosanoate, methyl docosanoate, methyl tetracosanoate, methyl hexacosanoate, tetracosanyl hexadecanoate, hexacosanyl hexadecanoate, octacosanyl hexadecanoate		
**59.**	Al-Baha, Saudi Arabia	Methanol Extract	Totarol, Ferruginol, Methyl octadecenoate, hinokione, sugiol, hinokiol, hydroginkgol, squalene, Urs-9(11), 12 dien-3-one, dammaradienol taraxasterol, Lup-20(29)en-3-one, α-amyrin, lanosterol, β-amyrone, β-amyrin, cycloartenol, moretanol, lupeol, olean-13(18)-en-3-one, hentriacontene, tritriacontene, oleana-9(11)-dien-33-ylacetate, β-amyryl acetate, Cycloartenyl acetate, Lanostenyl acetate, α-amyryl acetate, β-lupenyl acetate, α-lupenyl acetate, Di-octyl-phthalate	GC-MS	[59]
**60.**	Al-Baha, Saudi Arabia	Ethyl acetate extract	(12E)-communic acid, (12Z)-communic acid, sandaracopimaric acid, (+)-	HPLC, APCI-MS/MS, NMR	[60]

			ferruginol, (+)-totarol, linoleic acid, oleic, palmitic acid, stearic acid, 3β-Acetoxy-19(29)-taraxasten-20a-ol, 15-O-oleoyl isocupressic acid, 15-O-palmitoyl isocupressic acid, cyclartenol, 24-methylene-cycloartenol, β-Amyrin-3β-O-actetate, α-Amyrin-3β-O-actetate, taraxasterol-3β-O-actetate, lupeol-3β-O-actetate		
**61.**	Behera and UAE, Saudi Arabia	Ethanolic extracts	Malic acid, Succinic acid, Palmitic acid, Oleic acid, Octadecenoic acid, Octadecadienoic acid, Stearic acid, Tetracosanaoic acid, 2-Amino-3-methoxybenzoic acid, Benzenepropionic acid, Cinnamic acid, 4-Hydroxycinnamic acid, 4-Methoxycinnamic acid, Caffeic acid, Ferulic acid, Glycerol, 2-Naphthalenemethanol derivatives, 2-Methoxy-4-vinylphenol, Butyraldehyde, 4-Methoxyhydrocinnamate, 3-Methyl-3-butenyl isoferulate, Ethyl palmitate, 5,7-Dihydroxyflavone, 5,7-Dihydroxydihydroflavone, 1,7-Dihydroxy-3-methoxy-6-methylanthraquinone , 1,3,8-Trihydroxy-6-methylanthraquinone, 1-	GC-MS	[61]

			(Dihydroxyphenyl)-3-phenylpropenone, 5-Hydroxy-7-methoxy-2-phenyl- 4H-1-benzopyran-4-one, Tricosane, Octacosane, Docosane, Pentatricosane		
**62.**	Istanbul, Turkey	Ethanol extracts	Kaempferol, Fumaric acid, Pyrogallol, p-OH benzoic acid, Vanillin, p-coumaric acid, Caffeic acid, t-ferulic acid, Quercetin, Ellagic acid, Isorhamnetin, Quercetagetin-3,6-dimethyl ether, Chlorogenic Acid, Rosmarinic acid, Kaempferol-3-O-Rutinoside, Rutin Hydrate, Gallic acid, Salvigenin, Penduletin	LC-MS/MS	[62]
			Galangin, Chrysin, Pinobanksin, Pinocembrin, Pinostrobin, Apigenin, Luteolin, t-cinnamic acid, Quercetin dihydrate	HPLC	
**63.**	Turkey	Ethanolic extract	3-O-methylquercetin, chrysin, caffeic acid, caffeic acid phenethyl ester/CAPE, galangin, pinocembrin	HPTLC and HPLC Analysis	[63]
**64.**	Mamara, Turkey<br>Aegean, Turkey	Ethanol extract	Benzaldehyde, Isovanillin, Vanillin, 4-Methylene-5-hexenal, Cinnamaldehyde, Oleic acid ethyl ester, 6-Octadecenoic acid	GC-MS analysis	[64]

			methyl ester, Hexadecanoic acid ethyl ester, Octadecanoic acid ethyl ester, Oleic acid, Heptadecanoic acid ethyl ester, E-11-Hexadecenoic acid ethyl ester, Linoleic acid ethyl ester, Decanoic acid ethyl ester, 5-Phenyl-4-pentenoic acid, 5,7-Dihydroxy-4'-methoxyflavone, Pinostrobin chalcone,5,7-Dihydroxy flavanone, 5-Hydroxy-7-methoxyflavone, 4,5-Dihydroxy-7-methoxy flavanone, 5,7-Dihydroxy flavone, Benzyl cinnamate, β-Myrcene, trans-Caryophyllene, α-Selinene, γ-Selinene, 1R-α-Pinene, γ-Muurolene, Aromadendrene, β-Phellandrene, Junipene, Totarol		
**65.**	Aegean, Black Sea, Central Anatolia, Marmara, Mediterranean Regions of Turkey	Hydroalcoholic extracts	pinocembrin, CAPE, 3-O-methylquercetin, caffeic acid, ferulic acid, quercetin, galangin	HPTLC, MPLC, NMR	[65]
**66.**	Anatolia, Hatay, Turkey<br>Bursa, Turkey	Ethyl alcohol extract	3-Methyl-3-buten-1-ol, Phenylethyl alcohol, (E)-11-Hexadecen-1-ol, 2-Propen-1-ol, 2-Naphthalene-methanol, 13-Tetradecy-11-yn-1-ol, Olean-12-en-3-ol, Benzenemethanol, 5-Phenyl-4-pentenoic	GC-MS	[66]

acid, Benzoic acid, Benzenepropanoic acid, 3-Phenyl-2-propenoic acid, Decanoic acid, 9-Octadecenoic acid, Octadecanoic acid, Benzene acetic acid, 4-hydroxy-3-methoxymethyl ester, Octadecanoic acid-methyl ester, 1,2-Benzenedicarboxylic acid, bis(8-methyl nonyl) ester, 1,2-Benzenedicarboxylic acid, bis(8-methyl propyl) ester, 1,2-Benzenedicarboxylic acid, butyl 8-methylonyl ester, Benzyl cinnamate, 1,2-Benzenedicarboxylic acid, diisodecyl ester, Benzaldehyde, Tetradecanoic acid, Heptadecanoic acid, n-Hexadecanoic acid, Tetradecanoic acid, ethyl ester, Hexadecanoic acid, ethyl ester, 15-methyl-ethyl ester, 4H-1-Benzopyran-4-one, 5-hydroxy-7-methoxy-2-phenyl, 4H-1-Benzopyran-4-one, 2,3-dihydro-5, 7-dihydroxy-2-phenyl, 4H-1-Benzopyran-4-one, 3,5,7-trihydroxy-2-phenyl, Chrysin, 5,7-Dihydroxy-6-methoxy-3(4′-methoxyphenyl), 5-Hydroxy-6,7-dimethoxy-3(4′-methoxyphenyl), Cyclotetradecane, Heptadecane, 1-Heptadecane, 1-Nonadecene, 9-Tricosene, Delta-cadinene, Bicyclo(4.4.0) dec-1-ene,

			6(Z) 9(E)-Heptadecane, 2-Propen-1-one, Ethyl oleate, Cinnamyl cinnamate, 3-Hydroxy-4-methoxycinnamic acid, 2(5H)-Furanone, 5,5-diphenyl, 2-Phenyl-2-tipyl-acenapthenone, 1-(2-Vinyl phenyl)ethanone, Totarolone, Hinokione, 2-Heptadecanoate		
**67.**	Yozgat, Turkey<br>Izmir, Turkey<br>Kayseri, Turkey<br>Adana, Turkey<br>Erzurum, Turkey<br>Artvin, Turkey<br>Ankara-Kazan, Turkey<br>Mugla-Marmaris, Turkey<br>Turkey	Hydro-alcoholic extracts	Quercetin, rutin, chrysin, pinocembrin, pinostrobin, chalcone, pinobanksin, gallocatechin, epigallocatechin, myricetin, α-eusdemol, β-eusdemol, isopimaric acid, abietic acid, p-coumaric, feruli, benzoic acid, caffeic acid, cinnamid acid, angelicin, bergapten, bergaptol, columbianetin, decursin, isogeijerin, jatamansin, lomatin, methoxsalen, oroselone, osthole, oxypeucedanin, prangenin, proralen, seselin	GC-MS, HPLC, (UHPLC-LTQ/orbitrap/MS/MS)	[27]
**68.**	Erzurum, Turkey	Water extract	Curcumin, Caffeic acid, Ferulic acid, Syringic acid, Ellagic acid, Quercetin, a-Tocopherol, Catechol, Pyragallol, p-Hydroxy benzoic acid, Vanillin, p-Coumaric acid, Gallic acid, Ascorbic acid	HPLC, LC-MS	[67]

**69.**	Turkey	Ethanol extract	Apigenin, Quercetin, Chrysin, Caffeic acid, Naringenin, CAPE, Pinobanksin, Galangin,, Pinocembrin	HPTLC	[68]
**70.**	Izmir, Turkey	Acetone extract	acenaphthylene, acenaphthene, phenanthrene, anthracene, fluoranthene, pyrene, benzo(b)fluoranthene, benzo(a)pyrene, acenaphthylene, acenaphthene, fluorene, fluoranthene, pyrene, chrysene, benzo(k)fluoranthene, dibenzo(a,h)anthracene, indeno(1,2,3-cd) pyrene, benzo(ghi)perylene	GC	[69]
**71.**	Ankara-Kazan, Turkey Mug˘la-Marmaris, Turkey	ethanolic extract	1H-3A,7-Methanoazulene, 2,3,4,7,8,8A-hexahydro-3,6,8,8-tetramethyl-[3R-(3α,3a,7, 8aα)]; 8-H-cedran-8-ol; 1,4-anhydroglucitol; 1-naphthalenemethanol, decahydro-1,10-dimethyl-6-methenyl-5-(5-hydroxy-3- pentene); 3-α,5--pregnan-20-one; androstan-1,17- dimethyl-17-hydroxy-3-one; thunbergol; docosa-8,14-diyn-cis-1,22-diol; 4-H,5α-eremophi1Đ1(10)-ene; 1-(5-ethenyltetrahydro-5-methyl-2-furanyl)-1-methylethanol; 4-H,5α-eremophi1Đ1(10)-ene; 1-(5-ethenyltetrahydro-5-methyl-2-furanyl)-1-methylethanol	GC-MS	[70]

**72.**	Balikesir, Turkey<br>Istanbul, Turkey	ethyl alcohol	3-Methyl-2-butenol, Ethyl decanoate, Ethyl benzoate, Diethyl succinate, Ethyl decanoate, Calamenene, Ethyl-3-phenyl propionate, Phenylethyl alcohol, (E)-Ethyl cinnamate, y-Eudesmol, α-Eudesmol, ß-Eudesmol, Ethyl hexadecanoate, Decanoic acid, Ethyl oleate	GC-MS	[71]
**73.**	Chilgok, South Korean<br>Cheongju, South Korean<br>Geochang, South Korean<br>Muju, South Korean<br>Pocheon, South Korean<br>Sangju, South Korean	ethanol extract	caffeic acid; p-coumaric acid; 3,4-dimethoxycinnamic acid; pinobanksin 5-methyl ether; apigenin; kaempferol; pinobanksin; cinnamylideneacetic acid; chrysin; pinocembrin; galangin; pinobanksin 3-acetate; phenethyl caffeate; cinnamyl caffeate; tectochrysin.	HPLC with PDA and MS detection	[72]
**74.**	Yangpyeong, Gyeonggi-do, Korean	ethanolic extract	caffeic acid phenethyl ester, caffeic acid benzyl ester, caffeic acid ethyl ester, ferulic acid benzyl ester, ferulic acid 30,30-dimethylallyl ester, 3,4-dimethoxycaffeic acid cinnamyl ester, coumaric acid cinnamyl ester, coumaric acid benzyl ester, cinnamic acid	NMR and ESI-MS	[73]

			phenethyl ester , and cinnamic acid cinnamyl ester .		
**75.**	Jeju Island, South Korean	EtOH extract	(±)-(E)-4′-methoxy-4,2′-dihydroxy-3′-(2′′,3′′-dihydroxy-3′′-methylbutyl)-chalcone; (E,E,E)-4,2′,4′-trihydroxy-3′-(7′′-hydroxy-3′′,7′′-dimethyloct-2′′,5′′-dienyl)-chalcone; (±)-(E,E)-4,2′,4′-trihydroxy-3′-(5′′-hydroxy-3′′,7′′-dimethyloct-2′′,6′′-dienyl)-chalcone; (±)-(E)-4′-methoxy-4,3′,4′′-trihydroxy-2′′,2′′-dimethyldihydropyrano-(2′,3′)-chalcone; (±)-(E)-4′-methoxy-4,3′′-dihydroxy-2′′-(1′′ -hydroxyisopropyl)-dihydrofurano-(2′,3′)-chalcone; (–)-(E)-4,4′-dihydroxy-2′′-(1′′-hydroxy-1′′ ,5′′ ′-dimethylhex-4′′-enyl)-dihydro furano-(2′,3′)-chalcone; (+)-(E)-4,2′-dihydroxy-2′′-methyl-2′′-(3′′,4′′-dihydroxy-4′′ -methylpentanyl)-2H-pyrano-(3′,4′)-chalcone; and (–)-(E)-4,2′-dihydroxy-2′′-methyl-2′′-(3′′,4′′-dihydroxy-4′′-methylpentanyl)-2H-pyrano-(3′,4′)-chalcone; 4-hydroxyderricin, (±)-TB5, (±)-xanthoangelol B, xanthoangelol xanthoangelol F, (±)-bavachromanol , (±)-xanthoangelol H , (+)-TB2 , (±)-lespeol,(+)-TB1, (SS)-(+)-laserpitin , (SS)-(–)-	Yanaco MP-500 micromelting point apparatus, Horiba SEPA-200 polarimeter, Jasco DIP-1000 digital polarimeter, UV/VIS spectrophotometer, FABMS spectra, ESIMS spectra,1D and 2D NMR spectra, Silica gel Column Chromatography (CC), RP-HPLC	[74]

			isolaserpitin , (S)-(–)-selinidin, (RR)-(+)-khellactone, (RR)-(+)- 30-senecioylkhellactone, (20S,30R)-(+)-vaginidiol, (20S,30R)-(+)-daucoidin A , (S)-(–)-oxypeucedanin hydrate and (S)-(+)-marmesin		
**76.**	South Korea	Methanol extract	cinnamic acid, benzoic acid and their esters, substituted phenolic acids and esters, flavonoid glycones, bee wax, and caffeic acid phenethyl ester (CAPE)	HPLC, MS/MS	[75]
**77.**	Jeju Island, South Korean	Methanol extract	(SS)-(þ)-laserpitin; (SS)-( )-isolaserpitin; (S)-( )-selidin; 4-hydroxyderricin; xanthoangelol; xanthoangelol F.	HPLC	[76]
**78.**	Taiping, Taichung, Taiwan<br>Wufeng,Taichung, Taiwan Changhua, Taiwan<br>Chiayi, Taiwan<br>Tainan, Taiwan<br>Ping Tung, Taiwan	Ethyl alcohol	propolin D ; propolin F; propolin C; propolin G	TLC, HPLC, GC-MS	[77]
**79.**	Taiwan	Ethanol extract	Propolin C, Propolin D, Propolin F, and Propolin G	HPLC	[78]

**80.**	Chiangmai, Thailand	MeOH extract	(7″S)-8-[1-(4′-hydroxy-3′-methoxyphenyl)prop-2-en-1-yl]-(2S)-pinocembrin and (E)-cinnamyl-(E)-cinnamylidenate.	NMR, Column chromatography, TLC	[79]
**81.**	Nan province, Thailand	ethanol and water extracts	seven new p-coumaric acid derivatives along with seventeen known compounds, including four flavonoids, one prenylated phenolic acid, four diterpenoic acids, one lignan, two p-coumaric acid esters and five cinnamic acid derivatives, were isolated from the relatively polar ethyl acetate soluble fraction of a 75% (v/v) EEP of Brazilian propolis	NMR, 1D-TLC	[80]
**82.**	Trat Province, Thailand<br>Chiang Mai, Thailand	Ethanol extract	α-mangostin**,** mangostanin, 8-deoxygartanin , gartanin, γ-mangostin, dipterocarpol, xanthone garcinone B, methylpinoresinol, 3-O-acetyl ursolic acid, dipterocarpol, ocotillone I, ocotillone II, ursolic, oleanolic aldehydes, cabralealactones, 2,3-dihydroxyoleanadien-28-oic acid, 2,3-dihydroxyursadien-28-oic acid,	GC-MS, TLC, NMR	[81]

**83.**	Pua, Nan, Thailand	hexane and dichloromethane extracts	Cardanol, Cardol	TLC, NMR, MS	[82]
**84.**	Makham, Chanthaburi, Thailand	Ethanol extract	αMangostin, γ- Mangostin	HPTLC	[83]
**85.**	Uzbekistan	Methanol Extract	Caffeic acid; p-coumaric acid; 3,4-dimethoxycinnamic acid; quercetin; pinobanksin 5-methyl ether; apigenin; kaempferol; pinobanksin; cinnamylideneacetic acid; chrysin; pinocembrin; galangin; pinobanksin 3-acetate; phenethyl caffeate; cinnamyl caffeate; tectochrysin; artepillin C.	HPLC analysis with photo-diode array (PDA) and mass spectrometric (MS) detection, and quantitatively analyzed	[84]
**86.**	Binhdinh, Vietnam	Ethanol extract	dihydroxy-5-methoxy-8-methylflavane 1; (3R)-7,4′-dihydroxyhomoisoflavane; (3S)-7,4′-dihydroxy-5-methoxyhomoisoflavane; 10,11-dihydroxydracaenone C, 3-geranyloxy-1,7-dihydroxyxanthone (cochinchinone G), 7-geranyloxy-1,3-dihydroxyxanthone, 2,6,8-trihydroxy-5-geranyl-7-prenylxanthone (cochinchinone A), α-mangostin, garcinone B, cycloartenone, lupeol, resorcinols	NMR, VLC, Column chromatography Low pressure liquid chromatography (LPLC), Preparative thin-layer chromatography (prep. TLC), GC-MS	[85]

**87.**	Vietnam	Ethanol extract	Tetragocarbon A; Tetragocarbon B; Mammein cinnnamoyl ester; 5,7-Dihydroxy-6-(4-cinnamoyl-3-methyl-1-oxobutyl)-4-phenylcoumarin; 4-(4'-Hydroxy-3'-methoxyphenyl)-3,5,7-trihydroxycoumarin; Sulawesin A; and Sulawesin B	LC-MS, GC-MS, NMR, MS	[86]
**88.**	Ben Tre Province, Vietnam	Ethanol extract	27-acetoxymangiferonic acid, (5R,8S,9S,10R,13R,14S,17R,20R)-27-methoxycarbonyloxymangiferonic acid, 27-acetoxymangiferolic acid, 23-hydroxyisomangiferolic acid A, 23-hydroxyisomangiferolic acid B, cycloartenone, mangiferonic acid,23-hydroxymangiferonic acid, 27-hydroxymangiferonic acid, mangiferolic acid, 23-hydroxymangiferolic acid, 27-hydroxymangiferolic acid, 27-hydroxyisomangiferolic acid, (24E)-3β-hydroxycycloart-24-en-26-al, (23E)-27-nor-3β-hydroxycycloart-23-en-25-one, and lanosterol	NMR	[87]
**89.**	Tarim, Yemen<br>Wadi Adem, Yemen	Dichloromethane and methanol	(β-Amyrone, α-Amyrone, α-Amyrin; β-Amyrin; α-Amyryl acetate; β-Amyryl	GC-MS	[88]

Seiyun, Yemen<br>Seiyun Shahoh, Yemen<br>Amran, Yemen<br>Thebi-Tarim, Yemen	acetate, α-Amyryl pentanoate, β- Amyryl pentanoate, Amyryl hexanoate, α-Amyryl hex-5-enoate, β-Amyryl hex-5-anoate, Moretenol, Dammaradienol, 3β-Lupenyl acetate, 3α-Lupenyl acetate, Dammaradienyl pentanoate, Dammaradienyl hex-5-enoate, Pentacosene, Hexacosene, Heptacosene, Octacosene, Nonacosene,Triacontene, Hentriacontene, Tritriacontene, Tetratriacontene, Pentatriacontene, Eicosane, Heneicosane, Docosane, Tricosane, Pentacosane; Hexacosane, Heptacosane; Octacosane, Nonacosane; Triacontane and Hentriacontane, Hexadecanoic acid, Octadecenoic acid, Octadecanoic acid, Eisanoic acid, Tetracosanoic acid, Octadecyl hexadecanoate, Eicosyl hexadecanoate, Dodecasanyl hexadecanoate, Tetracosanyl hexadecanoate, Octacosanyl hexadecanoate, Octadecanol, Eicosanol, Docosanol, Tetracosanol, Hexacosanol, Octacosanol, Triacontanol, Dotriacontanol, Methyl dodecanoate, Methyl tetradecanoate, Methyl hexadecanoate, Methyl octadecenoate,

			Methyl octadecanoate, Methyl tetracosanoate, Methyl hexacosanoate Methyl Octacosanoate, MethylTriacontanoate, Methyl Dotriacontanoate		
**90.**	Central Anatolia, Turkey<br>Suriye, Turkey<br>Muğla, Turkey<br>Konya Beyşehir Aydancık-Hüseyinler Village, Turkey<br>Eastern Anatolia, Turkey<br>West Mediterranean, Turkey Marmara, Turkey	Ethanolic extract	Apigenin, Luteolin, Emodin, Daidzein, Biochanin, 3-4 dimethoxycinnamic acid, Cafeic acid, *p*-coumaric acid, Myristic acid, Syringic acid, Rosmarinic acid, Chlorogenic acid, Ferrulic acid, Sinapic acid, Protocatheuic Acid, Gallic acid, Benzoic acid, trans-Cinnamic acid, Ellagic acid, *t*-ferulic acid, trans-3-Hydroxy-cinamic acid, Caffeic acid phenethyl ester (CAPE), Protocatechuic), acid (PCA), Cathechin, Kaempherol, Quercetin. Naringenin, Pinobanksin, Pinostrobin, Hesperidin, Chalcon, Trans-chalcone	LC-MS/MS	[89]
**91**	Yozgat, Turkey<br>Izmir, Turkey<br>Kayseri, Turkey<br>Adana, Turkey<br>Erzurum, Turkey<br>Artvin, Turkey	Ethanol extract	cinnamic acid, glucose, benzyl cinnamate, caffeic acid, oleic acid, dimethylallyl caffeate, dehydroabietic acid, cinnamyl cinnamate, pinocembrin, pinobanksin, pinobanksin 3-O-acetate, chrysin, galangin, phenylethyl caffeate, (o) cynnamil caffeate**,** benzoic acid, vanillin,	TLC, GC-MS	[90]

			glucose, p-coumaric acid, benzyl cinnamate, ferulic acid, caffeic acid, oleic acid, dehydroabietic acid, benzyl p-coumarate, benzyl ferulate, coumaroyl glycerol, coumaroyl glycerol (isomer), coumaroyl acetyl glycerol, dicoumarouyl acetyl glycerol, diferuloyl acetyl glycerol, coumaroyl caffeoyl acetyl glycerol.		
**92.**	Erzurum, Anatolia, Turkey	Ethanolic extract	Naringenin, Chrysin, Acacetin, 9-Octadecanoic acid, Hexadecanoic acid, Decanoic acid), Benzoic acid, Ferulic acid, 3,4-Dimethoxycinnamic acid, 3-Hydroxy-4-methoxycinnamic acid, 4-Pentenoic acid, 1,3-Benzenedicarboxylic acid, 2-Propenoic acid, Benzeneacetic acid, 3-Hydroxy-4-niethoxycinnamic acid, Ethyl acetate, Benzyl cinnamate, Benzyl benzoate, Benzeneethanol, Chrysophanol, 4,5-Dimethoxy-2-phenol, α-Cadinol, β-Eudesmol, α-Bisabolol, 4-Vinylphenol, 2-Methoxy-4-vinylphenol, Glycerin, Benzyl alcohol, α-Eudesmol, α-Bisabolol, Chrysophanol, 2-Nonadecanone, 2-Propen-1-one, 4*H*-1-Benzopyran-4-one, 1-Methyl-4-azailuorenone, Nonadecane, Heneicosane, Eicosane, Docosane, 2,5-	GC-MS	[91]

		Diethyl-3,6-dimethylpyrazine, Vanilin, Propanal, 2,3-Dihydro-benzofuran, 4-Hydroxy-2-methoxycinnamaldehyde, Octadecane, Benzene, β-Cadi nene, 2,4-Cycloheptadien-1-one, Benzaldehyde		
**93.**	Mersin, South Anatolia, Turkey Bursa, West Anatolia, Turkey	Phosphoric acid, Butanedioic acid, Propanoic acid, Decanoic acid, Pentanedioic acid, 1-Cyclohexene-1-carboxylic acid, *α*-D-Glucopyranuronic acid, D-Gluconic acid (Palmitic acid), Tetradecanoic acid), Coumaric acid, 2-Propenoic acid (Cinnamic acid), Hydrocinnamic acid, Oleic acid, Linoleic acid, Dehydroabietic acid, Abietic acid), Pinobanksin, Glycerol, Erythritol, D-Glucitol, *myo*-Inositol, Stigmast-22-en-3-ol, Tetrahydrocannabitol, Piperonal, D-Altrose, D-Galactose, Sorbopyranose, *α*-D-Mannopyranose, D-Sorbitol, D-Glucose, D, Mannopyranose, *α*-Atlantone, Pregnanone, Zeylanone, 4*H*-1-Benzopyrane-4-one, Cinnamic acid trimethylester, Cinamic acid 3,4-dimethoxy-tms-ester, Trimethylsilyl 3-methoxy-4-cinnamate, 2*H*-Cyclopentacyclooctene, 6,7-*bis*-1,3-	GC-MS	[92]

			Dimethoxyisoquinoline, Phenanthrene, 4-2-Trimethylsiloxymethyl-1-buten-3-yne, 3-Oxoallobetulane, 10-Ethyl-1,8-diphenyl-anthracene, *N,N-bis*-2-phenylquinolon-4-amine, 1-Methyl-1-*n*-decyloxy-1-silacyclopentane, Cholestane, Hexadecane.		
**94.**	Bursa, Turkey<br>Erzurum- Askale, Turkey<br>Gumushane-Sogutagil, Turkey<br>Trabzon-Caglayan, Turkey	Ethanol extract	Alcohols, Aliphatic acids, Amino acids, Aromatic acids, Aromatic acid esters, aromatic aldehyde, Flavonoids, Ketones, Others, Terpenoids, Vitamin A	GC-MS	[93]
**95.**	Bursa, Turkey<br>Mugla, Turkey<br>Izmir, Turkey<br>Beytepe, Turkey	Ethanol and water extract	Benzoic acid, *p*-Hydroxybenzoic acid, Vanillinic acid, Cinnamic acid, *p*-Coumaric acid, Ferulic acid, Isoferulic acid, caffeic acid, 3,4-dimethoxycinnamic acid, Hexadecanoic acid, Oleic acid, Octadecanoic acid, Benzyl benzoate, Benzyl cinnamate, isopent-3-enyl ferulate, 3,3-dimethylallyl ferulate, Isopent-3-enyl caffeate, 3,3-dimethylallyl caffeate, Benzyl *p*-coumarate, Benzyl ferulate, Benzyl caffeate, Cinnamyl caffeate, Chrysin,	GC-MS	[94]

			Galangin, Sakuratenin, 3-Methylgalangin, Pinostrobin, Pinocembrin, Pinobanksin, Pinobanksin-3-O-acetate, Pinobanksin-3-O-Burenoate, Pinobanksin-3-O-Hexanoate, Pimaric acid, Isopimaric acid, Abietic acid, Dehydroabietic, Fructose, Glucose, Sucrose, Maltose, Glycerol, Vanilin, Mallic acid		
**96.**	Rize, Turkey<br>Kazan, Turkey<br>Mugla, Turkey<br>Tahtakopru, Turkey	Ethanol extract	Benzyl alcohol, Phenyl alchohol, 2-napthalenemethanol, 5-azulenemethanol, 1-napthalenemethanol, 2-pherothrenol, Benzoic acid, Benzenepropanoic acid, 4-pentenoic acid 5-phenyl, Caffeic acid, 2 -Propenoic Acid 3-Phenyl, 2-Propenoic acid 3-(4-methoxyphenyl), 1-phenanthrenecarboxylic acid, Benzaldehyde, Cinnamic acid and its esters, Cinnamyl cinnamate, Benzyl cinnamate, Benzyl benzoate, 1,3-hydroxy-4-methoxycinnamic acid, 1-naphthalene, Fatty acids, Lauric acid, Myristic acid, Palmitic acid, Oleic acid, Stearic acid, Linoleic acid, Cyclohexadecane, Hexadecane, Nonadecane, Octadecane, Octadecanoic acid, 2-propen-1-one, 4H-1-Benzopyran-4-one, Danthron, Narigenin,	GC-MS	[95]

			4'-5'-dihydroxy-7-methoxyflavanone, Chrysin, 3,4',7-trimethoxy flavanone, Ferruginol, Thunbergol, Pinobanksin and its derivatives, Quercetin and its derivatives, Galangine and its derivatives, Apigenin and its derivatives		
**97.**	Hadim-Konya, Turkey	Ethanolic extract	Protocatechuic acid, Catechin, Gentisic acid, Vanilic acid, Cafeic acid, Rutin, p-Coumaric acid, Quercetin 3-O-galactoside (Hyperside), Ellagic acid, Apigenin 7-O-apioglucoside (Apiin), Ferulic acid, Kaempferol 3-O-glucoside (Astragalin), Apigenin-7-O-glucoside, Resveratrol, Hesperetin, Luteolin, Naringenin, Apigenin, Genistein, Chrysin, Pinocembrin, Galangin	HPLC, UPLC-MS	[2]
**98**	China		Cinnamic acid, Caffeic acid, 2,5-Dimethyl-7-hydroxychromone, Isoferulic acid, Dimethylcaffeic acid, Benzyl caffeate, Luteolin, Chrysoeriol, 3′ ,5,6,7-Tetrahydroxy-4′ -methoxyisoflavone, Neobavaisoflavone, Jaceosidin, Xanthomicrol, 3′ -Methoxydaidzin, Genistin, Isoaloeresin D, 3′-Deoxysappanol	LC-MS/MS QTOF	[96]

**99**	China	Water and ethanol extract	chrysin, galandin, kaempferol, 3-O-methylkaempferol	Thin layer chromatography (TLC) and reversed-phase column chromatography	[97]
**100**	Beijing, China	Ethanol extract	3,4-Dihydroxybenzaldehyde, vanillic acid, caffeic acid, p-coumaric acid, ferulic acid, isoferulic acid, benzoic acid, 3,4-dimethoxycinnamic acid, cinnamic acid, 4- methoxycinnamic acid, cinnamylideneacetic acid, 5-methoxy pinobanksin, pinobanksin, quercetin, alpinetin, kaempferol, cinnamylideneacetic acid, apigenin, isorhamnetin, pinocembrin, benzyl caffeate, pinobanksin-3-O- acetate, chrysin, phenethyl caffeate, galangin, benzyl p-coumarate, pinostrobin, tectochrysin, and cinnamyl cinnamate.	HPLC	[98]

Table S2. SMILES codes of all test compounds

Compound Name	Number Code	SMILES
**2,5-Dihyrothiophene**	NCIRI-1	C1C=CCS1
**Dotriacontene**	NCIRI-2	CCCCCCCCCCCCCCCCCCCCCCCCCCCCCCC=C
**Methyl tetradecanoate**	NCIRI-3	CCCCCCCCCCCCCC(=O)OC
**Tritriacontene**	NCIRI-4	CCCCCCCCCCCCCCCCCCCCCCCCCCCCCCCC=C
**(-)-Elema-1,3,11(13)-trien-12-al**	NCIRI-6	CC(=C)C1CC(CCC1(C)C=C)C(=C)C=O
**(-)-MOLLISACACIDIN**	NCIRI-7	Oc1ccc3c(c1)O[C@H](c2ccc(O)c(O)c2)[C@@H](O)[C@@H]3O
**(+)-cis-Khellactone**	NCIRI-8	CC1([C@@H]([C@@H](C2=C(O1)C=CC3=C2OC(=O)C=C3)O)O)C
**(+)-medicarpin**	NCIRI-9	COC1=CC2=C(C=C1)C3COC4=C(C3O2)C=CC(=C4)O
**(+)-MOLLISACACIDIN**	NCIRI-10	C1=CC(=C(C=C1C2C(C(C3=C(O2)C=C(C=C3)O)O)O)O)O
**(+)-oxypeucedanin hydrate**	NCIRI-11	CC(C)([C@@H](COC1=C2C=CC(=O)OC2=CC3=C1C=CO3)O)O
**(+)-vesticarpan**	NCIRI-12	COc4ccc2c(OC3c1ccc(O)cc1OCC23)c4O

**(±)-(E)-4'-methoxy-4,2'-dihydroxy-3'-(2'',3''-dihydroxy-3''-methylbutyl)-chalcone**	NCIRI-13	COc2ccc(C(=O)C=Cc1ccc(O)cc1)c(O)c2CCC(O)C(C)(C)O
**(±)-(E)-4'-methoxy-4,3'',4''-trihydroxy-2'',2''-dimethyldihydropyrano-(2',3')-chalcone**	NCIRI-14	COc2ccc(C(=O)C=Cc1ccc(O)cc1)c3OC(C)(C)C(O)C(O)c23
**(±)-(E,E)-4,2',4'-trihydroxy -3'-(5''-hydroxy-3'',7''-dimethyloct-2'',6''-dienyl)-chalcone**	NCIRI-15	CC(C)=CC(O)CC(C)=CCCc2c(O)ccc(C(=O)C=Cc1ccc(O)cc1)c2O
**(1'S)-2-trans,4-trans-abscisic acid**	NCIRI-16	CC(C=C[C@@]1(O)C(C)=CC(=O)CC1(C)C)=CC(=O)O
**(22Z,24E)-3-oxocycloart-22,24-dien-26-oic acid**	NCIRI-17	CC(=CC=CC(C)C2CCC3(C)C1CCC45CC1(CCC23C)C4CCC(=O)C5(C)C)C(=O)O
**(23E)-27-nor-3beta-hydroxycycloart-23-en-25-one**	NCIRI-18	[H][C@]4([C@H](C)CC=CC(C)=O)CC[C@@]5(C)[C@]2([H])CC[C@@]1([H])C(C)(C)[C@H](O)CC[C@@]13C[C@@]23CC[C@]45C
**(24E)-3beta-hydroxycycloart-24-en-26-al**	NCIRI-19	[H]C(=O)C(C)=CCC[C@@H](C)[C@@]4([H])CC[C@@]5(C)[C@]2([H])CC[C@@]1([H])C(C)(C)[C@H](O)CC[C@@]13C[C@@]23CC[C@]45C
**(24E)-3-oxo-27,28-dihydroxycycloart-24-en-26-oic acid**	NCIRI-20	CC(CCC=C(CO)C(=O)O)C2CCC3(C)C1CCC45CC1(CCC23C)C4CCC(=O)C5(C)CO
**(2R)-7,4′-Dihydroxy-5-methoxy-8-methylflavane**	NCIRI-21	COc2cc(O)c(C)c3O[C@@H](c1ccc(O)cc1)CCc23
**(2R,3R)-pinobanksin 3-(2-methyl)-butyrate**	NCIRI-22	CCC(C)C(=O)O[C@H]2C(=O)c1c(O)cc(O)cc1O[C@@H]2c3ccccc3
**(2R,3R)-pinobanksin 3-isobutyrate**	NCIRI-23	CC(C)C(=O)O[C@@H]1[C@H](OC2=CC(=CC(=C2C1=O)O)O)C3=CC=CC=C3
**(2S)-5,7,4′-trihydroxy-8,3′-diprenylflavanone**	NCIRI-24	CC(C)=CCc3cc([C@@H]2CC(=O)c1c(O)cc(O)c(CC=C(C)C)c1O2)ccc3O
**(2S)-5,7,4′-trihydroxy-8-prenylflavanone**	NCIRI-25	CC(C)=CCc2c(O)cc(O)c3C(=O)C[C@@H](c1ccc(O)cc1)Oc23
**(2S)-5,7-dihydroxy-4′-methoxy-8,3-diprenylflavanone**	NCIRI-26	COc3ccc([C@@H]2CC(=O)c1c(O)cc(O)c(CC=C(C)C)c1O2)cc3CC=C(C)C

**(2S)-5,7-dihydroxy-4′-methoxy-8-prenylflavanone**	NCIRI-27	COc3ccc([C@@H]2CC(=O)c1c(O)cc(O)c(CC=C(C)C)c1O2)cc3
**(2S)-7- Methoxyflavanone**	NCIRI-28	COC1=CC2=C(C=C1)C(=O)CC(O2)C3=CC=CC=C3
**(2'S,3'R)-(+)-vaginidol**	NCIRI-29	CC(C)(O)[C@H]3Oc2ccc1ccc(=O)oc1c2[C@H]3O
**(3S)-7,4′-Dihydroxy-5-methoxyhomoisoflavane**	NCIRI-30	COc2cc(O)cc3OCC(Cc1ccc(O)cc1)Cc23
**(4S,6S)-4-Hydroxy-3-methoxy-6-(1-phenyl-2-propenyl)-2-cyclohexene-1-one**	NCIRI-31	[H][C@@]2(C(C=C)c1ccccc1)C[C@@H](O)C(OC)=CC2=O
**(5R,8S,9S,10R,13R,14S,17R,20R)-27-methoxycarbonyloxy- mangiferonic acid**	NCIRI-32	[H][C@]4([C@H](C)CCC=C(OC(=O)OC)C(=O)O)CC[C@@]5(C)[C@]2([H])CC[C@@]1([H])C(C)(C)C(=O)CC[C@@]13C[C@@]23CC[C@]45C
**(E)-1-(5-Bromothiophen-2-yl)-3-[4-(dimethylamino)phenyl]prop-2-en-1-one (TB5)**	NCIRI-33	CN(C)C1=CC=C(C=C1)/C=C/C(=O)C2=CC=C(S2)Br
**(E)-Cinnamaldehyde**	NCIRI-34	C1=CC=C(C=C1)C=CC=O
**(E)-cinnamyl (E)-cinnamate**	NCIRI-35	O=C(C=Cc1ccccc1)OCC=Cc2ccccc2
**(E)-cinnamyl (E)-ferulate**	NCIRI-36	COc2cc(C=CC(=O)OCC=Cc1ccccc1)ccc2O
**(E)-cinnamyl-(E)- cinnamylidenate**	NCIRI-37	O=C(C=CC=Cc1ccccc1)OCC=Cc2ccccc2
**(E,E,E)-4,2',4'-trihydroxy-3'-(7''-hydroxy-3'',7''-dimethyloct-2'',5''-dienyl)-chalcone**	NCIRI-38	CC(=CCCc2c(O)ccc(C(=O)C=Cc1ccc(O)cc1)c2O)CC=CC(C)(C)O
**(R) Pinocembrin**	NCIRI-39	C1[C@@H](OC2=CC(=CC(=C2C1=O)O)O)C3=CC=CC=C3
**(RR)-(+), 3,3,4-trimethyl-4-p-tolyl-cyclopentanol**	NCIRI-40	Cc2ccc([C@]1(C)C[C@H](O)CC1(C)C)cc2
**(RR)-(+)-3'-senecioylkhellactone**	NCIRI-41	CC(C)=CC(=O)OC[C@@H]3[C@H](O)c1c(ccc2ccc(=O)oc12)OC3(C)C

**(S)-(-)-selidin**	NCIRI-42	CC=C(C)C(=O)O[C@H]3Cc1c(ccc2ccc(=O)oc12)OC3(C)C
**(S)-(+)-marmesin**	NCIRI-43	CC(C)([C@@H]1CC2=C(O1)C=C3C(=C2)C=CC(=O)O3)O
**(SS)- or (RR)-2,3-hexanediol**	NCIRI-44	CCCC(C(C)O)O
**(SS)-(-)-isolaserpitin**	NCIRI-45	C/C=C(/C)\C(=O)OC1C(C2=C(C=CC3=C2OC(=O)C=C3)OC1(C)C)O
**[6]-Dehydrogingerdione**	NCIRI-46	CCCCCC(=O)CC(=O)C=CC1=CC(=C(C=C1)O)OC
**1-(5-ethenyltetrahydro-5-methyl-2-furanyl)- 1-methylethanol**	NCIRI-47	C=C[C@@]1(C)CC[C@@H](C(C)(C)O)O1
**1,2,3,4,5-Pentakis-O-(trimethylsilyl), ribitol**	NCIRI-48	C[Si](C)(C)OCC(C(C(CO[Si](C)(C)C)O[Si](C)(C)C)O[Si](C)(C)C)O[Si](C)(C)C
**1,2,3,5-Tetrakis-O-(trimethylsilyl), arabinofuranose**	NCIRI-49	C[Si](C)(C)OC[C@@H]1[C@H]([C@H](C(O1)O[Si](C)(C)C)O[Si](C)(C)C)O[Si](C)(C)C
**1,2,3-Propanetriol (glycerol)**	NCIRI-50	C(C(CO)O)O
**1,2,-Benzenedicarboxylic acid**	NCIRI-51	O=C2c1ccccc1C(=O)C(O)C2O
**1,2-Benzenedicarboxylic acid**	NCIRI-52	C1=CC=C(C(=C1)C(=O)O)C(=O)O
**1,2-Benzenedicarboxylic acid, bis(8-methyl nonyl) ester**	NCIRI-53	CC(C)CCCCCCCOC(=O)C1=CC=CC=C1C(=O)OCCCCCCCC(C)C
**1,2-Benzenedicarboxylic acid, butyl 8-methylonyl ester**	NCIRI-54	CCCCOC(=O)c1ccccc1C(=O)OCCCCCCCC(C)C
**1,2-Benzenedicarboxylic acid, diisodecyl ester**	NCIRI-55	CCCCCCCCCCOC(=O)c1ccccc1C(=O)OCCCCCCCCCC
**1,2-Dihydro-4,6-dimethyl-2-oxo, nicotinic acid**	NCIRI-56	CC1=CC(=C(C(=O)N1)C(=O)O)C
**1,3 benzenedicarboxylic acid**	NCIRI-57	C1=CC(=CC(=C1)C(=O)O)C(=O)O
**1,3,4,5,6-Pentakis-O-(trimethylsilyl)-, O-methyloxime, d-fructose**	NCIRI-58	CON=C(CO[Si](C)(C)C)C(C(C(CO[Si](C)(C)C)O[Si](C)(C)C)O[Si](C)(C)C)O[Si](C)(C)C

**1,3,6-Octatriene, 3,7-dimethyl-, (E)-**	NCIRI-59	CC(=CCC=C(C)C=C)C
**1,4- Dihydrophenanthrene**	NCIRI-60	C1C=CCC2=C1C=CC3=CC=CC=C23
**1,4-Benzenediol (hydroquinone)**	NCIRI-61	C1=CC(=CC=C1O)O
**1,5,5-Trimethyl-6-methylene-cyclohexene**	NCIRI-62	CC1=CCCC(C1=C)(C)C
**10,11-Dihydroxydracaenone C**	NCIRI-64	C1C2C[C@]3(C=CC(=O)C=C3OC2)C4=CC(=C(C=C41)O)O
**10-hydroxybenzo[j]fluoranthene**	NCIRI-65	Oc5ccc3c(ccc4c2cccc1cccc(c12)c34)c5
**11-Dien-2-one, 4.beta.H,5.alpha.-ermophila-1(10)**	NCIRI-66	C=C(C)[C@@H]2CCC1=CC(=O)C[C@@H](C)[C@]1(C)C2
**11-Eicosanoic acid**	NCIRI-67	CCCCCCCC/C=C\CCCCCCCCCC(=O)O
**12 Hydroxysteric acid phenacyl ester**	NCIRI-68	CCCCCCC(CCCCCCCCCCC(=O)OCC(=O)C1=CC=CC=C1)O
**13-epi-torulosal**	NCIRI-69	[H][C@@]12CCC(=C)[C@H](CC[C@@](C)(O)C=C)[C@@]1(C)CCC[C@]2(C)C=O
**13-tetradecen-11-yn-1-ol**	NCIRI-70	C=CC#CCCCCCCCCCCO
**1-Heptatriacotanol**	NCIRI-71	CCCCCCCCCCCCCCCCCCCCCCCCCCCCCCCCCCCCO
**1-methyl-4-azafluorenone**	NCIRI-72	Cc2ccnc3c1ccccc1c(=O)c23
**1-napthalene**	NCIRI-73	C1=CC=C2C=CC=CC2=C1
**1-napthalenemethanol**	NCIRI-74	C1=CC=C2C(=C1)C=CC=C2CO
**1-phenanthrenecarboxylic acid**	NCIRI-75	C1=CC=C2C(=C1)C=CC3=C2C=CC=C3C(=O)O
**2-(1-Phenylprop-2-enyl)benzene-1,4-diol**	NCIRI-76	C=CC(C1=CC=CC=C1)C2=C(C=CC(=C2)O)O
**2-(3-Bromo-3-buten-1-yl)-1,3-dioxolane**	NCIRI-77	C=C(CCC1OCCO1)Br
**2(5H)-Furanone, 5,5-diphenyl**	NCIRI-78	C1=CC=C(C=C1)C2(C=CC(=O)O2)C3=CC=CC=C3

**2-(Methylenecyclopropyl) ethyl ester acrylic acid**	NCIRI-79	C=CC(=O)OCCC1CC1=C
**2,3,4-Trihydroxy butyric acid (tetronic acid)**	NCIRI-80	C1C(=O)COC1=O
**2,3-diethynylbut-2-ene-1,4-diol**	NCIRI-81	C#CC(CO)=C(C#C)CO
**2,3-dihydro-benzofuran**	NCIRI-82	C1COC2=CC=CC=C21
**2,3-Dihydroxypropanoic acid (glyceric acid)**	NCIRI-83	C(C(C(=O)O)O)O
**2,4-dihydroxy-2,5-dimethyl-3(2h)furan-3-one**	NCIRI-84	CC1=C(C(=O)C(O1)(C)O)O
**2,5 Dimethoxyterephthalic acid**	NCIRI-85	COC1=CC(=C(C=C1C(=O)O)OC)C(=O)O
**2,5-diethyl-3,6-dimethylpyrazine**	NCIRI-86	CCC1=C(N=C(C(=N1)C)CC)C
**2,5-Dimethyl-7-hydroxychromone**	NCIRI-87	CC1=CC(=CC2=C1C(=O)C=C(O2)C)O
**2,5-furandione or succinic anhydride**	NCIRI-88	C1CC(=O)OC1=O
**2',6'-Dihydroxy-4'-methoxychalcone**	NCIRI-89	COC1=CC(=C(C(=C1)O)C(=O)/C=C/C2=CC=CC=C2)O
**20,40,60-Trihydroxy chalcone (pinocembrin chalcone)**	NCIRI-91	O=C(C=Cc1ccccc1)c2c(O)cc(O)cc2O
**23-hydroxyisomangiferolic acid**	NCIRI-92	[H][C@]4([C@H](C)CC(O)C=C(C)C(=O)O)CC[C@@]5(C)[C@]2([H])CC[C@@]1([H])C(C)(C)C(O)CC[C@@]13C[C@@]23CC[C@]45C
**23-hydroxymangiferolic acid**	NCIRI-93	[H][C@]4([C@H](C)CCC=C(CO)C(=O)O)CC[C@@]5(C)[C@]2([H])CC[C@@]1([H])C(C)(C)[C@@H](O)CC[C@@]13C[C@@]23CC[C@]45C
**23-hydroxymangiferonic acid**	NCIRI-94	C[C@H](CC(/C=C(\C)/C(=O)O)O)[C@H]1CC[C@@]2([C@@]1(CC[C@]34[C@H]2CC[C@@H]5[C@]3(C4)CCC(=O)C5(C)C)C)C
**24-(Z)-3-oxolanosta-1,7,24-trien-26-oic acid**	NCIRI-95	CC(=CCCC[C@H]3CC[C@@]4(C)C2=CC[C@H]1C(C)(C)C(O)=CC[C@]1(C)C2CC[C@]34C)C(=O)O
**24-Methylenecycloartanol**	NCIRI-96	CC(C)C(=C)CCC(C)C1CCC2(C1(CCC34C2CCC5C3(C4)CCC(C5(C)C)O)C)C

**27-acetoxymangiferolic acid**	NCIRI-97	[H][C@]4([C@H](C)CCC=C(COC(=O)O)C(=O)O)CC[C@@]5(C)[C@]2([H])CC[C@@]1([H])C(C)(C)C(=O)CC[C@@]13C[C@@]23CC[C@]45C
**27-hydroxymangiferolic acid**	NCIRI-98	C[C@H](CC/C=C(\CO)/C(=O)O)[C@H]1CC[C@@]2([C@@]1(CC[C@]34[C@H]2CC[C@@H]5[C@]3(C4)CC[C@@H](C5(C)C)O)C)C
**27-hydroxymangiferoNic acid**	NCIRI-99	C[C@H](CC/C=C(\CO)/C(=O)O)[C@H]1CC[C@@]2([C@@]1(CC[C@]34[C@H]2CC[C@@H]5[C@]3(C4)CCC(=O)C5(C)C)C)C
**28-hydroxymangiferonic acid**	NCIRI-100	CC(CCC=C(C)C(=O)O)C1CCC2(C1(CCC34C2CCC5C3(C4)CCC(=O)C5(C)CO)C)C
**2-Amino-1-(3-hydroxy-4-methoxyphenyl) ethanone**	NCIRI-101	COC1=C(C=C(C=C1)C(=O)CN)O
**2-courmaranone**	NCIRI-102	C1C2=CC=CC=C2OC1=O
**2-furanmethanol**	NCIRI-103	C1=COC(=C1)CO
**2H-2,4a-Ethanonaphthalene,1,3,4,5,6,7-hexahydro-2,5,5-trimethyl-**	NCIRI-104	CC1(CCC=C2C13CCC(C2)(CC3)C)C
**2-Hydroxy acetic acid**	NCIRI-105	C(C(=O)O)O
**2-Hydroxy malonic acid**	NCIRI-106	C(C(=O)O)(C(=O)O)O
**2-Hydroxy-5-methylbenzaldehyde**	NCIRI-107	CC1=CC(=C(C=C1)O)C=O
**2-hydroxybenzyl alcohol**	NCIRI-108	C1=CC=C(C(=C1)CO)O
**2-Hydroxybutanedioic acid (malic acid)**	NCIRI-109	C(C(C(=O)O)O)C(=O)O
**2'-hydroxyformononetin**	NCIRI-110	COC1=CC(=C(C=C1)C2=COC3=C(C2=O)C=CC(=C3)O)O
**2-Hydroxypropanoic acid (lactic acid)**	NCIRI-111	CC(C(=O)O)O
**2-Methoxy-4-vinylphenol**	NCIRI-112	COC1=C(C=CC(=C1)C=C)O
**2-Methyl, 1-penten-3-yne**	NCIRI-113	CC#CC(=C)C
**2-methyl-2-butenol**	NCIRI-114	C/C=C(/C)\CO

**2-Methyl-2-butenyl-trans-4-coumarate**	NCIRI-115	CCC=CC1(C)C=C(O)C=CC1C=CC(=O)O
**2-Methyl-2-butenyl-trans-caffeate**	NCIRI-116	Oc1ccc(/C=C/C(=O)OCC(\C)=C\C)cc1O
**2-Methylpropanoyl**	NCIRI-117	[CH2-]C(=C)C#[O+]
**2-naphtalenemethanol**	NCIRI-118	C1=CC=C2C=C(C=CC2=C1)CO
**2-Naphthalenemethanol, 1,2,3,4,4a,5,6,7-octahydro-α,α,4a,8-tetramethyl-, (2R-cis)-**	NCIRI-119	CC2=C1C[C@H](C(C)(C)O)CC[C@@]1(C)CCC2
**2-nonadecanone**	NCIRI-120	CCCCCCCCCCCCCCCCC(=O)C
**2-Phenylpropyl propanoate**	NCIRI-122	CCC(=O)OCC(C)C1=CC=CC=C1
**2-propen-1-one**	NCIRI-123	C=CC=O
**2-propenoic acid**	NCIRI-124	C=CC(=O)O
**2-Propenoic acid, 3-phenyl-, ethyl ester (ethyl cinnamate)**	NCIRI-125	CCOC(=O)C=CC1=CC=CC=C1
**2-Propenoic acid, 3-phenyl-, ethylester, (E)-**	NCIRI-126	CCOC(=O)C=Cc1ccccc1
**3(3,4-Dihydroxyphenyl)-2-propenoic acid (caffeic acid)**	NCIRI-128	C1=CC(=C(C=C1C=CC(=O)O)O)O
**3(3,4-Dihydroxyphenyl)-2-propenoic acid, methyl ester**	NCIRI-129	COC(=O)C=Cc1ccc(O)c(O)c1
**3(3,4-Dimethoxyphenyl)-2-propenoic acid (3,4-dimethoxy cinnamic acid)**	NCIRI-130	COC1=C(C=C(C=C1)/C=C/C(=O)O)OC
**3(3,4-Dimethoxyphenyl)-2-propenoic acid, methyl ester**	NCIRI-131	COC(=O)C=Cc1ccc(OC)c(OC)c1
**3(3-Hydroxy-4-methoxyphenyl)-2-propenoic acid (isoferulic acid)**	NCIRI-132	C1=CC(=CC=C1C=CC(=O)O)O

**3(3-Methoxy, 4-hydroxyphenyl)-2-propenoic acid (isomer 1)**	NCIRI-133	COc1cc(C=CC(=O)O)ccc1O
**3(3-Methoxy, 4-hydroxyphenyl)-2-propenoic acid, methyl ester**	NCIRI-134	COC(=O)C=Cc1ccc(O)c(OC)c1
**3(4-Methoxy, 3-hydroxyphenyl)-2-propenoic acid, methyl ester**	NCIRI-135	COC(=O)C=Cc1ccc(OC)c(O)c1
**3,12-Oleandione**	NCIRI-136	CC1(CCC2(CCC3(C(C2C1)C(=O)CC4C3(CCC5C4(CCC(=O)C5(C)C)C)C)C)C)C
**3,3-dimethylallyl caffeate**	NCIRI-137	CC(C)=CCOC(=O)C=Cc1ccc(O)c(O)c1
**3,3-dimethylpentane**	NCIRI-138	CCC(C)(C)CC
**3,3-Diphenyl-cyclopropene**	NCIRI-139	C1=CC1(c2ccccc2)c3ccccc3
**3,4',7-trimethoxy flavanone**	NCIRI-143	COc3ccc2C(=O)C(O)[C@@H](c1ccc(OC)c(OC)c1)Oc2c3
**3,4-dihydro-2-(3,4-dihydroxyphenyl)-2H-chromene-3,7-diol**	NCIRI-144	Oc3ccc2CC(O)[C@@H](c1ccc(O)c(O)c1)Oc2c3
**3',4'-dihydroxy-4-methoxydalbergione**	NCIRI-145	COC1=CC(=O)C(=CC1=O)C(C=C)C2=CC(=C(C=C2)O)O
**3,4-dihydroxybenzaldehyde**	NCIRI-146	C1=CC(=C(C=C1C=O)O)O
**3,4-dihydroxybenzoic acid**	NCIRI-147	C1=CC(=C(C=C1C(=O)O)O)O
**3,4-dimethoxycaffeic acid cinnamyl ester**	NCIRI-148	COc2ccc(C=CC(=O)OCC=Cc1ccccc1)cc2OC
**3,4-Dimethoxystyrene**	NCIRI-149	COC1=C(C=C(C=C1)C=C)OC
**3,4-dimethyl caffeic acid**	NCIRI-150	CC1(C=CC(=CC1(C)O)C=CC(=O)O)O
**3,4-Octadiene, 7-methyl-**	NCIRI-151	CCC=C=CCC(C)C
**3′,8- diprenylnaringenin**	NCIRI-154	CC(=CCC1=C(C=CC(=C1)C2=CC(=O)C3=C(C=C(C(=C3O2)CC=C(C)C)O)O)O)C

**3′-Methoxydaidzin**	NCIRI-157	COC1=C(C=CC(=C1)C2=COC3=C(C2=O)C=CC(=C3)O[C@H]4[C@@H]([C@H]([C@@H]([C@H](O4)CO)O)O)O)O
**3alpha,22-dihydroxycycloart-24-en-26-oic acid**	NCIRI-158	[H][C@@]24CC[C@@]1(C)[C@](C)(C)[C@@H](O)CC[C@@]13C[C@@]23CC[C@]5(C)[C@@H]([C@H](C)C(O)CC=C(CO)C(=O)O)CC[C@@]45C
**3alpha,27-dihydroxycycloart-24-en-26-oic acid**	NCIRI-159	[H][C@@]24CC[C@@]1(C)[C@](C)(C)[C@@H](O)CC[C@@]13C[C@@]23CC[C@]5(C)[C@@H]([C@H](C)CCC=C(CO)C(=O)O)CC[C@@]45C
**3B,23-dihydroxycycloart-24-en-26-oic acid**	NCIRI-160	[H][C@@]24CC[C@@]1(C)[C@](C)(C)[C@H](O)CC[C@@]13C[C@@]23CC[C@]5(C)[C@@H]([C@H](C)CC(O)C=C(CO)C(=O)O)CC[C@@]45C
**3B,27-dihydroxycycloart-24-en-26-oic acid**	NCIRI-161	[H][C@@]24CC[C@@]1(C)[C@](C)(C)[C@H](O)CC[C@@]13C[C@@]23CC[C@]5(C)[C@@H]([C@H](C)CCC=C(CO)C(=O)O)CC[C@@]45C
**3'-Deoxysappanol**	NCIRI-162	C1[C@@]([C@H](C2=C(O1)C=C(C=C2)O)O)(CC3=CC=C(C=C3)O)O
**3'-geranylnaringenin**	NCIRI-164	CC(C)=CCCC(C)=CCCc3cc(C2CC(=O)c1c(O)cc(O)cc1O2)ccc3O
**3-Geranyloxy-1,7-dihydroxyxanthone**	NCIRI-165	CC(=CCC/C(=C/COC1=CC(=C2C(=C1)OC3=C(C2=O)C=C(C=C3)O)O)/C)C
**3-Hydroxybutanoic acid**	NCIRI-168	CC(CC(=O)O)O
**3-Methoxy,4-hydroxybenzoic acid (isovanillic acid)**	NCIRI-169	COC1=C(C=C(C=C1)C(=O)O)O
**3-Methoxy-4,5-methylenedioxybenzaldehyde**	NCIRI-170	COC1=CC(=CC2=C1OCO2)C=O
**3-methyl-2-butenyl ferulate**	NCIRI-173	CC(=CCOC(=O)/C=C/C1=CC(=C(C=C1)O)OC)C
**3-Methyl-2-butenyl-trans-4-coumarate**	NCIRI-174	CCC=Cc1c(C)c(O)ccc1C=CC(=O)O
**Phenylethyl trans-4-coumarate**	NCIRI-175	O=C(C=Cc1ccc(O)cc1)OCCc2ccccc2
**3-Methyl-2-butenyl-trans-caffeatE**	NCIRI-176	CC(=CCOC(=O)/C=C/C1=CC(=C(C=C1)O)O)C
**3-Methyl-2-butenyl-trans-ferulate**	NCIRI-177	CCC=CC1C(C=CC(=O)O)=CC=C(O)C1(C)OC
**3-methyl-3-buten-1-ol**	NCIRI-178	CC(=C)CCO
**3-Methyl-3-butenyl-trans-caffeate**	NCIRI-179	CC(=C)CCOC(=O)/C=C/C1=CC(=C(C=C1)O)O

**3-Methyl-3-butenyl-trans-iso ferulate**	NCIRI-180	C=C(C)CCOC(=O)/C=C\c1ccc(OC)c(O)c1
**3-methyl-4-isopropylphenol**	NCIRI-181	CC1=C(C=CC(=C1)O)C(C)C
**3-Methylbutanoyl**	NCIRI-182	[CH2-]C(=C)CC#[O+]
**3-methylethergalangin**	NCIRI-183	COC1=C(OC2=CC(=CC(=C2C1=O)O)O)C3=CC=CC=C3
**3-O-acetyl-11-oxoursolic acid**	NCIRI-184	C[C@@H]1CC[C@@]2(CC[C@@]3(C(=CC(=O)[C@H]4[C@]3(CC[C@@H]5[C@@]4(CC[C@@H](C5(C)C)OC(=O)C)C)C)[C@@H]2[C@H]1C)C)C(=O)O
**3-Phenyl-1-propanol**	NCIRI-185	C1=CC=C(C=C1)CCCO
**4',5-dihydroxy-7-methoxyflavanone**	NCIRI-187	COC1=CC(=C2C(C(=O)C(OC2=C1)C3=CC=CC=C3)O)O
**4,5-dimethoxy-2-phenol**	NCIRI-188	COc1ccc(O)cc1OC
**4,8,13-Cyclotetradecatriene-1,3-diol, 1,5,9-trimethyl-12-(1-methylethyl)-**	NCIRI-190	CC1=CCCC(=CC(CC(C=CC(CC1)C(C)C)(C)O)O)C
**4-bH,5a-Eremophil-1 (10)-ene**	NCIRI-191	C=C(C)C2CCC1CCCC(C)C1(C)C2
**4-Ethylformanilide**	NCIRI-192	CCC1=CC=C(C=C1)NC=O
**4H-1-benzopyran-4-one**	NCIRI-193	C1=CC=C2C(=C1)C(=O)C=CO2
**4-hydroxy-2-methoxycinnamaldehyde**	NCIRI-194	COC1=C(C=CC(=C1)O)C=CC=O
**4-Hydroxybenzaldehyde**	NCIRI-195	C1=CC(=CC=C1C=O)O
**4-Hydroxybenzoic acid**	NCIRI-196	C1=CC(=CC=C1C(=O)O)O
**4-hydroxyderricin**	NCIRI-197	CC(=CCC1=C(C=CC(=C1O)C(=O)/C=C/C2=CC=C(C=C2)O)OC)C
**4-hydroxymedicarpin**	NCIRI-198	COC1=CC2=C(C=C1)C3COC4=C(C3O2)C=CC(=C4O)O
**4'-Methoxy-2',3, 7-trihydroxyisoflavanon**	NCIRI-200	COC1=CC(=C(C=C1)C2(COC3=C(C2=O)C=CC(=C3)O)O)O
**4-methoxycinnamic acid**	NCIRI-201	COC1=CC=C(C=C1)/C=C/C(=O)O

**4-methoxycinnamic acid cinnamyl ester**	NCIRI-202	COc2ccc(C=CC(=O)OCC=Cc1ccc(O)cc1)cc2
**4-methoxydalbergione**	NCIRI-203	COC1=CC(=O)C(=CC1=O)C(C=C)C2=CC=CC=C2
**4-Methyl-2-(1-ethylethenyl)-1-cyclopentene-1-carboxaldehyde**	NCIRI-204	C=C(C)C1=C(C=O)CC(C)C1
**4-pentenoic acid**	NCIRI-205	C=CCCC(=O)O
**4-pentenoic acid, 5-phenyl**	NCIRI-206	C1=CC=C(C=C1)C=CCCC(=O)O
**4-vinylphenol**	NCIRI-207	C=CC1=CC=C(C=C1)O
**5,7-Dihydroxy-4'-methoxy-8-C-prenylflavanone**	NCIRI-219	CC(=CCC1=C2C(=C(C=C1O)O)C(=O)CC(O2)C3=CC=C(C=C3)OC)C
**5,7-Dihyroxy-3-(iso)butanoyloxyflavanone (pinobanksin-3-isobutanoate)**	NCIRI-221	CC(C)C(=O)OC1C(OC2=C(C1=O)C(=CC(=C2)O[Si](C)(C)C)O[Si](C)(C)C)C3=CC=CC=C3
**5,7-Dihyroxy-3-(iso)pentanoyloxyflavanone (pinobankin-3-isopentanoate)**	NCIRI-222	CC(C)CC(=O)O[C@H]2C(=O)c1c(O)cc(O)cc1O[C@@H]2c3ccccc3
**5,7-Dihyroxy-3-propanoyloxyflavanone (pinobanksin-3-propanoate)**	NCIRI-223	CCC(=O)OC1C(OC2=CC(=CC(=C2C1=O)O)O)C3=CC=CC=C3
**5-azulenemethanol**	NCIRI-225	C1=CC2=CC=CC2=CC(=C1)CO
**5-heptylresorcinol**	NCIRI-226	CCCCCCCC1=CC(=CC(=C1)O)O
**5-octadecene**	NCIRI-230	CCCCCCCCCCCC=CCCCC
**5-Phenoxymethyl-1,3,4-thiadiazol-2-amine**	NCIRI-231	C1=CC=C(C=C1)OCC2=NN=C(S2)N
**5-phenyl-4-pentenoic acid**	NCIRI-232	C1=CC=C(C=C1)/C=C/CCC(=O)O
**5-phenylthiazolidine**	NCIRI-233	C1C(SCN1)C2=CC=CC=C2

**6(Z)9E-Heptadecadine**	NCIRI-234	CCCCCC=CCCCCCCCCCC
**6,7-dihydroxyflavanon**	NCIRI-235	C1C(OC2=CC(=C(C=C2C1=O)O)O)C3=CC=CC=C3
**6-cinnamylchrysin**	NCIRI-236	C1=CC=C(C=C1)/C=C/CC2=C(C3=C(C=C2O)OC(=CC3=O)C4=CC=CC=C4)O
**6-c-methyl quercetin**	NCIRI-237	CC1=C(C2=C(C=C1O)OC(=C(C2=O)O)C3=CC(=C(C=C3)O)O)O
**6-Dehydrogingerdione**	NCIRI-238	CCCCCC(=O)C=C(C=CC1=CC(=C(C=C1)O)OC)O
**6-Epiangustifolin**	NCIRI-239	CC12CCCC3(C1C(OC2)OC)COC(=O)C45C3C(CC(C4)C(=C)C5=O)O
**6-Hydroxy-1-oxogermacr-4,10(15),11(13)-trien-12,8-olide**	NCIRI-240	CC1=CC(C2C(CC(=C)C(=O)CC1)OC(=O)C2=C)O
**6-hydroxy-3-methoxy-6-(3-phenyl-2-propenyl)-2-cyclehexane-1-one**	NCIRI-241	COC2=CC(=O)[C@](O)(CC=Cc1ccccc1)CC2
**7,4′-Dihydroxyflavanone (liquiritigenin)**	NCIRI-243	C1[C@H](OC2=C(C1=O)C=CC(=C2)O)C3=CC=C(C=C3)O
**7,4′-Dihydroxyhomoisoflavane**	NCIRI-244	C1C(C(=O)C2=C(O1)C=C(C=C2)O)CC3=CC=C(C=C3)O
**7-Geranyloxy-1,3-dihydroxyxanthone**	NCIRI-245	CC(=CCC/C(=C/COC1=CC2=C(C=C1)OC3=CC(=CC(=C3C2=O)O)O)/C)C
**7-hydroxycoumarin**	NCIRI-247	C1=CC(=CC2=C1C=CC(=O)O2)O
**7-hydroxyflavanone**	NCIRI-248	C1C(OC2=C(C1=O)C=CC(=C2)O)C3=CC=CC=C3
**7-O-methyl-8-prenylnaringenin**	NCIRI-249	CC(=CCC1=C(C=C(C2=C1O[C@@H](CC2=O)C3=CC=C(C=C3)O)O)OC)C
**8-[1-(4′-hydroxy-3′-methoxyphenyl)prop-2- en-1-yl]-(2S)-pinocembrin**	NCIRI-251	C=CC(c1ccc(O)c(OC)c1)c4c(O)cc(O)c3C(=O)C[C@H](c2ccccc2)Oc34
**8-deoxygartanin**	NCIRI-252	CC(=CCC1=C(C(=C2C(=C1O)C(=O)C3=C(O2)C(=CC=C3)O)CC=C(C)C)O)C
**8-octadecenoic acid methyl ester**	NCIRI-253	CCCCCCCCCC=CCCCCCCC(=O)OC
**8-prenyl-5,7-dihydroxy-3′-(3-hydroxy-3-methylbutyl)- 4′-methoxyflavanone**	NCIRI-254	CC(=CCC1=C2C(=C(C=C1O)O)C(=O)CC(O2)C3=CC(=C(C=C3)OC)CCC(C)(C)O)C

**9, 19- Cyclo- 9 beta- lanostane- 3 beta 25 diol**	NCIRI-255	CC(CCCC(C)(C)O)C1CCC2(C1(CCC34C2CCC5C3(C4)CCC(C5(C)C)O)C)C
**9,12-Octadecadienoic acid (Z,Z)**	NCIRI-256	CCCCCC=CCC=CCCCCCCCC(=O)O[Si](C)(C)C
**9-dodecenol**	NCIRI-257	CCC=CCCCCCCCCO
**9-hydroxy-6,7-dimethoxydalbergiquinol**	NCIRI-258	C=CC(c1ccccc1)c2cc(OC)c(OC)cc2O
**9-octadecen-1-ol-9z**	NCIRI-259	CCCCCCCCC=CCCCCCCCCO
**9-tricosene**	NCIRI-260	CCCCCCCCCCCCC/C=C/CCCCCCCC
**abietic acid**	NCIRI-261	CC(C)C1=CC2=CC[C@@H]3[C@@]([C@H]2CC1)(CCC[C@@]3(C)C(=O)O)C
**Acalycixeniolide K**	NCIRI-264	CC1=CCCC(=C)C2COC(=O)C(C2CC1)CCC=CCO
**Acetate, 3-cyclohexen-1-ol**	NCIRI-265	CC(=O)OC1CC=CCC1
**Acetophenone**	NCIRI-266	CC(=O)C1=CC=CC=C1
**acetoxymangiferonic acid**	NCIRI-267	[H][C@]4([C@H](C)CCC=C(OC(=O)O)C(=O)O)CC[C@@]5(C)[C@]2([H])CC[C@@]1([H])C(C)(C)C(=O)CC[C@@]13C[C@@]23CC[C@]45C
**Actinopyrone A**	NCIRI-268	CC=C(C)C(C(C)C=C(C)C=CCC(=CCC1=C(C(=O)C(=C(O1)OC)C)C)C)O
**Adenosine**	NCIRI-269	C1=NC(=C2C(=N1)N(C=N2)[C@H]3[C@@H]([C@@H]([C@H](O3)CO)O)O)N
**Adhyperforin**	NCIRI-270	CCC(C)C(=O)C12C(=O)C(=C(C(C1=O)(CC(C2(C)CCC=C(C)C)CC=C(C)C)CC=C(C)C)O)CC=C(C)C
**a-D-Xylopyranose**	NCIRI-271	C1C(C(C(C(O1)O)O)O)O
**agathadiol**	NCIRI-272	CC(=CCO)CCC1C(=C)CCC2C1(CCCC2(C)CO)C
**alfaxalone**	NCIRI-273	CC(=O)C1CCC2C1(CC(=O)C3C2CCC4C3(CCC(C4)O)C)C
**a-Linolenic acid**	NCIRI-274	CCC=CCC=CCC=CCCCCCCCC(=O)O
**Allo-Aromadendrene**	NCIRI-275	CC1CCC2C1C3C(C3(C)C)CCC2=C

**allyl alcohol**	NCIRI-276	C=CCO
**alpha amyryl hex-5-enoate**	NCIRI-277	[H][C@@]12CC(C)(C)CC[C@]1(C)CC[C@]5(C)C2=CC[C@]4([H])[C@@]3(C)CC[C@H](OC(=O)CCCC=C)C(C)(C)[C@]3([H])CC[C@]45C
**alpha cardinol**	NCIRI-278	CC1=C[C@H]2[C@@H](CC[C@@]([C@@H]2CC1)(C)O)C(C)C
**alpha copaene**	NCIRI-279	CC1=CCC2C3C1C2(CCC3C(C)C)C
**alpha selinene**	NCIRI-280	CC1=CCCC2(C1CC(CC2)C(=C)C)C
**Alpha-[p-bromophenyl]-O-amino cinnamic acid**	NCIRI-281	Nc1ccccc1C=C(C(=O)O)c2ccc(Br)cc2
**alpha-amyrin**	NCIRI-282	C[C@@H]1CC[C@@]2(CC[C@@]3(C(=CC[C@H]4[C@]3(CC[C@@H]5[C@@]4(CC[C@@H](C5(C)C)O)C)C)[C@@H]2[C@H]1C)C)C
**alpha-amyryl acetate**	NCIRI-283	[H][C@@]12CC(C)(C)CC[C@]1(C)CC[C@]5(C)C2=CC[C@]4([H])[C@@]3(C)CC[C@H](OC(C)=O)C(C)(C)[C@]3([H])CC[C@]45C
**alpha-amyryl pentanoate**	NCIRI-284	[H][C@@]12CC(C)(C)CC[C@]1(C)CC[C@]5(C)C2=CC[C@]4([H])[C@@]3(C)CC[C@H](OC(=O)CCCC)C(C)(C)[C@]3([H])CC[C@]45C
**alpha-Cadinene**	NCIRI-285	CC1=CC2C(CC1)C(=CCC2C(C)C)C
**alpha-tocopherol**	NCIRI-286	CC1=C(C2=C(CC[C@@](O2)(C)CCC[C@H](C)CCC[C@H](C)CCCC(C)C)C(=C1O)C)C
**alpha-Tocopherol Succinate**	NCIRI-287	CC1=C(C(=C(C2=C1OC(CC2)(C)CCCC(C)CCCC(C)CCCC(C)C)C)OC(=O)CCC(=O)O)C
**alpinetin**	NCIRI-288	COC1=CC(=CC2=C1C(=O)CC(O2)C3=CC=CC=C3)O
**alpinone**	NCIRI-289	COC1=CC(=C2C(=C1)OC(C(C2=O)O)C3=CC=CC=C3)O
**alpinone-3-acetate [20]**	NCIRI-290	CC(=O)O[C@@H]1[C@H](OC2=CC(=CC(=C2C1=O)O)OC)C3=CC=CC=C
**ambolic acid**	NCIRI-291	CC(CCC(=C)C(C)C(=O)O)C1CCC2(C1(CCC34C2CCC5C3(C4)CCC(C5(C)C)O)C)C
**ambonic acid**	NCIRI-292	CC(CCC(=C)C(C)C(=O)O)C1CCC2(C1(CCC34C2CCC5C3(C4)CCC(=O)C5(C)C)C)C

**Amphidinolide X**	NCIRI-293	CCCC1(CC2C(O1)CCC(=CC(C(OC(=O)CC(C=CC(=O)O2)C)CC(=O)C)C)C)C
**amyryl hexanoate**	NCIRI-294	[H][C@@]12CC(C)(C)CC[C@]1(C)CC[C@]5(C)C2=CC[C@]4([H])[C@@]3(C)CC[C@H](OC(=O)CCCCC)C(C)(C)[C@]3([H])CC[C@]45C
**anacardic acid**	NCIRI-295	CCCCCCCCCCCCCCC1=C(C(=CC=C1)O)C(=O)O
**aniline**	NCIRI-296	C1=CC=C(C=C1)N
**apigenin**	NCIRI-297	C1=CC(=CC=C1C2=CC(=O)C3=C(C=C(C=C3O2)O)O)O
**apigenin 7-O-apioglucoside**	NCIRI-298	O=c4cc(c1ccc(O)cc1)oc5cc(O[C@@H]2C[C@H](CO)[C@@H](O)[C@H](O)[C@H]2OC3OC[C@](O)(O)[C@@H]3O)cc(O)c45
**apigenin 7-O-apioglucoside (Apiin)**	NCIRI-299	C1C(C(C(O1)OC2C(C(C(OC2OC3=CC(=C4C(=C3)OC(=CC4=O)C5=CC=C(C=C5)O)O)CO)O)O)O)(CO)O
**Apigenin 7-O-glucoside (Apigetrin)**	NCIRI-300	C1=CC(=CC=C1C2=CC(=O)C3=C(C=C(C=C3O2)O[C@H]4[C@@H]([C@H]([C@@H]([C@H](O4)CO)O)O)O)O)O
**a-Pinene**	NCIRI-302	CC1=CCC2CC1C2(C)C
**Arisugacin E; 3-Ketone**	NCIRI-303	CC1(C(CCC2(C1(CCC3(C2CC4=C(O3)C=C(OC4=O)C5=CC=C(C=C5)OC)C)O)C)O)C
**artepilin C**	NCIRI-304	CC(=CCC1=CC(=CC(=C1O)CC=C(C)C)/C=C/C(=O)O)C
**ascorbic acid**	NCIRI-305	C([C@@H]([C@@H]1C(=C(C(=O)O1)O)O)O)O
**Baicalin**	NCIRI-307	C1=CC=C(C=C1)C2=CC(=O)C3=C(C(=C(C=C3O2)O[C@H]4[C@@H]([C@H]([C@@H]([C@H](O4)C(=O)O)O)O)O)O)O
**bavachromanol**	NCIRI-308	CC1(C(CC2=C(C=CC(=C2O1)C(=O)/C=C/C3=CC=C(C=C3)O)O)O)C
**Benzaldehyde**	NCIRI-310	C1=CC=C(C=C1)C=O
**Benzaldehyde, 4,6-dimethoxy-2,3-dimethyl-**	NCIRI-311	CC1=C(C(=C(C=C1OC)OC)C=O)C
**benzene acetic acid**	NCIRI-312	C1=CC=C(C=C1)CC(=O)O

**Benzene acetic acid, 4-hydroxy-3-methoxymethyl ester**	NCIRI-313	COC(=O)Cc1ccc(O)c(OC)c1
**Benzeneacetic acid, methyl ester**	NCIRI-314	COC(=O)CC1=CC=CC=C1
**benzenemethanol**	NCIRI-315	C1=CC=C(C=C1)CO
**Benzenepropanoic acid (hydrocinnamic acid)**	NCIRI-316	C1=CC=C(C=C1)CCC(=O)O
**Benzoic acid**	NCIRI-317	C1=CC=C(C=C1)C(=O)O
**benzyl (E)-ferulate**	NCIRI-318	COC1=C(C=CC(=C1)/C=C/C(=O)OCC2=CC=CC=C2)O
**benzyl (E)-isoferulate**	NCIRI-319	COC1=C(C=C(C=C1)/C=C/C(=O)OCC2=CC=CC=C2)O
**benzyl (E)-p-coumarate**	NCIRI-320	C1=CC=C(C=C1)COC(=O)/C=C/C2=CC=C(C=C2)O
**Benzyl benzoate**	NCIRI-321	C1=CC=C(C=C1)COC(=O)C2=CC=CC=C2
**benzyl caffeate**	NCIRI-322	C1=CC=C(C=C1)COC(=O)/C=C/C2=CC(=C(C=C2)O)O
**benzyl cinnamate**	NCIRI-323	C1=CC=C(C=C1)COC(=O)/C=C/C2=CC=CC=C2
**beta amyryl acetate**	NCIRI-324	[H][C@]12CC(C)(C)CC[C@]1(C)CC[C@]5(C)C2=CC[C@]4([H])[C@@]3(C)CC[C@H](OC(C)=O)C(C)(C)[C@]3([H])CC[C@]45C
**beta amyryl hex-5-enoate**	NCIRI-325	[H][C@]12CC(C)(C)CC[C@]1(C)CC[C@]5(C)C2=CC[C@]4([H])[C@@]3(C)CC[C@H](OC(=O)CCCC=C)C(C)(C)[C@]3([H])CC[C@]45C
**beta cadinene**	NCIRI-326	CC1=CC[C@@H]2[C@@H](C1)[C@@H](CC=C2C)C(C)C
**beta selinene**	NCIRI-327	CC(=C)C1CCC2(CCCC(=C)C2C1)C
**beta, Longipinene**	NCIRI-328	CC1(CCCC2C3C1C2C(=C)CC3)C
**beta-amyrine**	NCIRI-329	C[C@@]12CC[C@@]3(C(=CC[C@H]4[C@]3(CC[C@@H]5[C@@]4(CC[C@@H](C5(C)C)O)C)C)C1CC(CC2)(C)C)C
**beta-amyrone**	NCIRI-330	CC1(CCC2(CCC3(C(=CCC4C3(CCC5C4(CCC(=O)C5(C)C)C)C)C2C1)C)C)C

**beta-amyryl pentanoate**	NCIRI-331	[H][C@]12CC(C)(C)CC[C@]1(C)CC[C@]5(C)C2=CC[C@]4([H])[C@@]3(C)CC[C@H](OC(=O)CCCC)C(C)(C)[C@]3([H])CC[C@]45C
**beta-tocotrienol**	NCIRI-332	CC1=CC(=C(C2=C1O[C@](CC2)(C)CC/C=C(\C)/CC/C=C(\C)/CCC=C(C)C)C)O
**bicyclo(4,4,0)dec-1-ene**	NCIRI-334	C2=C1CCCCC1CCC2
**broussoflavonol**	NCIRI-335	CC(=CCC1=C(C(=C(C=C1C2=C(C(=O)C3=C(O2)C(=C(C=C3O)O)C(C)(C)C=C)O)O)O)CC=C(C)C)C
**Butanedioic acid (succinic acid)**	NCIRI-336	C(CC(=O)O)C(=O)O
**butin**	NCIRI-338	C1C(OC2=C(C1=O)C=CC(=C2)O)C3=CC(=C(C=C3)O)O
**cabralealactone**	NCIRI-339	C[C@@]12CC[C@@H]([C@H]1CC[C@H]3[C@]2(CC[C@@H]4[C@@]3(CCC(=O)C4(C)C)C)C)[C@@]5(CCC(=O)O5)C
**caffeic acid**	NCIRI-340	C1=CC(=C(C=C1/C=C/C(=O)O)O)O
**caffeic acid ethyl ester**	NCIRI-341	CCOC(=O)/C=C/C1=CC(=C(C=C1)O)O
**Caffeic acid isoprenyl ester**	NCIRI-342	CC(=C)C=COC(=O)/C=C/C1=CC(=C(C=C1)O)O
**calamenene**	NCIRI-344	C[C@H]1CC[C@H](C2=C1C=CC(=C2)C)C(C)C
**CAPE/phenethyl caffeate**	NCIRI-345	C1=CC=C(C=C1)CCOC(=O)/C=C/C2=CC(=C(C=C2)O)O
**cardanol**	NCIRI-348	C=CC/C=C\C/C=C\CCCCCCCC1=CC(=CC=C1)O
**cardanol (alkylphenol)**	NCIRI-349	C=CCC=CCC=CCCCCCCCC1=CC(=CC=C1)O
**Cardol (5-pentadecyl resorcinol)**	NCIRI-350	CCCCCCCCCCCCCCCC1=CC(=CC(=C1)O)O
**carvone**	NCIRI-351	CC1=CCC(CC1=O)C(=C)C
**Caryophyllene oxide**	NCIRI-352	CC1(CC2C1CCC3(C(O3)CCC2=C)C)C
**catechin**	NCIRI-353	C1[C@@H]([C@H](OC2=CC(=CC(=C21)O)O)C3=CC(=C(C=C3)O)O)O
**catechol**	NCIRI-354	C1=CC=C(C(=C1)O)O

**cathecin**	NCIRI-355	Oc3cc(O)c2C[C@H](O)[C@@H](c1ccc(O)c(O)c1)Oc2c3
**cearoin**	NCIRI-356	COC1=C(C=C(C(=C1)O)C(=O)C2=CC=CC=C2)O
**Cedrol**	NCIRI-357	C[C@@H]1CC[C@@H]2[C@]13CC[C@@]([C@H](C3)C2(C)C)(C)O
**chlorogenic acid**	NCIRI-358	C1[C@H]([C@H]([C@@H](C[C@@]1(C(=O)O)O)OC(=O)/C=C/C2=CC(=C(C=C2)O)O)O)O
**chrysin**	NCIRI-359	C1=CC=C(C=C1)C2=CC(=O)C3=C(C=C(C=C3O2)O)O
**chrysin-7-methyl ether**	NCIRI-360	COc3cc(O)c2c(=O)cc(c1ccccc1)oc2c3
**Chrysoeriol**	NCIRI-361	COC1=C(C=CC(=C1)C2=CC(=O)C3=C(C=C(C=C3O2)O)O)O
**chrysophanol**	NCIRI-362	CC1=CC2=C(C(=C1)O)C(=O)C3=C(C2=O)C=CC=C3O
**Cinnamic acid**	NCIRI-363	C1=CC=C(C=C1)C=CC(=O)O
**cinnamyl (E)-p-coumarate**	NCIRI-364	COC1=C(C=CC(=C1)/C=C/C(=O)OCCC2=CC=CC=C2)O
**Cinnamyl alcohol**	NCIRI-365	C1=CC=C(C=C1)C=CCO
**cinnamyl caffeate**	NCIRI-366	C1=CC=C(C=C1)/C=C/COC(=O)/C=C/C2=CC(=C(C=C2)O)O
**cinnamyl cinnamate**	NCIRI-367	C1=CC=C(C=C1)/C=C/COC(=O)/C=C/C2=CC=CC=C2
**cinnamylideneacetic acid**	NCIRI-368	C1=CC=C(C=C1)/C=C/C=C/C(=O)O
**Cis-lanceol**	NCIRI-370	CC1=CCC(CC1)C(=C)CCC=C(C)CO
**Citraconic anhydride**	NCIRI-371	CC1=CC(=O)OC1=O
**Citric acid**	NCIRI-372	C(C(=O)O)C(CC(=O)O)(C(=O)O)O
**clerodane diterpenoid I**	NCIRI-373	[H][C@]12CCC=C(C)[C@]1(C)CC[C@@H](CO)[C@]2(C)CCC(C)=CC(=O)O
**Cochinchinone A**	NCIRI-374	CC(=CCC/C(=C/CC1=C2C(=C(C(=C1O)CC=C(C)C)O)C(=O)C3=C(O2)C=CC(=C3)O)/C)C
**coniferyl aldehyde**	NCIRI-375	COC1=C(C=CC(=C1)/C=C/C=O)O

**coumaric acid cinnamyl ester**	NCIRI-376	C1=CC=C(C=C1)C=CCOC(=O)/C=C/C2=CC=C(C=C2)O
**cryptomeridiol**	NCIRI-377	CC12CCCC(C1CC(CC2)C(C)(C)O)(C)O
**curcumin**	NCIRI-378	COC1=C(C=CC(=C1)C=CC(=O)CC(=O)C=CC2=CC(=C(C=C2)O)OC)O
**cyclitol DERIVATIVE pinitol**	NCIRI-379	CO[C@H]1[C@@H](O)[C@H](O)[C@@H](O)[C@@H](O)[C@@H]1O
**cycloartenol**	NCIRI-380	C[C@H](CCC=C(C)C)[C@H]1CC[C@@]2([C@@]1(CC[C@]34[C@H]2CC[C@@H]5[C@]3(C4)CC[C@@H](C5(C)C)O)C)C
**cycloartenone**	NCIRI-381	C[C@H](CCC=C(C)C)[C@H]1CC[C@@]2([C@@]1(CC[C@]34[C@H]2CC[C@@H]5[C@]3(C4)CCC(=O)C5(C)C)C)C
**cycloartenyl acetate**	NCIRI-382	CC(CCC=C(C)C)C1CCC2(C1(CCC34C2CCC5C3(C4)CCC(C5(C)C)OC(=O)C)C)C
**cyclohexadecane**	NCIRI-383	C1CCCCCCCCCCCCCCC1
**cyclotetradecane**	NCIRI-384	C1CCCCCCCCCCCCC1
**daidzein**	NCIRI-385	C1=CC(=CC=C1C2=COC3=C(C2=O)C=CC(=C3)O)O
**Dalbergin**	NCIRI-386	COC1=C(C=C2C(=CC(=O)OC2=C1)C3=CC=CC=C3)O
**dammaradienol**	NCIRI-387	CC(=CCCC(=C)[C@H]1CC[C@@]2([C@@H]1CC[C@H]3[C@]2(CC[C@@H]4[C@@]3(CC[C@@H](C4(C)C)O)C)C)C)C
**dammaradienyl hex-5-enoate**	NCIRI-388	[H][C@]1(C(=C)CCC=C(C)C)CC[C@]4(C)[C@]1([H])CC[C@]3([H])[C@@]2(C)CC[C@H](OC(=O)CCCC=C)[C@](C)(C)[C@]2([H])CC[C@]34C
**dammaradienyl pentanoate**	NCIRI-389	[H][C@]1(C(=C)CCC=C(C)C)CC[C@]4(C)[C@]1([H])CC[C@]3([H])[C@@]2(C)CC[C@H](OC(=O)CCCC)[C@](C)(C)[C@]2([H])CC[C@]34C
**dammarane triterpene dipterocarpol**	NCIRI-390	[H][C@]34CCC2[C@@]1(C)CCC(=O)[C@](C)(C)C1CC[C@@]2(C)[C@]3(C)CCC4C(C)(O)CCC=C(C)C
**danthron**	NCIRI-391	C1=CC2=C(C(=C1)O)C(=O)C3=C(C2=O)C=CC=C3O
**daucoidin A**	NCIRI-392	C/C=C(/C)\C(=O)OC(C)(C)C1C(C2=C(O1)C=CC3=C2OC(=O)C=C3)O
**Decanal**	NCIRI-393	CCCCCCCCCC=O

**Decanoic acid (capric acid)**	NCIRI-394	CCCCCCCCCC(=O)O
**delta cadinene**	NCIRI-396	CC1=CC2C(CCC(=C2CC1)C)C(C)C
**Delta-9-tetra-hydrocannabinol acid**	NCIRI-397	CCCCCc1cc(O)c2c(c1)OC(C)(C)C3CCC(C)=CC23
**delta-cadinene**	NCIRI-398	CC1=C[C@H]2[C@@H](CCC(=C2CC1)C)C(C)C
**delta-tocotrienol**	NCIRI-399	CC1=CC(=CC2=C1O[C@](CC2)(C)CC/C=C(\C)/CC/C=C(\C)/CCC=C(C)C)O
**deoxypodophyllotoxin**	NCIRI-400	COC1=CC(=CC(=C1OC)OC)C2C3C(CC4=CC5=C(C=C24)OCO5)COC3=O
**d-fenchone**	NCIRI-401	CC1(C2CCC(C2)(C1=O)C)C
**D-Fructose (isomer 2)**	NCIRI-402	C1[C@H]([C@H]([C@@H](C(O1)(CO)O)O)O)O
**D-Galactose**	NCIRI-403	C(C1C(C(C(C(O1)O)O)O)O)O
**D-Glucitol**	NCIRI-404	C(C(C(C(C(CO)O)O)O)O)O
**diethyl ester (diethyl phthalate)**	NCIRI-405	CCOC(=O)C1=CC=CC=C1C(=O)OCC
**diethyl succinate**	NCIRI-406	CCOC(=O)CCC(=O)OCC
**Dihydro-.alpha.-terpineol**	NCIRI-408	CC1CCC(CC1)C(C)(C)O
**dihydroabietic acid**	NCIRI-409	CC(C)C1CCC2C(=C1)CCC3C2(CCCC3(C)C(=O)O)C
**dihydrobenzofuran**	NCIRI-410	c2ccc1OCCc1c2
**dimethyl sulfone**	NCIRI-411	CS(=O)(=O)C
**di-octyl-phthalate**	NCIRI-412	CCCCCCCCOC(=O)C1=CC=CC=C1C(=O)OCCCCCCCC
**dipterocarpol**	NCIRI-414	CC(=CCC[C@@](C)([C@H]1CC[C@@]2([C@@H]1CC[C@H]3[C@]2(CC[C@@H]4 [C@@]3(CCC(=O)C4(C)C)C)C)C)O)C
**diterpene propsiadin ((ent)-2-oxo-kaur-16-en-6,18-diol)**	NCIRI-415	C=C3C[C@]24C[C@H](O)[C@@]1(C)[C@@](C)(CO)CC(=O)CC1(C)[C@@]2(C)CC[C@H]3C4
**diterpene psiadin**	NCIRI-416	[H][C@H]2C[C@@]14CC(=C)[C@@H](CC[C@]1([H])C3(C)CC(=O)C[C@](CO)(CO)[C@]23[H])C4

**D-Limonene**	NCIRI-417	CC1=CC[C@@H](CC1)C(=C)C
**Docosane**	NCIRI-418	CCCCCCCCCCCCCCCCCCCCCC
**Docosanol**	NCIRI-419	CCCCCCCCCCCCCCCCCCCCCCO
**Dodecanoic acid (lauric acid)**	NCIRI-420	CCCCCCCCCCCC(=O)O
**Dodecasanyl hexadecanoate**	NCIRI-421	CCCCCCCCCCCCCCCCCCCCCCOC(=O)CCCCCCCCCCCCCCC
**dotriacontanol**	NCIRI-422	CCCCCCCCCCCCCCCCCCCCCCCCCCCCCCCCO
**E-11-hexadecen-1-ol**	NCIRI-423	CCCC/C=C/CCCCCCCCCCO
**E-Ethyl cinnamate**	NCIRI-424	CCOC(=O)/C=C/C1=CC=CC=C1
**Eicosane**	NCIRI-425	CCCCCCCCCCCCCCCCCCCC
**Eicosanol**	NCIRI-426	CCCCCCCCCCCCCCCCCCCCO
**Eicosyl hexadecanoate**	NCIRI-427	CCCCCCCCCCCCCCCCCCCCOC(=O)CCCCCCCCCCCCCCC
**ellagic acid**	NCIRI-428	C1=C2C3=C(C(=C1O)O)OC(=O)C4=CC(=C(C(=C43)OC2=O)O)O
**emodin**	NCIRI-429	CC1=CC2=C(C(=C1)O)C(=O)C3=C(C2=O)C=C(C=C3O)O
**ethyl acetate**	NCIRI-430	CCOC(=O)C
**ethyl benzoate**	NCIRI-431	CCOC(=O)C1=CC=CC=C1
**ethyl decanoate**	NCIRI-432	CCCCCCCCCC(=O)OCC
**Ethyl diazoacetate**	NCIRI-433	CCOC(=O)C=[N+]=[N-]
**ethyl palmitate**	NCIRI-434	CCCCCCCCCCCCCCCC(=O)OCC
**ethyl-3-phenyl propionate**	NCIRI-435	CCC1=CC(=CC=C1)OC(=O)CC
**Ethylvinylacetylene**	NCIRI-436	CCC#CC=C
**eugenol**	NCIRI-437	COC1=C(C=CC(=C1)CC=C)O

**ferruginol**	NCIRI-441	CC(C)C1=C(C=C2C(=C1)CC[C@@H]3[C@@]2(CCCC3(C)C)C)O
**ferulic acid**	NCIRI-442	COC1=C(C=CC(=C1)C=CC(=O)O)O
**ferulic acid 3',3'-dimethylallyl ester**	NCIRI-443	COc1cc(C=CC(=O)OCC=C(C)C)ccc1O
**ferulic acid benzyl ester**	NCIRI-444	COc2cc(C=CC(=O)Oc1ccccc1)ccc2O
**ferulic acid methyl ester**	NCIRI-445	COC1=C(C=CC(=C1)C=CC(=O)OC)O
**ferutinin**	NCIRI-446	CC1=CCC2(CCC(C2C(C1)OC(=O)C3=CC=C(C=C3)O)(C(C)C)O)C
**Fisetin**	NCIRI-447	C1=CC(=C(C=C1C2=C(C(=O)C3=C(O2)C=C(C=C3)O)O)O)O
**fisetinidol**	NCIRI-448	C1C(C(OC2=C1C=CC(=C2)O)C3=CC(=C(C=C3)O)O)O
**formononetin**	NCIRI-450	COC1=CC=C(C=C1)C2=COC3=C(C2=O)C=CC(=C3)O
**fumaric acid**	NCIRI-451	C(=C/C(=O)O)\C(=O)O
**furofurane lignane methyl pimoresinol**	NCIRI-452	[H][C@]23COC(c1ccc(OC)c(OC)c1)[C@@]2([H])COC3c4ccc(OC)c(OC)c4
**galandin**	NCIRI-453	O=C3c1c(O)cc(O)cc1OC(c2ccccc2)C3O
**galangin**	NCIRI-454	C1=CC=C(C=C1)C2=C(C(=O)C3=C(C=C(C=C3O2)O)O)O
**Galangin-5-methyl ether**	NCIRI-455	COC1=CC(=CC2=C1C(=O)C(=C(O2)C3=CC=CC=C3)O)O
**gallic acid**	NCIRI-456	C1=C(C=C(C(=C1O)O)O)C(=O)O
**gamma mangostin**	NCIRI-457	CC(=CCC1=C(C2=C(C=C1O)OC3=C(C2=O)C(=C(C(=C3)O)O)CC=C(C)C)O)C
**gamma-tocotrienol**	NCIRI-458	CC1=C(C=C2CC[C@@](OC2=C1C)(C)CC/C=C(\C)/CC/C=C(\C)/CCC=C(C)C)O
**garcinone B**	NCIRI-459	CC(=CCC1=C(C2=C(C=C1O)OC3=C(C2=O)C4=C(C(=C3)O)OC(C=C4)(C)C)O)C
**gartanin**	NCIRI-460	CC(=CCC1=C(C(=C2C(=C1O)C(=O)C3=C(C=CC(=C3O2)O)O)CC=C(C)C)O)C

**genistin**	NCIRI-462	C1=CC(=CC=C1C2=COC3=CC(=CC(=C3C2=O)O)O[C@H]4[C@@H]([C@H]([C@@H]([C@H](O4)CO)O)O)O)O
**genkwanin**	NCIRI-463	COC1=CC(=C2C(=C1)OC(=CC2=O)C3=CC=C(C=C3)O)O
**gentistic acid**	NCIRI-465	C1=CC(=C(C=C1O)C(=O)O)O
**geraniol**	NCIRI-466	CC(=CCCC(=CCO)C)C
**glyasperin A**	NCIRI-467	CC(=CCC1=C(C=CC(=C1)C2=C(C(=O)C3=C(O2)C=C(C(=C3O)CC=C(C)C)O)O)O)C
**Guaiol**	NCIRI-469	C[C@H]1CC[C@H](CC2=C1CC[C@@H]2C)C(C)(C)O
**heneicosane**	NCIRI-470	CCCCCCCCCCCCCCCCCCCCC
**Hentriacontane**	NCIRI-471	CCCCCCCCCCCCCCCCCCCCCCCCCCCCCCC
**hentriacontene**	NCIRI-472	CCCCCCCCCCCCCCCCCCCCCCCCCCCCCC=C
**heptacosane**	NCIRI-473	CCCCCCCCCCCCCCCCCCCCCCCCCCC
**heptacosene**	NCIRI-474	CCCCCCCCCCCCCCCCCCCCCCCCCC=C
**heptadecana**	NCIRI-475	CCCCCCCCCCCCCCCCC
**heptadecanoate**	NCIRI-476	CCCCCCCCCCCCCCCCC(=O)[O-]
**Heptadecanoic acid**	NCIRI-477	CCCCCCCCCCCCCCCCC(=O)O
**heptadecanoic acid, 15-methyl-ethyl ester**	NCIRI-478	CCC(C)CCCCCCCCCCCCCC(=O)OCC
**Heptatriacontane**	NCIRI-479	CCCCCCCCCCCCCCCCCCCCCCCCCCCCCCCCCCCCC
**heptylacetylene**	NCIRI-480	CCCCCCCC#C
**hesperetin**	NCIRI-482	COC1=C(C=C(C=C1)[C@@H]2CC(=O)C3=C(C=C(C=C3O2)O)O)O

**hesperidin**	NCIRI-483	C[C@H]1[C@@H]([C@H]([C@H]([C@@H](O1)OC[C@@H]2[C@H]([C@@H]([C@H]([C@@H](O2)OC3=CC(=C4C(=O)C[C@H](OC4=C3)C5=CC(=C(C=C5)OC)O)O)O)O)O)O)O)O
**hexacosane**	NCIRI-484	CCCCCCCCCCCCCCCCCCCCCCCCCC
**hexacosanol**	NCIRI-485	CCCCCCCCCCCCCCCCCCCCCCCCCCO
**Hexacosanyl hexadecanoate**	NCIRI-486	CCCCCCCCCCCCCCCCCCCCCCCCCCOC(=O)CCCCCCCCCCCCCCC
**hexacosene**	NCIRI-487	CCCCCCCCCCCCCCCCCCCCCCCCC=C
**hexadecane**	NCIRI-488	CCCCCCCCCCCCCCCC
**hexadecanoic acid**	NCIRI-489	CCCCCCCCCCCCCCCC(=O)O
**Hexanoic acid**	NCIRI-490	CCCCCC(=O)O
**Hexatriacontane**	NCIRI-491	CCCCCCCCCCCCCCCCCCCCCCCCCCCCCCCCCCCC
**HINOKIOL**	NCIRI-492	CC(C)C1=C(C=C2C(=C1)CCC3C2(CCC(C3(C)C)O)C)O
**HINOKIONE**	NCIRI-493	CC(C)C1=C(C=C2C(=C1)CCC3C2(CCC(=O)C3(C)C)C)O
**imbricatoloic acid**	NCIRI-497	CC(CCC1C(=C)CCC2C1(CCCC2(C)C(=O)O)C)CCO
**Inositol**	NCIRI-498	C1(C(C(C(C(C1O)O)O)O)O)O
**Isoaloeresin D**	NCIRI-501	CC1=CC(=C(C2=C1C(=O)C=C(O2)C[C@H](C)O)[C@H]3[C@@H]([C@H]([C@@H]([C@H](O3)CO)O)O)OC(=O)/C=C/C4=CC=C(C=C4)O)OC
**isocaryophyllene**	NCIRI-502	CC1=CCCC(=C)C2CC(C2CC1)(C)C
**isochlorogenic acid**	NCIRI-503	C1[C@H]([C@@H]([C@@H](C[C@@]1(C(=O)O)O)OC(=O)/C=C/C2=CC(=C(C=C2)O)O)O)O
**isochlorogenic acid C**	NCIRI-504	C1[C@H]([C@@H]([C@@H](C[C@]1(C(=O)O)O)OC(=O)/C=C/C2=CC(=C(C=C2)O)O)OC(=O)/C=C/C3=CC(=C(C=C3)O)O)O
**isocupressic acid**	NCIRI-505	CC(=CCO)CCC1C(=C)CCC2C1(CCCC2(C)C(=O)O)C

**isoferulic acid**	NCIRI-507	COC1=C(C=C(C=C1)C=CC(=O)O)O
**ISOLIQUIRITIGENIN**	NCIRI-508	C1=CC(=CC=C1/C=C/C(=O)C2=C(C=C(C=C2)O)O)O
**isonymphaeol-B**	NCIRI-509	CC(=CCCC(=CCC1=C(C(=CC(=C1)C2CC(=O)C3=C(C=C(C=C3O2)O)O)O)O)C)C
**isopent-3-enyl ferulate**	NCIRI-510	C=C(C)CCOC(=O)C=Cc1ccc(O)c(O)c1
**isopentyl caffeate**	NCIRI-511	CC(C)CCOC(=O)C=CC1=CC(=C(C=C1)O)O
**isopimaric acid**	NCIRI-512	C[C@@]1(CC[C@H]2C(=CC[C@@H]3[C@@]2(CCC[C@@]3(C)C(=O)O)C)C1)C=C
**isoprenyl-p- coumarate**	NCIRI-513	CC(C)=CCc1cc(C=CC(=O)O)ccc1O
**isorhamnetin**	NCIRI-514	COC1=C(C=CC(=C1)C2=C(C(=O)C3=C(C=C(C=C3O2)O)O)O)O
**izalpinin**	NCIRI-517	COC1=CC(=C2C(=C1)OC(=C(C2=O)O)C3=CC=CC=C3)O
**Jaceosidin**	NCIRI-518	COC1=C(C=CC(=C1)C2=CC(=O)C3=C(O2)C=C(C(=C3O)OC)O)O
**kaempferide**	NCIRI-519	COC1=CC=C(C=C1)C2=C(C(=O)C3=C(C=C(C=C3O2)O)O)O
**kaempferol**	NCIRI-520	C1=CC(=CC=C1C2=C(C(=O)C3=C(C=C(C=C3O2)O)O)O)O
**kaempferol 3-O-glucoside (Astragalin)**	NCIRI-521	C1=CC(=CC=C1C2=C(C(=O)C3=C(C=C(C=C3O2)O)O)O[C@H]4[C@@H]([C@H]([C@@H]([C@H](O4)CO)O)O)O)O
**Kaempferol-3-methyl ether**	NCIRI-522	COC1=C(OC2=CC(=CC(=C2C1=O)O)O)C3=CC=C(C=C3)O
**kaempferol-3-rutinoside**	NCIRI-524	C[C@H]1[C@@H]([C@H]([C@H]([C@@H](O1)OC[C@@H]2[C@H]([C@@H]([C@H]([C@@H](O2)OC3=C(OC4=CC(=CC(=C4C3=O)O)O)C5=CC=C(C=C5)O)O)O)O)O)O
**kurarinone**	NCIRI-527	CC(=CCC(CC1=C2C(=C(C=C1O)OC)C(=O)CC(O2)C3=C(C=C(C=C3)O)O)C(=C)C)C
**lanosta-8,24-dien-3-ol (3 beta)**	NCIRI-528	CC(CCC=C(C)C)[C@H]1CC[C@@]2([C@@]1(CCC3=C2CC[C@@H]4[C@@]3(CC[C@@H](C4(C)C)O)C)C)C

**lanosterol**	NCIRI-530	C[C@H](CCC=C(C)C)[C@H]1CC[C@@]2([C@@]1(CCC3=C2CC[C@@H]4[C@@]3(CC[C@@H](C4(C)C)O)C)C)C
**laserpitin**	NCIRI-531	C/C=C(/C)\C(=O)O[C@H]1C[C@@](C(=O)[C@H]([C@@]2([C@@H]1[C@@](CC2)(C(C)C)O)C)OC(=O)/C(=C\C)/C)(C)O
**lespeol**	NCIRI-532	CC(=CCCC1(C=CC2=C(O1)C=CC(=C2O)C(=O)/C=C/C3=CC=C(C=C3)O)C)C
**L-Gluconic acid**	NCIRI-533	C(C(C(C(C(C(=O)O)O)O)O)O)O
**Linalool**	NCIRI-535	CC(=CCCC(C)(C=C)O)C
**linoleic acid**	NCIRI-536	CCCCC/C=C\C/C=C\CCCCCCCC(=O)O
**Linoleic acid ethyl ether (ethyl linoleate)**	NCIRI-537	CCCCCC=CCC=CCCCCCCCC(=O)OCC
**liquiritigenin**	NCIRI-538	C1C(OC2=C(C1=O)C=CC(=C2)O)C3=CC=C(C=C3)O
**Lup-20(29)en-3one**	NCIRI-539	C=C(C)C4CCC5(C)CCC3(C)C(CCC2C1(C)CCC(=O)C(C)(C)C1CCC23C)C45
**lupenyl acetate**	NCIRI-540	CC(=C)[C@@H]1CC[C@]2([C@H]1[C@H]3CC[C@@H]4[C@]5(CC[C@@H](C([C@@H]5CC[C@]4([C@@]3(CC2)C)C)(C)C)OC(=O)C)C)C
**lupeol**	NCIRI-541	CC(=C)[C@@H]1CC[C@]2([C@H]1[C@H]3CC[C@@H]4[C@]5(CC[C@@H](C([C@@H]5CC[C@]4([C@@]3(CC2)C)C)(C)C)O)C)C
**luteolin**	NCIRI-542	C1=CC(=C(C=C1C2=CC(=O)C3=C(C=C(C=C3O2)O)O)O)O
**Luteolin 7-O-glucoside (Cynaroside)**	NCIRI-543	C1=CC(=C(C=C1C2=CC(=O)C3=C(C=C(C=C3O2)O[C@H]4[C@@H]([C@H]([C@@H]([C@H](O4)CO)O)O)O)O)O)O
**luteolin-5-methyl ether**	NCIRI-544	COC1=CC(=CC2=C1C(=O)C=C(O2)C3=CC(=C(C=C3)O)O)O
**mangiferolic acid**	NCIRI-545	C[C@H](CC/C=C(\C)/C(=O)O)[C@H]1CC[C@@]2([C@@]1(CC[C@]34[C@H]2CC[C@@H]5[C@]3(C4)CC[C@@H](C5(C)C)O)C)C)C
**mangiferonic acid**	NCIRI-546	C[C@H](CC/C=C(\C)/C(=O)O)[C@H]1CC[C@@]2([C@@]1(CC[C@]34[C@H]2CC[C@@H]5[C@]3(C4)CCC(=O)C5(C)C)C)C)C

**Methyl 3-(4'-hydroxyphenyl)prop-2-enoate**	NCIRI-548	COC(=O)C=CC1=CC=C(C=C1)O
**methyl caffeate**	NCIRI-549	COC(=O)C=CC1=CC(=C(C=C1)O)O
**Methyl docosanoate**	NCIRI-550	CCCCCCCCCCCCCCCCCCCCCC(=O)OC
**Methyl dodecannoate**	NCIRI-551	CCCCCCCCCCCC(=O)OC
**Methyl Dotriacontanoate**	NCIRI-552	CCCCCCCCCCCCCCCCCCCCCCCCCCCCCCCC(=O)OC
**Methyl eicosanoate**	NCIRI-553	CCCCCCCCCCCCCCCCCCCC(=O)OC
**METHYL HEXACOSANOATE**	NCIRI-554	CCCCCCCCCCCCCCCCCCCCCCCCCC(=O)OC
**Methyl hexadecanoate**	NCIRI-555	CCCCCCCCCCCCCCCC(=O)OC
**Methyl Octacosanoate**	NCIRI-556	CCCCCCCCCCCCCCCCCCCCCCCCCCCC(=O)OC
**Methyl octadecanoate**	NCIRI-557	CCCCCCCCCCCCCCCCCC(=O)OC
**Methyl octadecenoate**	NCIRI-558	CCCCCCCCCCCCCCC/C=C/C(=O)OC
**Methyl octadecenoate**	NCIRI-559	CCCCCCCCCCCCCCCC=CC(=O)OC
**Methyl tetracosanoate**	NCIRI-560	CCCCCCCCCCCCCCCCCCCCCCCC(=O)OC
**Methyl Triacontanoate**	NCIRI-561	CCCCCCCCCCCCCCCCCCCCCCCCCCCCCC(=O)OC
**methyleugenol**	NCIRI-562	COC1=C(C=C(C=C1)CC=C)OC
**M-heptadecyl ester**	NCIRI-563	CCCCCCCCCCCCCCCCCOC(=O)C1=CC=CC(=C1)C
**moretenol**	NCIRI-564	CC(=C)[C@@H]1CC[C@]2([C@H]1CC[C@@]3([C@@H]2CC[C@H]4[C@]3(CC[C@@H]5[C@@]4(CC[C@@H](C5(C)C)O)C)C)C)C
**Morin**	NCIRI-565	C1=CC(=C(C=C1O)O)C2=C(C(=O)C3=C(C=C(C=C3O2)O)O)O
**myricetin**	NCIRI-566	C1=C(C=C(C(=C1O)O)O)C2=C(C(=O)C3=C(C=C(C=C3O2)O)O)O
**Naphthalene,1,2,3,4,4a,5,6,8aoctahydro-4a,8-dimethyl-2-(1-methylethenyl)**	NCIRI-567	C=C(C)[C@H]2CC[C@]1(C)CCC=C(C)C1C2

**Naringenin**	NCIRI-568	C1C(OC2=CC(=CC(=C2C1=O)O)O)C3=CC=C(C=C3)O
**naringin**	NCIRI-569	CC1C(C(C(C(O1)OC2C(C(C(OC2OC3=CC(=C4C(=O)CC(OC4=C3)C5=CC=C(C=C5)O)O)CO)O)O)O)O)O
**Neobavaisoflavone**	NCIRI-570	CC(=CCC1=C(C=CC(=C1)C2=COC3=C(C2=O)C=CC(=C3)O)O)C
**Nonacosane**	NCIRI-572	CCCCCCCCCCCCCCCCCCCCCCCCCCCCC
**nonacosene**	NCIRI-573	CCCCCCCCCCCCCCCCCCCCCCCCCCCC=C
**nonadecane**	NCIRI-574	CCCCCCCCCCCCCCCCCCC
**nonanal**	NCIRI-575	CCCCCCCCC=O
**Nonanoic acid (pelargonic acid)**	NCIRI-576	CCCCCCCCC(=O)O
**nymphaeol A**	NCIRI-577	CC(=CCC/C(=C/CC1=C(C2=C(C=C1O)O[C@@H](CC2=O)C3=CC(=C(C=C3)O)O)O)/C)C
**nymphaeol B**	NCIRI-578	CC(=CCC/C(=C/CC1=C(C=CC(=C1O)O)[C@@H]2CC(=O)C3=C(C=C(C=C3O2)O)O)/C)C
**nymphaeol C**	NCIRI-579	CC(=CCC/C(=C/CC1=C(C=CC(=C1O)O)[C@@H]2CC(=O)C3=C(O2)C=C(C(=C3O)CC=C(C)C)O)/C)C
**nymphaeol-A**	NCIRI-580	CC(=CCCC(=CCC1=C(C2=C(C=C1O)OC(CC2=O)C3=CC(=C(C=C3)O)O)O)C)C
**nymphaeol-B**	NCIRI-581	CC(=CCCC(=CCC1=C(C=CC(=C1O)O)C2CC(=O)C3=C(C=C(C=C3O2)O)O)C)C
**nymphaeol-C**	NCIRI-582	CC(=CCCC(=CCC1=C(C=CC(=C1O)O)C2CC(=O)C3=C(O2)C=C(C(=C3O)CC=C(C)C)O)C)C
**obtusaquinol**	NCIRI-583	COC1=C(C=C(C(=C1)O)C(C=C)C2=CC=CC=C2)O
**ocotillone I**	NCIRI-584	C[C@@]12CC[C@@H]([C@H]1CC[C@H]3[C@]2(CC[C@@H]4[C@@]3(CCC(=O)C4(C)C)C)C)[C@]5(CC[C@H](O5)C(C)(C)O)C

**ocotillone II**	NCIRI-585	C[C@@]12CC[C@@H]([C@H]1CC[C@H]3[C@]2(CC[C@@H]4[C@@]3(CCC(=O)C4(C)C)C)C)[C@@]5(CC[C@@H](O5)C(C)(C)O)C
**Octacosane**	NCIRI-586	CCCCCCCCCCCCCCCCCCCCCCCCCCCC
**octacosanol**	NCIRI-587	CCCCCCCCCCCCCCCCCCCCCCCCCCCCO
**Octacosanyl hexadecanoate**	NCIRI-588	CCCCCCCCCCCCCCCCCCCCCCCCCCCCOC(=O)CCCCCCCCCCCCCCC
**octacosene**	NCIRI-589	CCCCCCCCCCCCCCCCCCCCCCCCCCC=C
**octadecane**	NCIRI-590	CCCCCCCCCCCCCCCCCC
**Octadecanoic acid (stearic acid)**	NCIRI-591	CCCCCCCCCCCCCCCCCC(=O)O
**Octadecanol**	NCIRI-592	CCCCCCCCCCCCCCCCCO
**octadecyl hexadecanoate**	NCIRI-593	CCCCCCCCCCCCCCCCCCOC(=O)CCCCCCCCCCCCCCC
**Octanal**	NCIRI-594	CCCCCCCC=O
**Octanoic acid**	NCIRI-595	CCCCCCCC(=O)O
**odoratin**	NCIRI-596	COC1=C(C=C(C=C1)C2=COC3=CC(=C(C=C3C2=O)OC)O)O
**olean-12-en-3-ol**	NCIRI-598	CC(=O)O[C@@H]1CCC2(C(C1(C)C)CCC3(C2CC=C4C3(CCC5(C4CC(CC5)(C)C)C)C)C)C
**oleana-9(11)-dien-3beta-ylacetate**	NCIRI-599	[H][C@]5(C)CC[C@]4(C)CC[C@]3(C)C(=CC=C2[C@@]1(C)CC[C@H](C(=O)OC)[C@@](C)(C)C1CC[C@]23C)C4[C@H]5C
**oleanic aldehydes**	NCIRI-600	[H][C@@H]5C4C3=CCC2[C@@]1(C)CC[C@H](O)[C@](C)(C)C1CC[C@@]2(C)[C@]3(C)CC[C@@]4(C=O)CC[C@@]5(C)C
**oleic acid**	NCIRI-601	CCCCCCCC/C=C\CCCCCCCC(=O)O
**Oleic acid, ethyl ester (ethyl oleate)**	NCIRI-602	CCCCCCCCC=CCCCCCCCC(=O)OCC
**O-methylpinoresinol**	NCIRI-604	COC1=C(C=C(C=C1)[C@H]2[C@@H]3CO[C@H]([C@@H]3CO2)C4=CC(=C(C=C4)O)OC)OC
**O-Xylene**	NCIRI-605	CC1=CC=CC=C1C

**Palmitelaidic acid**	NCIRI-607	CCCCCCC=CCCCCCCCC(=O)O
**p-coumaric acid**	NCIRI-608	C1=CC(=CC=C1/C=C/C(=O)O)O
**p-coumaric acid benzyl ester**	NCIRI-609	O=C(C=Cc1ccc(O)cc1)Oc2ccccc2
**penduletin**	NCIRI-611	COC1=C(C(=C2C(=C1)OC(=C(C2=O)OC)C3=CC=C(C=C3)O)O)OC
**pentacosane**	NCIRI-612	CCCCCCCCCCCCCCCCCCCCCCCCC
**pentacosene**	NCIRI-613	CCCCCCCCCCCCCCCCCCCCCCCC=C
**pentadecane**	NCIRI-614	CCCCCCCCCCCCCCC
**Pentatriacontane**	NCIRI-615	CCCCCCCCCCCCCCCCCCCCCCCCCCCCCCCCCCC
**Pentatriacontene**	NCIRI-616	CCCCCCCCCCCCCCCCCCCCCCCCCCCCCCCCCC=C
**penylacetaldehyde**	NCIRI-617	C1=CC=C(C=C1)CC=O
**Phenethyl alcohol**	NCIRI-618	C1=CC=C(C=C1)CCO
**phenethyl caffeate (CAPE)**	NCIRI-619	C1=CC=C(C=C1)CCOC(=O)C=CC2=CC(=C(C=C2)O)O
**phenethyl ferulate**	NCIRI-620	COc2cc(C=CC(=O)OCCc1ccccc1)ccc2O
**phenol,3-pentadecyl**	NCIRI-621	CCCCCCCCCCCCCCCC1=CC(=CC=C1)O
**phenyl ethanol**	NCIRI-622	CC(C1=CC=CC=C1)O
**pimaric acid**	NCIRI-623	C[C@]1(CC[C@H]2C(=C1)CC[C@@H]3[C@@]2(CCC[C@@]3(C)C(=O)O)C)C=C
**Pinobanksin**	NCIRI-624	C1=CC=C(C=C1)[C@@H]2[C@H](C(=O)C3=C(C=C(C=C3O2)O)O)O
**Pinobanksin 3 methyl ether**	NCIRI-625	COC3c1c(O)cc(O)cc1O[C@H](c2ccccc2)[C@H]3O
**pinobanksin 3-O-butanoate**	NCIRI-626	CCCC(=O)O[C@H]2C(=O)c1c(O)cc(O)cc1O[C@@H]2c3ccccc3
**pinobanksin 3-O-hexanoate**	NCIRI-627	CCCCCC(=O)O[C@H]2C(=O)c1c(O)cc(O)cc1O[C@@H]2c3ccccc3
**Pinobanksin 5,7- dimethyl ether**	NCIRI-628	COc3cc(OC)c2C(=O)[C@H](O)[C@@H](c1ccccc1)Oc2c3

**pinobanksin 5-methyl ether**	NCIRI-629	COC1=CC(=CC2=C1C(=O)[C@@H]([C@H](O2)C3=CC=CC=C3)O)O
**Pinobanksin-3-O- proprionate**	NCIRI-630	CCC(=O)O[C@@H]1[C@H](OC2=CC(=CC(=C2C1=O)O)O)C3=CC=CC=C3
**pinobanksin-3-o-(pentanoate or 2-methylbutyrate)**	NCIRI-631	CCCCC(=O)OC2C(=O)c1c(O)cc(O)cc1O[C@@H]2c3ccccc3
**Pinobanksin-3-O-acetate**	NCIRI-632	CC(=O)O[C@@H]1[C@H](OC2=CC(=CC(=C2C1=O)O)O)C3=CC=CC=C3
**Pinobanksin-3-O-butyrate**	NCIRI-633	CCCOC(=O)C2C(=O)c1c(O)cc(O)cc1OC2c3ccccc3
**pinobanksin-3-o-pentanoate**	NCIRI-634	CCCCC(=O)O[C@H]2C(=O)c1c(O)cc(O)cc1O[C@@H]2c3ccccc3
**Pinobanksin-3-O-propionate**	NCIRI-635	CCOC(=O)C2C(=O)c1c(O)cc(O)cc1OC2c3ccccc3
**pinobanksin-3-propionate**	NCIRI-636	CCC(=O)O[C@H]2C(=O)c1c(O)cc(O)cc1O[C@@H]2c3ccccc3
**Pinobanksin-5-methyl ether**	NCIRI-637	COC1=CC(=CC2=C1C(=O)C(C(O2)C3=CC=CC=C3)O)O
**Pinobanksin-5-methyl ether-3-O-acetate**	NCIRI-638	COc2cc(O)cc3O[C@H](c1ccccc1)[C@@H](OC(C)=O)C(=O)c23
**pinobanksin-7-methyl-ether**	NCIRI-639	COc3cc(O)c2C(=O)C(O)[C@@H](c1ccccc1)Oc2c3
**pinocembrin**	NCIRI-640	C1[C@H](OC2=CC(=CC(=C2C1=O)O)O)C3=CC=CC=C3
**pinocembrin-5-methyl ether**	NCIRI-641	COc2cc(O)cc3O[C@H](c1ccccc1)CC(=O)c23
**Pinostrobin**	NCIRI-642	COC1=CC(=C2C(=O)C[C@H](OC2=C1)C3=CC=CC=C3)O
**Pinostrobin chalcone**	NCIRI-643	COC1=CC(=C(C(=C1)O)C(=O)C=CC2=CC=CC=C2)O
**plathymenin**	NCIRI-644	C1C(OC2=CC(=C(C=C2C1=O)O)O)C3=CC(=C(C=C3)O)O
**p-mehtoxy-cinnamic acid cinnamyl ester**	NCIRI-645	COc2ccc(C=CC(=O)OCC=Cc1ccccc1)cc2
**p-Pentyloxynitrobenzene**	NCIRI-646	CCCCCOC1=CC=C(C=C1)[N+](=O)[O-]
**prenyl caffeate**	NCIRI-647	CC(=CCOC(=O)C=CC1=CC(=C(C=C1)O)O)C

**prokinawan**	NCIRI-648	CC(=CCC1=C(C2=C(C=C1O)OC(CC2=O)C3=CC(=C(C=C3)O)O)O)CCCC(C)(C)O
**propolin A**	NCIRI-649	CC(=CCC1=C(C=CC(=C1O)O)C2CC(=O)C3=C(C=C(C=C3O2)O)O)CCCC(C)(C)O
**propolin B**	NCIRI-650	CC(=CCC1=C(C(=CC(=C1)C2CC(=O)C3=C(C=C(C=C3O2)O)O)O)O)CCCC(C)(C)O
**propolin E**	NCIRI-651	CC(=CCc3cc([C@@H]2CC(=O)c1c(O)cc(O)cc1O2)ccc3O)CCCC(C)(C)O
**propolin F**	NCIRI-652	CC(=CCC/C(=C/CC1=C(C(=CC(=C1)[C@@H]2CC(=O)C3=C(C=C(C=C3O2)O)O)O)O)/C)C
**Propolin H**	NCIRI-653	CC(C)=CCCC(C)=CCc3cc([C@@H]2CC(=O)c1c(O)cc(O)cc1O2)ccc3O
**protocatheic acid**	NCIRI-655	O=C(O)c1ccc(O)c(O)c1
**psiadiarabin**	NCIRI-657	COC1=C(C(=C(C(=C1)C2=CC(=O)C3=C(C(=C(C=C3O2)OC)OC)O)OC)O)OC
**P-Xylene**	NCIRI-659	CC1=CC=C(C=C1)C
**pyrogallol**	NCIRI-660	C1=CC(=C(C(=C1)O)O)O
**quercetagetin-3,6-dimethylether**	NCIRI-661	COc3c(O)cc2oc(c1ccc(O)c(O)c1)c(OC)c(=O)c2c3O
**quercetin**	NCIRI-662	C1=CC(=C(C=C1C2=C(C(=O)C3=C(C=C(C=C3O2)O)O)O)O)O
**quercetin 3-O-galaktoside (Hyperoside)**	NCIRI-663	C1=CC(=C(C=C1C2=C(C(=O)C3=C(C=C(C=C3O2)O)O)O[C@H]4[C@@H]([C@H]([C@H]([C@H](O4)CO)O)O)O)O)O
**Quercetin -7 methyl ether**	NCIRI-664	COC1=CC(=C2C(=C1)OC(=C(C2=O)C3=CC(=C(C=C3)O)O)O)O
**quercetin dihydrate**	NCIRI-665	C1=CC(=C(C=C1C2=C(C(=O)C3=C(C=C(C=C3O2)O)O)O)O)O.O.O
**quercetin-5,7-dimethyl ether**	NCIRI-668	COc3cc(OC)c2c(=O)c(O)c(c1ccc(O)c(O)c1)oc2c3
**ResveratroL**	NCIRI-669	C1=CC(=CC=C1/C=C/C2=CC(=CC(=C2)O)O)O
**Retusapurpurin A**	NCIRI-670	COC1=CC2=C(CC(CO2)C3=CC4=C(C=C(OC4=CC3=O)C5=C(C=C(C=C5)O)OC)C6=CC=C(C=C6)O)C=C1

**Retusapurpurin B**	NCIRI-671	COC1=CC(=C(C=C1)C2=CC(=[O+]C3=CC(=C(C=C23)C4CC5=C(C=C(C=C5)OC)OC4)O)C6=CC=C(C=C6)O)O
**rhamnetin**	NCIRI-673	COC1=CC(=C2C(=C1)OC(=C(C2=O)O)C3=CC(=C(C=C3)O)O)O
**Rosifoliol**	NCIRI-676	CC1CCCC2(C1=CC(CC2)C(C)(C)O)C
**rosmarinic acid**	NCIRI-677	C1=CC(=C(C=C1C[C@H](C(=O)O)OC(=O)/C=C/C2=CC(=C(C=C2)O)O)O)O
**rutin**	NCIRI-679	C[C@H]1[C@@H]([C@H]([C@H]([C@@H](O1)OC[C@@H]2[C@H]([C@@H]([C@H]([C@@H](O2)OC3=C(OC4=CC(=CC(=C4C3=O)O)O)C5=CC(=C(C=C5)O)O)O)O)O)O)O)O
**Rutin hydrate**	NCIRI-680	C[C@H]1[C@@H]([C@H]([C@H]([C@@H](O1)OC[C@@H]2[C@H]([C@@H]([C@H]([C@@H](O2)OC3=C(OC4=CC(=CC(=C4C3=O)O)O)C5=CC(=C(C=C5)O)O)O)O)O)O)O)O.O
**sakuranetin**	NCIRI-681	COC1=CC(=C2C(=O)C[C@H](OC2=C1)C3=CC=C(C=C3)O)O
**salicylic acid**	NCIRI-682	C1=CC=C(C(=C1)C(=O)O)O
**salvigenin**	NCIRI-683	COC1=CC=C(C=C1)C2=CC(=O)C3=C(C(=C(C=C3O2)OC)OC)O
**sinapic acid**	NCIRI-686	COC1=CC(=CC(=C1O)OC)/C=C/C(=O)O
**Sorbose**	NCIRI-693	C1C(C(C(C(O1)(CO)O)O)O)O
**spathulenol**	NCIRI-694	CC1(C2C1C3C(CCC3(C)O)C(=C)CC2)C
**squalene**	NCIRI-695	CC(=CCCC(=CCCC(=CCCC=C(C)CCC=C(C)CCC=C(C)C)C)C)C
**Stearic acid, ethyl ester (ethyl stearate)**	NCIRI-697	CCCCCCCCCCCCCCCCCC(=O)OCC
**suberosin**	NCIRI-698	CC(=CCC1=C(C=C2C(=C1)C=CC(=O)O2)OC)C
**Sucrose**	NCIRI-699	C(C1C(C(C(C(O1)OC2(C(C(C(O2)CO)O)O)CO)O)O)O)O
**SUGIOL**	NCIRI-700	CC(C)C1=C(C=C2C(=C1)C(=O)CC3C2(CCCC3(C)C)C)O
**sulabiroins A**	NCIRI-701	[H][C@@]16COC[C@@]1([H])[C@H](c3cc(OC)c2OCOc2c3)c5c(cc4OCOc4c5OC)C6

**sulabiroins B**	NCIRI-702	[H][C@@]15COC[C@@]1([H])[C@H](c3cc(OC)c2OCOc2c3)c4c(cc(OC)c(OC)c4OC)C5
**syringic acid**	NCIRI-704	COC1=CC(=CC(=C1O)OC)C(=O)O
**taraxasterol**	NCIRI-706	CC1C2C3CCC4C5(CCC(C(C5CCC4(C3(CCC2(CCC1=C)C)C)C)(C)C)O)C
**TB1**	NCIRI-707	CC3(C)C(O)CCC4(C)Oc2c(C(=O)C=Cc1ccc(O)cc1)ccc(O)c2CC34
**TB2**	NCIRI-708	CC(C)=CCCCC3(C)Oc2c(C(=O)C=Cc1ccc(O)cc1)ccc(O)c2CC3O
**techtochrysin**	NCIRI-709	COC1=CC(=C2C(=C1)OC(=CC2=O)C3=CC=CC=C3)O
**teferin**	NCIRI-711	CC1=CCC2(CCC(C2C(C1)OC(=O)C3=CC(=C(C=C3)O)OC)(C(C)C)O)C
**tetracosane**	NCIRI-712	CCCCCCCCCCCCCCCCCCCCCCCC
**tetracosanoic acid**	NCIRI-713	CCCCCCCCCCCCCCCCCCCCCCCC(=O)O
**tetracosanol**	NCIRI-714	CCCCCCCCCCCCCCCCCCCCCCCCO
**Tetracosanyl hexadecanoate**	NCIRI-715	CCCCCCCCCCCCCCCCCCCCCCCCCCOC(=O)CCCCCCCCCCCCCCC
**Tetradecanoic acid (myristic acid)**	NCIRI-717	CCCCCCCCCCCCCC(=O)O
**tetradecanoic acid, ethyl ester**	NCIRI-718	CCCCCCCCCCCCCC(=O)OCC
**Tetratriacontane**	NCIRI-719	CCCCCCCCCCCCCCCCCCCCCCCCCCCCCCCCCC
**Tetratriacontene**	NCIRI-720	CCCCCCCCCCCCCCCCCCCCCCCCCCCCCCCCC=C
**thunbergol**	NCIRI-722	C/C/1=C\CCC(/C=C/C(CC/C(=C/CC1)/C)C(C)C)(C)O
**Tiglic-acid**	NCIRI-723	C/C=C(\C)/C(=O)O
**totarol**	NCIRI-725	CC(C)C1=C(C=CC2=C1CCC3C2(CCCC3(C)C)C)O
**totarolone**	NCIRI-726	CC(C)C1=C(C=CC2=C1CCC3C2(CCC(=O)C3(C)C)C)O
**trans 1-Phenyl-1-propen-3-ol 20-Hydroxyacetophenone**	NCIRI-727	O=C(C=CC(O)c1ccccc1)c2ccccc2O
**trans, trans-farnesol**	NCIRI-728	CC(=CCCC(=CCCC(=CCO[Si](C)(C)C)C)C)C

**trans-1,4-Butenedioic acid (fumaric acid)**	NCIRI-729	C(=CC(=O)O)C(=O)O
**trans-chalcon**	NCIRI-730	C1=CC=C(C=C1)/C=C/C(=O)C2=CC=CC=C2
**trans-cinnamic acid**	NCIRI-731	C1=CC=C(C=C1)/C=C/C(=O)O
**TRANS-Ferulic acid**	NCIRI-732	COC1=C(C=CC(=C1)/C=C/C(=O)O)O
**Triacontane**	NCIRI-733	CCCCCCCCCCCCCCCCCCCCCCCCCCCCCC
**triacontanol**	NCIRI-734	CCCCCCCCCCCCCCCCCCCCCCCCCCCCCCO
**Triacontene**	NCIRI-735	CCCCCCCCCCCCCCCCCCCCCCCCCCCCC=C
**tricosane**	NCIRI-737	CCCCCCCCCCCCCCCCCCCCCCC
**tridecane**	NCIRI-738	CCCCCCCCCCCCC
**tritricontene**	NCIRI-739	CCCCCCCCCCCCCCCCCCC=CCCCCCCCCCCCCC
**tschimganin**	NCIRI-740	CC1(C2CCC1(C(C2)OC(=O)C3=CC(=C(C=C3)O)OC)C)C
**tschimgin**	NCIRI-741	CC1(C2CCC1(C(C2)OC(=O)C3=CC=C(C=C3)O)C)C
**undecane**	NCIRI-742	CCCCCCCCCCC
**URS-9(11),12 DIEN-3-ONE**	NCIRI-743	[H][C@@]45C3=CC=C2[C@@]1(C)CCC(=O)C(C)(C)C1CC[C@@]2(C)[C@]3(C)CC[C@@]4(C)CC[C@@H](C)[C@@H]5C
**ursolic aldehydes**	NCIRI-744	[H][C@@]5(C)CC[C@]4(C=O)CC[C@]3(C)C(=CCC2[C@@]1(C)CC[C@H](O)[C@](C)(C)C1CC[C@]23C)C4[C@H]5C
**vanilin**	NCIRI-745	COC1=C(C=CC(=C1)C=O)O
**Vanillic acid**	NCIRI-746	COC1=C(C=CC(=C1)C(=O)O)O
**xanthoangelol**	NCIRI-750	CC(=CCC/C(=C/CC1=C(C=CC(=C1O)C(=O)/C=C/C2=CC=C(C=C2)O)O)/C)C
**xanthoangelol B**	NCIRI-751	CC(=C)C(CC/C(=C/CC1=C(C=CC(=C1O)C(=O)/C=C/C2=CC=C(C=C2)O)O)/C)O

**xanthoangelol F**	NCIRI-752	CC(=CCC/C(=C/CC1=C(C=CC(=C1O)C(=O)/C=C/C2=CC=C(C=C2)O)OC)/C)C
**xanthoangelol H**	NCIRI-753	CC1(C(CC2=C(C=CC(=C2O1)C(=O)/C=C/C3=CC=C(C=C3)O)OC)O)C
**Xanthoxyletin**	NCIRI-754	CC1(C=CC2=C(O1)C=C3C(=C2OC)C=CC(=O)O3)C
**α-Amyrin**	NCIRI-755	CC1CCC2(CCC3(C(=CCC4C3(CCC5C4(CCC(C5(C)C)O)C)C)C2C1C)C)C
**α-Amyrone**	NCIRI-756	CC1CCC2(CCC3(C(=CCC4C3(CCC5C4(CCC(=O)C5(C)C)C)C)C2C1C)C)C
**α-Bisabolol**	NCIRI-757	CC1=CCC(CC1)C(C)(CCC=C(C)C)O
**α-Cedrene**	NCIRI-758	CC1CCC2C13CC=C(C(C3)C2(C)C)C
**α-Curcumene**	NCIRI-759	CC1=CC=C(C=C1)C(C)CCC=C(C)C
**α-Eudesmol**	NCIRI-760	CC1=CCC[C@]2([C@H]1C[C@@H](CC2)C(C)(C)O)C
**α-Mangostin**	NCIRI-761	CC(=CCC1=C(C2=C(C=C1O)OC3=C(C2=O)C(=C(C(=C3)O)OC)CC=C(C)C)O)C
**β-Amyrin**	NCIRI-762	CC1(CCC2(CCC3(C(=CCC4C3(CCC5C4(CCC(C5(C)C)O)C)C)C2C1)C)C)C
**β-Eudesmol**	NCIRI-764	C[C@]12CCCC(=C)[C@@H]1C[C@@H](CC2)C(C)(C)O
**γ-Eudesmol**	NCIRI-765	CC1=C2C[C@@H](CC[C@]2(CCC1)C)C(C)(C)O
**δ-Cadinene**	NCIRI-766	CC1=C[C@@H]2[C@@H](CC1)C(=C)CC[C@H]2C(C)C

Table S3. Potential compounds of Asian propolis with proteins target and source countries

No	Compound	Country	Target
**1**	(+)-medicarpin	Nepal	AKR1B1(-9.8)
**2**	(+)-oxypeucedanin	South Korea	RBP4(-9.3)
**3**	(+)-vesticarpan	Nepal	AKR1B1 (-10.1), RBP4(-9.5)

**4**	(±)-(E)-4'-methoxy-4,2'-dihydroxy-3'-(2'',3''-dihydroxy-3''-methylbutyl)-chalcone	South Korea	HSD11B1(-9.0), GCK(-9.0), AKR1B1(-9.9), RXRA9-9.2)
**5**	(±)-(E)-4'-methoxy-4,3'',4''-trihydroxy-2'',2''-dimethyldihydropyrano-(2',3')-chalcone	South Korea	FFAR1(-9.4), HSD11B1(-9.5), PPARD(-9.0), GCK(-9.9), AKR1B1(-9.9), RXRA(-10.5)
**6**	(±)-(E,E)-4,2',4'-trihydroxy -3'-(5''-hydroxy-3'',7''-dimethyloct-2'',6''-dienyl)-chalcone	South korea	FFAR1(-9.4), PPARD(-9.0), AKR1B1(-10.0), PPARG(-9.7), RXRA(-10.6)
**7**	(22Z,24E)-3-oxocycloart-22,24-dien-26-oic acid	Myanmar	HSDB11B1(-10.8), PPARD(-9.4), DPP4(-10.3), MGAM(-9.4), AKR1B1(-9.0)
**8**	(23E)-27-nor-3beta-hydroxycycloart-23-en-25-one	Vietnam	AMY2A(-9.4), PPARD(-9.9)
**9**	(24E)-3beta-hydroxycycloart-24-en-26-al	Vietnam	HSDB11B1(-11,1), PPARD(-9.4), GCK(-9.6), AKR1B1(-9.1), PPARG(-9.7)
**10**	(24E)-3-oxo-27,28-dihydroxycycloart-24-en-26-oic acid	Myanmar	MGAM(-9.0)
**11**	(2R)-7,4′-Dihydroxy-5-methoxy-8-methylflavane	Vietnam	RBP4(-9.7)
**12**	(2R,3R)-pinobanksin 3-(2-methyl)-butyrate	Thailand	AKR1B1(-9.1)
**13**	(2R,3R)-pinobanksin 3-isobutyrate	Thailand	AKR1B1(-9.5)
**14**	(2S)-5,7,4′-trihydroxy-8,3′-diprenylflavanone	Myanmar	HSD11B1(-9.3), AMY2A(-9.3), DPP4(-9.4), PPARD(-9.5), GCK(-9.2), AKR1B1(-10.3), PPARG(-10.2), RXRA(-11.0)
**15**	(2S)-5,7,4′-trihydroxy-8-prenylflavanone	Myanmar	HSDB11B1(-9.2), PPARD(-9.6), AKR1B1(-9.5), RBP4(-10.8), RXRA(-9.3)

**16**	(2S)-5,7-dihydroxy-4′-methoxy-8,3-diprenylflavanone	Myanmar	HSD11B1(-9.4), DPP4(-9.7), PPARD(-9.2), GCK(-9.3), AKR1B1(-10.0) PPARG(-10.6)
**17**	(2S)-7- Methoxyflavanone	Nepal	AKR1B1(-9.0), RBP4(-9.2), RXRA(-9.4)
**18**	(2'S,3'R)-(+)-vaginidol	Sputh Korea	AKR1B1(-9.2)
**19**	(3S)-7,4′-Dihydroxy-5-methoxyhomoisoflavane	Vietnam	FFAR1(-9.2), AKR1B1(-9.7)
**20**	(5R,8S,9S,10R,13R,14S,17R,20R)-27-methoxycarbonyloxy- mangiferonic acid	Vietnam	HSD11B1(-9.8), DPP4(-9.0), PPARD(-11.0), AMY2A(-9.0)
**21**	(E)-1-(5-Bromothiophen-2-yl)-3-[4-(dimethylamino)phenyl]prop-2-en-1-one	South Korea	FFAR1(-9.1)
**22**	(E)-cinnamyl (E)-ferulate	Thailand	FFAR1(-9.3), AKR1B1(-10.3), RXRA(-9.5)
**23**	(E)-cinnamyl-(E)- cinnamylidenate	Thailand	FFAR1(-9.4), PPARD(-9.0), GCK(-9.5), AKR1B1(-10.2), PPARG(-9.5), RXRA(-9.6)
**24**	(E,E,E)-4,2',4'-trihydroxy-3'-(7''-hydroxy-3'',7''-dimethyloct-2'',5''-dienyl)-chalcone	South Korea	FFAR1(-9.9), HSDB11B1(-9.0), PPARD (-9.0), GCK(-9.1), AKR1B1(-10.5), PPARG(-9.6), RXRA(-9.7)
**25**	(RR)-(+)-3'-senecioylkhellactone	South Korea	HSD11B1(-9.5), PPARD(-10.0), GCK(-9.4), AKR1B1(-9.4), PPARG(-9.1), RXRA(-9.4)
**26**	(S)-(-)-selidin	South Korea	HSD11B1(-9.5), DPP4(-9.0), PPARD(-9.5), GCK(-9.5), AKR1B1(-9.6), RXRA(-9.3)
**27**	(S)-(+)-marmesin	South Korea	AKR1B1 (-9.1), RBP4(-9.5)
**28**	(SS)-(-)-isolaserpitin	South korea	HSDB11B1(-9.1), PPARD(-9.4), DPP4(-9.4), GCK(-9.3), AKR1B1(-9.4)
**29**	1,4- Dihydrophenanthrene	Iran	AKR1B1(-10.3)
**30**	10,11-Dihydroxydracaenone C	Vietnam	HSD11B1(-9.4)
**31**	10-hydroxybenzo[j]fluoranthene	Iran	HSD11B1(-10.0), AMY2A(-9.9), DPP4(-9.4), PPARD(-10.2), GCK(-9.7), AKR1B1(-11.0), INSR(-9.0), RBP4(-12.4), PPARG(-10.0), RXRA(-9.4)
**32**	13-epi-torulosal	Cyprus	GCK(-9.0)

**33**	1-methyl-4-azafluorenone	Turkey	AKR1B1(-9.0)
**34**	1-phenanthrenecarboxylic acid	Turkey	AKR1B1 (-10.1), RBP4(-9.8)
**35**	2(5H)-Furanone, 5,5-diphenyl	Turkey	FFAR1(-9.2), AKR1B1(-9.3), RBP4(-9.0)
**36**	2',6'-Dihydroxy-4'-methoxychalcone	Iran	AKR1B1(-9.2)
**37**	20,40,60-Trihydroxy chalcone (pinocembrin chalcone)	Iran	FFAR1(-9.4), AKR1B1(-9.0)
**38**	23-hydroxyisomangiferolic acid	Vietnam	HSD11B1(-9.7), AMY2A(-9.2), PPARD(-9.3)
**39**	23-hydroxymangiferolic acid	Vietnam	HSD11B1(-10.0), AMY2A(-9.1), PPARD(-9.6), AKRB1(-9.6)
**40**	23-hydroxymangiferonic acid	Vietnam	HSDB11B1(-11.9), PPARD(-10.2), AMY2A(-9.2), GCK(-9.3), PPARG(-9.5)
**41**	24-(Z)-3-oxolanosta-1,7,24-trien-26-oic acid	Jordan	FFAR1(-9.5), PDK2(-9.3), HSD11B1(-10.8), AMY2A(-10.9), DPP4(-9.8), PPARD(-10.4), MGAM(-9.8), AKR1B1(-9.6), INSR(-9.3), PPARG(-10.9), RXRA(-10.0)
**42**	24-Methylenecycloartenol	Saudi Arabia	FFAR1(-9.2), HSDB11B1(-11,1), DPP4(-9.2), PPARD (-9.9), GCK(-9.9), AKR1B1(-10.3), PPARG(-10.1)
**43**	27-acetoxymangiferolic acid	Vietnam	HSDB11B1(-10.4), PPARD(-10.0), AMY2A(-9.2), DPP4(-9.0), AKR1B1(-9.3)
**44**	27-hydroxymangiferolic acid	Vietnam	HSD11B1(-10.6), AMY2A(-9.1), PPARD(-9.2), GCK(-9.0), AKR1B1(-9.7), PPARG(-9.4)
**45**	27-hydroxymangiferoNic acid	Vietnam, Myanmar	HSD11B1(-11.3), AMY2A(-9.6), DPP4(-9.2), PPARD(-9.5), GCK(-9.1), PPARG(-9.0)
**46**	2H-2,4a-Ethanonaphthalene,1,3,4,5,6,7-hexahydro-2,5,5-trimethyl-	Jordan	FFAR1(-9.7)
**47**	2'-hydroxyformononetin	Nepal	AKR1B1(-9.5)
**48**	3,12-Oleandione	Iran	AMY2A(-9.0), DPP4(-9,6), PPARD(-9.7)
**49**	3,3-Diphenyl-cyclopropene	India	FFAR1(-10.2), AKR1B1(-9.0)
**50**	3',4'-dihydroxy-4-methoxydalbergione	Nepal	AKR1B1 (-9.3), RBP4(-9.2)

**51**	3,4-dimethoxycaffeic acid cinnamyl ester	South Korea	FFAR1(-9.0), AKR1B1(-10.3), PPARG(-9.0)
**52**	3′,8- diprenylnaringenin	Oman	HSD11B1(-9.7), AMY2A(-9.6), DPP4(-9.4), PPARD(-10.0), GCK(-9.4), AKR1B1(-10.7), PPARG(-9.6), RXRA(-11.0)
**53**	3′-Methoxydaidzin	China	HSD11B1(-9.8), PPARG(-9.7), PPARD(-9.3), AKRB1(-9.6)
**54**	3alpha,27-dihydroxycycloart-24-en-26-oic acid	Myanmar	HSD11B1(-10.2), PPARD(-10.2), AKRB1(-10.5), GCK(-9.4)
**55**	3B,23-dihydroxycycloart-24-en-26-oic acid	Myanmar	HSD11B1(-9.5), AMY2A(-9.0), PPARD(-9.3),
**56**	3B,27-dihydroxycycloart-24-en-26-oic acid	Myanmar	HSD11B1(-10,1), PPARD(-9.6), GCK(-9.4)
**57**	3'-Deoxysappanol	China	GCK(-9.4), AKR1B1(-9.4)
**58**	3'-geranylnaringenin	Japan	FFAR1(-9.9), HSD11B1(-10.8), DPP4(-9.7), PPARD(-10.3), GCK(-9.0), AKR1B1(-10.5), PPARA(-9.2), PPARG(-10.3), RXRA(-10.9)
**59**	3-Geranyloxy-1,7-dihydroxyxanthone	Vietnam	FFAR1(-9.9), HSD11B1(-10.2), PDK2(-9.0), PPARD(-9.9), GCK(-10.0), AKR1B1(-9.9), PPARG(-10.4), RXRA(-9.8)
**60**	3-O-acetyl ursolic acid	Thailand	HSD11B1(-9.3)
**61**	4,8,13-Cyclotetradecatriene-1,3-diol, 1,5,9-trimethyl-12-(1-methylethyl)-	Jordan	HSD11B1(-9.1), PPARD(-9.1)
**62**	4-hydroxyderricin	South Korea	FFAR1(-9.3), HSDB11B1(-9.1), PPARD (-19.1), GCK(-9.4), AKR1B1(-10.4), PPARG(-9.5), RXRA(-10.2)
**63**	4-hydroxymedicarpin	Nepal	AKR1B1 (-9.8), RBP4(-9.8)
**64**	4-methoxycinnamic acid cinnamyl ester	China	FFAR1(-9.3), AKR1B1(-10.2), GCK(-9.0), RXRA(-9.2)
**65**	4-methoxydalbergione	Nepal	AKR1B1(-9.2)
**66**	5,7-Dihyroxy-3-(iso)pentanoyloxyflavanone (pinobankin-3-isopentanoate)	Iran	PPARD(-9.0), AKR1B1(-9.5)

**67**	6,7-dihydroxyflavanon	Nepal	AKR1B1 (-9.0), RBP4(-9.3)
**68**	6-cinnamylchrysin	China	FFAR1(-9.8), HSD11B1(-10.6), DPP4(-9.2), PPARD(-10.2), AMY2A(-9.2), MGAM (-9.1), GCK(-10.4), AKR1B1(-10.5), PPARG(-11.2)
**69**	6-c-methyl quercetin	Iraq	AKR1B1(-9.1)
**70**	6-Dehydrogingerdione	Indonesia	AKR1B1(-9.0)
**71**	6-Epiangustifolin	Indonesia	PPARD(-9.8)
**72**	6-hydroxy-3-methoxy-6-(3-phenyl-2-propenyl)-2-cyclehexane-1-one	Nepal	FFAR1(-9.2), AKR1B1(-9.4)
**73**	7,4′-Dihydroxyhomoisoflavane	Vietnam	FFAR1(-9.0), AKR1B1(-10.1), RBP4(-9.2), RXRA(-9.2)
**74**	7-Geranyloxy-1,3-dihydroxyxanthone	Vietnam	FFAR1(-9.4), HSD11B1(-10.7), PPARD(-9.6), AKR1B1(-10.0),PPARG(-10.4), RXRA(-9.8)
**75**	7-hydroxyflavanone	Nepal	AKR1B1(-9.1), RBP4(-9.6), RXRA(-9.3)
**76**	7-O-methyl-8-prenylnaringenin	Oman	HSD11B1(-9.1), PPARD(-9.7), AKRB1(-9.1), RBP(-9.6)
**77**	8-[1-(4′-hydroxy-3′-methoxyphenyl)prop-2- en-1-yl]-(2S)-pinocembrin	Thailand	HSD11B1(-9.6),PPARD(-9.8), AKRB1(-10.1), GCK(-9.5)
**78**	8-deoxygartanin	Thailand	PPARD(-9.5), GCK(-9.4), AKR1B1(-9.1), PPARG(-9.1), RXRA(-9.1)
**79**	8-prenyl-5,7-dihydroxy-3′-(3-hydroxy-3-methylbutyl)- 4′-methoxyflavanone	Oman	HSD11B1(-9.5), AKR1B1(-9.4), PPARG(-10.2), AMY2A(-9.0)
**80**	9, 19- Cyclo- 9 beta- lanostane- 3 beta 25 diol	Malaysia	HSDB11B1(-11.1), PPARD(-9.5), AMY2A(-9.1), AKR1B1(-9.8), PPARG(-10.1)
**81**	abietic acid	Turkey	PPARD(-9.0), AKR1B1(-9.6)
**82**	Acalycixeniolide K	Indonesia	RBP4(-9.3), RXRA(-9.0)
**83**	acetoxymangiferonic acid	Vietnam	HSDB11B1(-10.0), PPARD(-10.6), AMY2A(-9.3), GCK(-9.3), PYGL(-9,6)
**84**	Actinopyrone A	Indonesia	FFAR1(-9.7), GCK(-9.3), PPARG(-9.2)
**85**	Adhyperforin	Indonesia	PPARD(-10.4), AKR1B1(-9.4)
**86**	alfaxalone	Iran	HSDB11B1(-10.1), PPARD(-9.0), AMY2A(-9.4), GCK(-9.5), PPARG(-9.1)
**87**	alpha amyryl hex-5-enoate	Yemen	HSD11B1(-10.3), AMY2A(-9.8), DPP4(-10.1)

**88**	Alpha-[p-bromophenyl]-O-amino cinnamic acid	India	AKR1B1(-9.4)
**89**	alpha-Amyrin	Yemen, Saudi Arabia, Vietnam	HSDB11B1(-10.5), PPARD(-9.3), AMY2A(-10.3), DPP4(-9.4), GCK(-9.1)
**90**	alpha-amyryl acetate	Yemen	HSD11B1(-9.5), AMY2A(-10.3) DPP4(-9.3)
**91**	alpha-amyryl pentanoate	Yemen	HSD11B1(-9.3), AMY2A(-9.4) DPP4(-9.8)
**92**	alpha-tocopherol	Turkey	HSD11B1(-9.1), PPARG(-9.5)
**93**	alpha-Tocopherol Succinate	Indonesia	HSD11B1(-9.3), PPARG(-9.5)
**94**	alpinetin	China	RBP4(-9.3)
**95**	alpinone	Jordan	RBP4(-9.3)
**96**	ambolic acid	Indonesia	HSDB11B1(-10.8), AMY2A(-9.6), DPP4(-9.1), PPARD (-10.0), MGAM (-9.0), GCK(-9.1), PPARG(-9.4)
**97**	ambonic acid	Indonesia	HSDB11B1(-11.3), AMY2A(-9.0), DPP4(-9.3), PPARD (-10.1), GCK(-9.7), AKR1B1(-9.5), PPARG(-9.8)
**98**	Amphidinolide X	Indonesia	PPARD(-11.8)
**99**	amyryl hexanoate	Yemen	HSD11B1(-10.2), AMY2A(-9.6) DPP4(-10.1)
**100**	apigenin	Malaysia, South korea, Uzbekistan, Lebanon, India, turkey, China, Iraq, Jordan	HSD11B1(-9.0), RXRA(-9.1), RBP4(-9.9), AKR1B1(-9.1)
**101**	apigenin 7-O-apioglucoside	China, Turkey	HSD11B1(-10.9), DPP4(-9.2), PPARD(-9.8), PPARG(-9.7)
**102**	Apigenin 7-O-glucoside (Apigetrin)	China	HSD11B1(-9.4), PPARG(-9.5), RXRA(-9.3), PPARD(-9.1)
**103**	Arisugacin E; 3-Ketone	Indonesia	HSD11B1(-11.6), AMY2A(-9.5),NR5A2(-9.0), DPP4(-9.6), PPARD(-9.5), PPARG(-9.0)
**104**	artepilin C	Uzbekistan	PPARD(-9.4), GCK(-9.0), AKR1B1(-9.5), RBP4(-9.3)
**105**	Baicalin	China	HSD11B1(-9.8), PPARG(-9.6), DPP4(-9.3), PPARD(-9.1)

**106**	bavachromanol	South Korea	FFAR1(-10.0), HSD11B1(-9.2), DPP4(-9.0), GCK(-9.8), AKR1B1(-10.8), RBP4(-9.3), PPARG(-9.3), RXRA (-10.7)
**107**	benzyl (E)-ferulate	Thailand	FFAR1(-9.4), AKR1B1(-9.6)
**108**	benzyl (E)-isoferulate	Thailand	FFAR1(-9.3), AKR1B1(-9.5), RBP4(-9.5)
**109**	benzyl (E)-p-coumarate	Thailand	FFAR1(-9.5), AKR1B1(-9.8), RBP4(-9.3), RXRA(-9.2)
**110**	benzyl caffeate	Turkey, Iraq, China	FFAR1(-9.5), AKR1B1(-9.8), RBP4(-9.6), RXRA(-9.0)
**111**	benzyl cinnamate	Turkey	FFAR1(-9.6), AKR1B1(-9.7), RBP4(-9.0)
**112**	beta amyryl acetate	Saudi Arabia, Yemen	AMY2A(-9.6), DPP4(-9.0)
**113**	beta amyryl hex-5-enoate	Yemen	HSD11B1(-9.5), DPP4(-9.3)
**114**	beta-Amyrin	Saudi Arabia, Yemen, Vietnam	HSD11B1(-9.9), AMY2A(-10.1), DPP4(-9.2), GCK(-9.2)
**115**	beta-amyrone	Yemen, Saudi Arabia	HSDB11B1(-10.3), PPARD(-9.3), AMY2A(-9.8), DPP4(-9.7), GCK(-9.8)
**116**	beta-amyryl pentanoate	Yemen	AMY2A(-9.4), PPARD(-9.0), RBP4(-10.2)
**117**	beta-tocotrienol	Vietnam	FFAR1(-9,9), HSD11B1(-9.8), DPP4(-9.0), PPARD(-9.1), GCK(-9.2), AKR1B1(-9.3), PPARG(-10.4), RXRA(-9.2)
**118**	broussoflavonol	Indonesia	RXRA(-9.1)
**119**	butin	Nepal	HSD11B1(-9.3), AKR1B1(-9,1), RBP4(-9.6), RXRA(-9.0)
**120**	cabralealactone	Thailand	HSD11B1(-9.3), AMY2A(-10.2) DPP4(-10.2)
**121**	Caffeic acid isoprenyl ester	China	FFAR1(-9.1)
**122**	calamenene	Turkey	RBP4(-9.0)
**123**	CAPE/phenethyl caffeate	Indonesia, India, China, Turkey, Thailand, South Korea, Uzbekistan, Iraq	FFAR1(-9.4), GCK(-9.2), AKR1B1(-10.3), RBP4(-9.8), RXRA(-9.1)
**124**	catechin	China	AKR1B1 (-9.0), RBP4(-9.0)

**125**	chrysin	Jordan, China, Thailand, Turkey, Iraq, Indonesia, Iran, lebanon, South Korea, Uzbekistan	AKR1B1(-9.0), RBP4(-9.6), RXRA(-9.2)
**126**	Chrysoeriol	China	HSD11B1(-9.2), AKR1B1(-9,1), RBP4(-9.7), RXRA(-9.2)
**127**	chrysophanol	Turkey	RBP4(-9.8)
**128**	cinnamyl (E)-p-coumarate	Thailand	FFAR1(-9.1), AKR1B1(-10.0), RXRA(-9.5)
**129**	cinnamyl caffeate	South Korea, Uzbekistan, iran, China,	FFAR1(-9.6), GCK(-9.7), AKR1B1(-10.3), RBP4(-9.6), PPARG(-9.0), RXRA(-9.5)
**130**	cinnamyl cinnamate	Turkey, China, Jordan, Thailand	FFAR1(-9.7), GCK(-9.5), AKR1B1(-10.3), PPARG(-9.1), RXRA(-9.4)
**131**	clerodane diterpenoid I	Iraq	RBP4(-9.4)
**132**	Cochinchinone A	Vietnam	HSDB11B1(-9.4), PPARD(-10.2), GCK(-10.0), AKR1B1(-11,2), PPARG(-10.5)
**133**	coumaric acid cinnamyl ester	South korea	FFAR1(-9.5), GCK(-9.7), AKR1B1(-10.3), RBP4(-9.3), RXRA(-9.7)
**134**	curcumin	India	FFAR1(-9.6), AKR1B1(-9.8), RXRA(-9.1)
**135**	cycloartenol	Indonesia, Vietnam, Saudi Arabia	HSDB11B1(-11.5), AMY2A(-9.0),DPP4(-9.0), PPARD (-9.8), GCK(-9.8), AKR1B1(-10.0), PPARG(-10.0)
**136**	cycloartenone	Vietnam	FFAR1(-9.3), HSD11B1(-11.5), PPARD(-10.1), GCK(-10.0),AKR1B1(-10.5), PPARG(-9.8)
**137**	cycloartenyl acetate	Saudi Arabia	HSD11B1(-10.6), AKR1B1(-10.2), AMY2A(-9.0), PPARG(-9.5)
**138**	daidzein	Turkey, lebanon	FFAR1(-9.9), AKR1B1(-9.2), RBP4(-9.9)
**139**	Dalbergin	Nepal	AKR1B1(-9.7)
**140**	dammaradienol	Yemen, Saudi Arabia	HSD11B1(-10)

**141**	dammaradienyl hex-5-enoate	Yemen	HSD11B1(-9.2), DPP4(-9.0)
**142**	dammaradienyl pentanoate	Yemen	HSD11B1(-9.3), PPARD(-9.4)
**143**	dammarane triterpene dipterocarpol	Thailand	FFAR1(-9.1), HSD11B1(-9.1), PPARD(-9.2), GCK(-9.0)
**144**	danthron	Turkey	RBP4(-9.0)
**145**	daucoidin A	South Korea	HSD11B1(-9.1), AKR1B1(-10.6), RBP4(-9.3), RXRA(-9.2)
**146**	Delta-9-tetra-hydrocannabinol acid	Iran	FFAR1(-9.2), HSD11B1(-9.0), PPARD(-9.2), GCK(-9.5), AKR1B1(-9.1)
**147**	delta-cadinene	Turkey, Malaysia	RBP4(-9.0)
**148**	delta-tocotrienol	Vietnam	FFAR1(-9.9), HSDB11B1(-9.8), DPP4(-9.2), PPARD (-9.4), AKR1B1(-9.2), PPARG(-10.4), RXRA(-9.0)
**149**	deoxypodophyllotoxin	Indonesia	PPARD(-9.1)
**150**	dihydroabietic acid	Turkey	RBP4(-10.2), RXRA(-9.1), PPARD(-9.1), GCK(-9.0)
**151**	dipterocarpol	Thailand	PPARD(-9.3), DPP4(-9.0), AKR1B1(-9.2)
**152**	diterpene propsiadin ((ent)-2-oxo-kaur-16-en-6,18-diol)	Saudi Arabia	PPARD(-9.2)
**153**	emodin	Turkey	RBP4(-9.5)
**154**	ferruginol	Turkey	AMY2A(-9.0), PPARD(-9.0), AKR1B1(-9.5)
**155**	ferulic acid benzyl ester	South Korea	FFAR1(-9.5), AKR1B1(-9.7)
**156**	ferutinin	Iran	AKR1B1 (-10.4), RBP4(-10.2)
**157**	Fisetin	China	HSD11B1(-9.4), RBP4(-9.2)
**158**	fisetinidol	Oman, Saudi Arabia	AKR1B1(-9.3)
**159**	formononetin	Nepal	FFAR1(-9.3), AKR1B1(-9.6), RBP4(-9.0)
**160**	galandin	China	RBP4(-9.2)
**161**	galangin	Iran, India, Lebanon, South Korea, Uzbekistan,	RBP4(-9.1)

		Jordan, China, Turkey, Iraq	
**162**	Galangin-5-methyl ether	China	RBP4(-9.5)
**163**	gamma mangostin	Thailand	HSD11B1(-9.6), DPP4(-9.0), PPARD(-9.6), GCK(-9.6), AKR1B1(-9.4), PPARG(-10.3)
**164**	gamma-tocotrienol	Vietnam	FFAR1(-9.5), HSD11B1(-9.7), DPP4(-9.1), PPARD(-9.8), GCK(-9.6), AKR1B1(-9.4), PPARG(-10.4), RXRA(-9.7)
**165**	garcinone B	Thailand	HSD11B1(-10.7), PTPN9(-9.4), DPP4(-9.4), PPARD(-9.1), GCK(-9.1), AKR1B1(-9.7), PPARA(-9.2), PPARG(-9.8)
**166**	gartanin	Thailand	HSDB11B1(-9.2), PPARD(-9.2), GCK(-9.5), AKR1B1(-10.4), PPARG(-9.1)
**167**	genistin	China	HSD11B1(-10.4), PPARG(-9.5)
**168**	genkwanin	Jordan	HSD11B1(-9.0), RBP(-9.6), RXRA(-9.1)
**169**	glyasperin A	Indonesia, Philipine	HSD11B1(-10.1), DPP4(-9.1), PPARD(-9.1), GCK(-9.2), AKR1B1(-9.7), PPARG(-10.5)
**170**	hesperetin	Iraq, China	FFAR1(-9.0), HSD11B1(-9.3), RBP4(-9.6)
**171**	hesperidin	Turkey	HSD11B1(-10.2), DPP4(-9.7, PPARD(-10.1), GCK(-9.4), PPARA(-9.1), INSR(-9.3), PYGL(-9.3), PPARG(-10.4)
**172**	HINOKIOL	Saudi Arabia	AKR1B1(-9.4), RBP4(-10.2), PPARG(-9.0)
**173**	HINOKIONE	Turkey, Saudi Arabia	HSD11B1(-9.0), AKR1B1(-9,3), RBP4(-9.7), PPARG(-9.1)
**174**	Isoaloeresin D	China	HSD11B1(-9.4), PPARD(-10.0)
**175**	isocupressic acid	Cyprus	RBP4(-9.1)
**176**	ISOLIQUIRITIGENIN	Nepal	FFAR1(-9.1), AKR1B1(-9.7), RXRA(-9.3)
**177**	isonymphaeol-B	Japan	FFAR1(-10.4), HSD11B1(-11.0), DPP4(-9.7), PPARD(-10.1), GCK(-9.5), AKR1B1(-10.3), PPARA(-9.5), PPARG(-10.1), RXRA(-9.0)
**178**	isopimaric acid	Turkey	RXRA(-9.5)
**179**	isorhamnetin	Indonesia, China, Turkey	HSD11B1(-9.1), RBP4(-9.5)
**180**	izalpinin	Thailand, China	RBP4(-9.3)

**181**	kaempferide	Iraq	RBP4(-9.5)
**182**	kaempferol	Indonesia, India, Iran, China, Turkey, Malaysia, South Korea, Uzbekistan, Lebanon, Iraq	HSD11B1(-9.2), RBP4(-9.5)
**183**	kaempferol 3-O-glucoside (Astragalin)	China	HSD11B1(-9.0), PPARG(-9.2), PPARD(-9.0)
**184**	kaempferol-3-rutinoside	Turkey	HSD11B1(-10.3), DPP4(-10.2), PPARD(-9.4), GCK(-9.1), INSR(-9,1), PPARG(-9.1)
**185**	kurarinone	Indonesia	HSDB11B1(-9.4), PPARD(-9.5), GCK(-9.9), AKR1B1(-9.6), PPARG(-9.1)
**186**	lanosterol	Saudi arabia, Vietnam, Malaysia	PPARD(-9.4)
**187**	laserpitin	South Korea	HSD11B1(-9.3), PPATD(-9.2)
**188**	lespeol	South Korea	FFAR1(-9.8), HSD11B1(-10.1), DPP4(-9.1), PPARD(-9.8), GCK(-10.1), AKR1B1(-10.4), PPARG(-10.5), RXRA(-12.0)
**189**	liquiritigenin	Nepal, Vietnam	AKR1B1(-9.1), RBP4(-9.6), RXRA(-9.3)
**190**	Lup-20(29)en-3one	Saudi Arabia	HSD11B1(-10.6), AKR1B1(-10.5), PPARG(-9.0), AMY2A(-9.6)
**191**	lupenyl acetate	Yemen, Saudi Arabia	PDK2(-9.0) AMY2A(-9.3), DPP4(-9.3), PPARD(-9.4), PPARG(-9.0)
**192**	lupeol	Saudi Arabia, Vietnam	HSD11B1(-9.4), AMY2A(-10.6), PPARD(-9.4), GCK(-9.0)
**193**	luteolin	Iraq, Malaysia, Iran, China, India, Turkey,	HSD11B1(-9.4), RBP4(-9.9), RXRA(-9.1), AKR1B1(-9.1)
**194**	Luteolin 7-O-glucoside (Cynaroside)	China, Turkey	HSDB11B1(-9.9), PPARD(-9.5), DPP4(-9.2), AKR1B1(-9.1), PPARG(-9.8)

**195**	luteolin-5-methyl ether	China	AKR1B1 (-9.2), RBP4(-9.4)
**196**	mangiferolic acid	Vietnam, Myanmar	HSD11B1(-10.9), AMY2A(-9.0), PPARD(-9.2), GCK(-9.9), PYGL(-9.1), PPARG(-10.3)
**197**	mangiferonic acid	Vietnam, Myanmar	FFAR1(-9.2), HSD11B1(-11.0), DPP4(-9.0), PPARD(-9.4), MGAM(-9.1), GCK(-10.2), AKR1B1(-10.4), PPARG(-9,9)
**198**	moretenol	Yemen, Saudi Arabia	HSDB11B1(-10.3), PPARD(-9.1), AMY2A(-9.5), DPP4(-10.3), PPARG(-10.8)
**199**	Morin	China	HSD11B1(-9.3), AKR1B1(-9.1), RBP4(-9.4)
**200**	myricetin	China	HSD11B1(-9.6), RBP4(-9.6)
**201**	Naringenin	Jordan, Turkey, Iran, Iraq, China,	RBP4(-9.7)
**202**	naringin	Bangladesh, India	PDK2(-9.1), HSD11B1(-9.1), AMY2A(-9.2), PTPN9(-9.0), DPP4(-10.0), PPARD(-9.9), GCK(-9.2), PPARG(-10.2)
**203**	Neobavaisoflavone	China	FFAR1(-9.8), HSDB11B1(-9.9), PPARD (-9.3), GCK(-9.9), RBP4(-10.4), PPARG(-9.4), PPARG(-9.1)
**204**	nymphaeol C	Japan	HSDB11B1(-9.7), DPP4(-9.2), PPARD (-10.7), MGAM(-9.4), GCK(-9.3), AKR1B1(-10.8), PPARG(-10.8)
**205**	nymphaeol-A	Japan	FFAR1(-9.3), HSD11B1(-9.1), AMY2A(-9.1), DPP4(-9.3), GCK(-9.3), PPARG(-10.3)
**206**	nymphaeol-B	Japan	HSD11B1(-9.7), DPP4(-9.1), PPARD(-10,1), GCK(-9.5), AKR1B1(-10.3), RXRA(-10.4)
**207**	ocotillone I	Thailand	AMY2A(-9.0)
**208**	ocotillone II	Thailand	AMY2A(-9.0), PPARD(-9,1)
**209**	olean-12-en-3-ol	Turkey	HSD11B1(-9.6), AMY2A(-9.8)
**210**	oleana-9(11)-dien-3beta-ylacetate	Saudi Arabia	AMY2A(-9.4), DPP4(-9.2), PPARD(-9.3)
**211**	oleanic aldehydes	Thailand	AMY2A(-9.8), GCK(-9.0)
**212**	O-methylpinoresinol	Thailand	RXRA(-9.3)
**213**	p-coumaric acid benzyl ester	China	FFAR1(-9.6), AKR1B1(-9.8), RXRA(-9.0)
**214**	phenethyl ferulate	Thailand	FFAR1(-9.3), AKR1B1(-9.8), RXRA(-9.3)

**215**	Phenylethyl trans-4-coumarate	Iran	FFAR1(-9.5), GCK(-9.1), AKR1B1(-10.0), RBP4(-9.3), RXRA(-9.3)
**216**	pimaric acid	Turkey	PPARD(-9.9)
**217**	Pinobanksin	Turkey, China, Iran, South Korea, uzbekistan, Iraq	RBP4(-9.0)
**218**	pinobanksin 3-O-butenoate	Turkey	AKR1B1(-9.3)
**219**	pinobanksin 3-O-hexanoate	Turkey	PPARD(-9.5), AKR1B1(-9.0)
**220**	Pinobanksin 5,7- dimethyl ether	Iran	RBP4(-9.5)
**221**	Pinobanksin-3-O- proprionate	Iran	AKR1B1(-9.5)
**222**	Pinobanksin-3-O-acetate	China	AKR1B1 (-9.3), RBP4(-9.3)
**223**	Pinobanksin-3-O-butyrate	China	AKR1B1(-9.0)
**224**	pinobanksin-3-o-pentanoate	Iran	PPARD(-9.2), AKR1B1(-9.5)
**225**	Pinobanksin-5-methyl ether	China, South Korea, Uzbekistan	RBP4(-9.4)
**226**	Pinobanksin-5-methyl ether-3-O-acetate	China	AKR1B1(-9.0)
**227**	pinocembrin	India, Lebanon, SouthKorea, Uzbekistan, Jordan, Iraq, Iran, China, Turkey, Nepal, Thailand	RBP4(-9.6)
**228**	pinocembrin-5-methyl ether	China	RBP4(-9.4)
**229**	Pinostrobin	Iran, Thailand, Turkey, Iraq, China	RBP4(-9.6)
**230**	Pinostrobin chalcone	Iran	AKR1B1(-9.5)

**231**	plathymenin	Nepal	HSD11B1(-9.7), AKR1B1(-9.1), RBP4(-9.2)
**232**	p-mehtoxy-cinnamic acid cinnamyl ester	China	FFAR1(-9.4), GCK(-9.1), AKR1B1(-10.3)
**233**	prenyl caffeate	Iraq, Jordan, Turkey	FFAR1(-9.0)
**234**	prokinawan	Japan	HSD11B1(-10.2), GCK(-9.4)
**235**	propolin A	Japan, Philipine	HSD11B1(-9.1), PPARD(-9.3) GCK(-9.2), AKR1B1(-9.7), PPARG(-9.3), RXRA(-9.4)
**236**	propolin B	Japan	FFAR1(-9.4), HSDB11B1(-9.9), DPP4(-9.1), PPARD (-9.3), GCK(-9.4), AKR1B1(-9.0), PPARG(-9.4)
**237**	propolin E	Philipine, Japan	FFAR1(-9.3), HSD11B1(-10.3), PPARD(-9.3), GCK(-9.5), PPARG(-9.6), RXRA(-10.0)
**238**	propolin F	Taiwan	FFAR1(-9.6), HSD11B1(-11.1), PPARD(-9.9), GCK(-9.1), AKR1B1(-9.7), PPARG(-10.2)
**239**	Propolin H	Philipine	FFAR1(-9.5), HSD11B1(-10.7), DPP4(-9.1), PPARD(-10.1), GCK(-9.7), AKR1B1(-10.3), PPARA(-9.0), PPARG(-10.0), RXRA(-10.0)
**240**	quercetin	Iraq, Bangladesh, Indonesia, India, China, Iran, Turkey, Malaysia, Lebanon, Uzbekistan	HSD11B1(-9.8), RBP4 (-9.6)
**241**	Quercetin -7 methyl ether	Iran	HSD11B1(-9.0),DPP4(-9.1)
**242**	ResveratroL	China, Turkey	FFAR1(-9.6), AKR1B1(-9.1)
**243**	Retusapurpurin A	Nepal	PDK2(-9.3), HSD11B1(-9.6), AMY2A(-10.7), NR5A(-9.7), DPP4(-10.9), PPARD(-10.6), GCK(-10.1), AKR1B1(-10.0), PPARA(-9.0), INSR(-9.6), PPARG(-12.0)
**244**	Retusapurpurin B	Nepal	AMY2A(-9.8), NR5A2(-9.3), DPP4(-10.4), PPARD (-10.9), AKR1B1(-10.4), PPARA(-9.4), PPARG(-11.7)

**245**	rhamnetin	China	HSD11B1(-9.4)
**246**	rosmarinic acid	China, Turkey	AKR1B1 (-10.5), PPARG(-9.1)
**247**	rutin	Turkey, Lebanon, China	HSDB11B1(-10.3), PPARD(-9.6), DPP4(-9.9), PPARA(-9.2), PPARG(-9.1)
**248**	sakuranetin	Iraq, Turkey, China	RBP4(-9.6)
**249**	salvigenin	Turkey	AKR1B1(-9.1)
**250**	squalene	Saudi Arabia, Philipine	PPARD(-9.5), AKR1B1(-9.4), RXRA(-9.2)
**251**	suberosin	Iran	AKR1B1 (-9.5), RBP4(-9.8)
**252**	SUGIOL	Saudi Arabia	HSD11B1(-9.4)
**253**	sulabiroins A	Indonesia	PPARD(-9.0), AKR1B1(-9.4)
**254**	taraxasterol	Saudi Arabia	HSD11B1(-10.1), INSR(-10.1), AMY2A(-9.7)
**255**	TB1	South Korea	FFAR1(-10.4), HSD11B1(-9.6), AMY2A(-9.6), DPP4(-9.1), PPARD(-10.2), AKR1B1(-10.5)
**256**	TB2	South Korea	FFAR1(-9.6), HSD11B1(-9.4), DPP4(-9.4), PPARD(-9.3), GCK(-10.0), AKR1B1(-11.3), PPARG(-9.4), RXRA(-9.9)
**257**	techtochrysin	China, Iraq, South Korea, Uzbekistan, Thailand	RBP4(-9.5)
**258**	teferin	Iran	PPARD(-9.1), AKR1B1(-9.0), RBP4(-10.2)
**259**	thunbergol	Turkey	PPARD(-9.2)
**260**	totarol	Saudi Arabia, Cyprus	HSD11B1(-9.0), GCK(-9.0), AKR1B1(-9.3), RBP4(-9.1)
**261**	totarolone	Turkey	GCK(-9.3)
**262**	trans 1-Phenyl-1-propen-3-ol 20-Hydroxyacetophenone	Iran	FFAR1(-9.9), AKR1B1(-9.5)
**263**	trans-chalcon	Turkey	FFAR1(-9.7), AKR1B1(-9.4)

**264**	tschimganin	Iran	AKR1B1 (-9.3), RBP4(-9.6)
**265**	tschimgin	Iran	AKR1B1 (-9.2), RBP4(-9.6)
**266**	URS-9(11),12 DIEN-3-ONE	Saudi Arabia	HSD11B1(-11.0), AMY2A(-10.6), DPP4(-9.2)
**267**	ursolic aldehydes	Thailand	HSD11B1(-10.3), AMY2A(-10.4), GCK(-9.0)
**268**	xanthoangelol	South Korea	FFAR1(-9.4), HSD11B1(-9.6), GCK(-9.5), AKR1B1(-10.0), PPARG(-9.8), RXRA (-11,0)
**269**	xanthoangelol B	South Korea	FFAR1(-9.1), HSD11B1(-9.4), GCK(-9.6), AKR1B1(-9.7), PPARG(-9.7), RXRA(-10.1)
**270**	xanthoangelol F	South Korea	FFAR1(-9.2), HSDB11B1(-9.5), PPARD (-9.0), GCK(-9.4), AKR1B1(-11.0), PPARG(-10.0), RXRA(-10.8)
**271**	xanthoangelol H	South Korea	FFAR1(-9.7), HSD11B1(-9.5), DPP4(-9.2), GCK(-9.9), AKR1B1(-10.8), RXRA(-10.6)
**272**	α-Amyrone	Saudi Arabia, Yemen	HSD11B1(-10.7), AMY2A(-10.6), DPP4(-9.7), PPARD(-9.7), GCK(-9.8), PYGL(-9.1)
**273**	α-Mangostin	Thailand, Vietnam	HSD11B1(-9.6), DPP4(-9.2), PPARD(-9.2), GCK(-9.3), AKR1B1(-9.4), PPARG(-10.6)
**274**	5,7-Dihydroxy-4'-methoxy-8-C-prenylflavanone	Myanmar	PPARD(-9.8), AKR1B1(-9.5), RBP4(-10.1)
**275**	isochlorogenic acid	China	AKR1B1(-9.1)

References

1. Tanvir, E.M.; Hasan, M.A.; Nayan, S.I.; Islam, T.; Ahmed, T.; Hossen, M.S.; Perveen, R.; Rahman, S.; Afroz, R.; Afroz, R.; et al. Ameliorative effects of ethanolic constituents of Bangladeshi propolis against tetracycline-induced hepatic and renal toxicity in rats. *J. Food Biochem.* **2019**, *43*, 1–11, doi:10.1111/jfbc.12958.

2. Ristivojević, P.; Stević, T.; Starović, M.; Pavlović, S.; Özcan, M.M.; Berić, T.; Dimkić, I. Phenolic composition and biological activities of geographically different type of propolis and black cottonwood resins against oral streptococci, vaginal microbiota and phytopathogenic Fusarium species. *J. Appl. Microbiol.* **2020**, doi:10.1111/jam.14633.

3. Wang, T.; Liu, Q.; Wang, M.; Zhang, L. Metabolomics Reveals Discrimination of Chinese Propolis from Different Climatic Regions. **2020**, 3–8.

4. Yuan, M.; Yuan, X.J.; Pineda, M.; Liang, Z.Y.; He, J.; Sun, S.W.; Pan, T.L.; Li, K.P. A comparative study between Chinese propolis and Brazilian green propolis: Metabolite profile and bioactivity. *Food Funct.* **2020**, *11*, 2368–2379, doi:10.1039/c9fo02051a.

5. Chi, Y.; Luo, L.; Cui, M.; Hao, Y.; Liu, T.; Huang, X.; Guo, X. Chemical Composition and Antioxidant Activity of Essential Oil of Chinese Propolis. *Chem. Biodivers.* **2020**, *17*, doi:10.1002/cbdv.201900489.

6. Xu, X.; Pu, R.; Li, Y.; Wu, Z.; Li, C.; Miao, X.; Yang, W. Chemical compositions of propolis from China and the United States and their antimicrobial activities against penicillium notatum. *Molecules* **2019**, *24*, doi:10.3390/molecules24193576.

7. Jiang, X.; Tian, J.; Zheng, Y.; Zhang, Y.; Wu, Y.; Zhang, C.; Zheng, H.; Hu, F. A new propolis type from changbai mountains in north-east china: Chemical composition, botanical origin and biological activity. *Molecules* **2019**, *24*, doi:10.3390/molecules24071369.

8. Narter, F.; Diren, A.; Kafkaslı, A.; Eronat, A.P.; Seyhan, M.F.; Yılmaz-Aydoğan, H.; Sarıkaya, S.; Hatipoğlu, S.D.; Sarıca, K.; Ozturk, O. Anatolian propolis prevents oxalate kidney stones: Dramatic reduction of crystal deposition in ethylene-glycol-induced rat model. *Rec. Nat. Prod.* **2018**, *12*, 445–459, doi:10.25135/rnp.48.17.11.075.

9. Sun, L.; Liao, L.; Wang, B. Potential antinociceptive effects of Chinese propolis and identification on its active compounds. *J. Immunol. Res.* **2018**, *2018*, 1–6, doi:10.1155/2018/5429543.

10. Sun, L.; Wang, K.; Xu, X.; Ge, M.; Chen, Y.; Hu, F. Potential Protective Effects of Bioactive Constituents from Chinese Propolis against Acute Oxidative Stress Induced by Hydrogen Peroxide in Cardiac H9c2 Cells. *Evidence-based Complement. Altern. Med.* **2017**, *2017*, doi:10.1155/2017/7074147.

11. Chang, H.; Yuan, W.; Wu, H.; Yin, X.; Xuan, H. Bioactive components and mechanisms of Chinese poplar propolis alleviates oxidized low-density lipoprotein-induced endothelial cells injury. *BMC Complement. Altern. Med.* **2018**, *18*, 1–11, doi:10.1186/s12906-018-2215-8.

12. Cao, X.P.; Chen, Y.F.; Zhang, J.L.; You, M.M.; Wang, K.; Hu, F.L. Mechanisms underlying the wound healing potential of propolis based on its in vitro antioxidant activity. *Phytomedicine* **2017**, *34*, 76–84, doi:10.1016/j.phymed.2017.06.001.

13. Botta, L.; Brunori, F.; Tulimieri, A.; Piccinino, D.; Meschini, R.; Saladino, R. Laccase-Mediated Enhancement of the Antioxidant Activity of Propolis and Poplar Bud Exudates. *ACS Omega* **2017**, *2*, 2515–2523, doi:10.1021/acsomega.7b00294.

14. Li, A.; Xuan, H.; Sun, A.; Liu, R.; Cui, J. Preparative separation of polyphenols from water-soluble fraction of Chinese propolis using macroporous absorptive resin coupled with preparative high performance liquid chromatography. *J. Chromatogr. B* **2016**, *1012–1013*, 42–49, doi:10.1016/j.jchromb.2015.12.038.

15. Zhang, J.; Shen, X.; Wang, K.; Cao, X.; Zhang, C.; Zheng, H.; Hu, F. Antioxidant activities and molecular mechanisms of the ethanol extracts of Baccharis propolis and Eucalyptus propolis in RAW64.7 cells. *Pharm. Biol.* **2016**, *54*, 2220–2235, doi:10.3109/13880209.2016.1151444.

16. Xuan, H.; Wang, Y.; Li, A.; Fu, C.; Wang, Y.; Peng, W. Bioactive Components of Chinese Propolis Water Extract on Antitumor Activity and Quality Control. *Evidence-based Complement. Altern. Med.* **2016**, *2016*, doi:10.1155/2016/9641965.

17. Tian, H.; Sun, H.; Zhang, J.; Zhang, X.; Zhao, L.; Guo, S.; Li, Y.; Jiao, P.; Wang, H.; Qin, S.; et al. Ethanol extract of propolis protects macrophages from oxidized low density lipoprotein-induced apoptosis by inhibiting CD36 expression and endoplasmic reticulum stress-C / EBP homologous protein pathway. *BMC Complement. Altern. Med.* **2015**, 1–12, doi:10.1186/s12906-015-0759-4.

18. Cui-ping, Z.; Shuai, H.; Wen-ting, W.; Shun, P.; Xiao-ge, S.; Ya-jing, L.; Fu-Liang, H. Development of High-Performance Liquid Chromatographic for Quality and Authenticity Control of Chinese Propolis. *J. Food Sci.* **2014**, *79*, 1–8, doi:10.1111/1750-3841.12510.

19. Wang, K.; Ping, S.; Huang, S.; Hu, L.; Xuan, H.; Zhang, C.; Hu, F. Molecular mechanisms underlying the in vitro anti-inflammatory effects of a flavonoid-rich ethanol extract from chinese propolis (poplar type). *Evidence-based Complement. Altern. Med.* **2013**, *2013*, doi:10.1155/2013/127672.

20. Nie, P.; Xia, Z.; Sun, D.W.; He, Y. Application of visible and near infrared spectroscopy for rapid analysis of chrysin and galangin in chinese propolis. *Sensors (Switzerland)* **2013**, *13*, 10539–104590, doi:10.3390/s130810539.

21. Shi, H.; Yang, H.; Zhang, X.; Yu, L. *Identification and quantification of phytochemical composition and anti-inflammatory and radical scavenging properties of methanolic extracts of Chinese propolis*; 2012; Vol. 60; ISBN 8621342057.

22. Yang, H.; Dong, Y.; Du, H.; Shi, H.; Peng, Y.; Li, X. Antioxidant compounds from propolis collected in Anhui, China. *Molecules* **2011**, *16*, 3444–3455,

doi:10.3390/molecules16043444.

23. Yang, C.; Luo, L.; Zhang, H.; Yang, X.; Lv, Y.; Song, H. Common aroma-active components of propolis from 23 regions of China. *J. Sci. Food Agric.* **2010**, *90*, 1268–1282, doi:10.1002/jsfa.3969.

24. Sha, N.; Guan, S.H.; Lu, Z.Q.; Chen, G.T.; Huang, H.L.; Xie, F.B.; Yue, Q.X.; Liu, X.; Guo, D.A. Cytotoxic constituents of Chinese propolis. *J. Nat. Prod.* **2009**, *72*, 799–801, doi:10.1021/np900118z.

25. Sha, N.; Huang, H.L.; Zhang, J.-Q.; Chen, G.T.; Tao, S.-J.; Yang, M.; Li, X.-N.; Li, P.; Guo, D. Simultaneous Quantification of Eight Major Bioactive Phenolic Compounds in Chinese Propolis by. *Nat. Prod. Commun.* **2009**, *4*, 813–818.

26. Usia, T.; Banskota, A.H.; Tezuka, Y.; Midorikawa, K.; Matsushige, K.; Kadota, S. Constituents of Chinese propolis and their antiproliferative activities. *J. Nat. Prod.* **2002**, *65*, 673–676, doi:10.1021/np010486c.

27. El-Guendouz, S.; Lyoussi, B.; Miguel, M.G. Insight on Propolis from Mediterranean Countries: Chemical Composition, Biological Activities and Application Fields. *Chem. Biodivers.* **2019**, *16*, doi:10.1002/cbdv.201900094.

28. Kasote, D.M.; Pawar, M. V.; Bhatia, R.S.; Nandre, V.S.; Gundu, S.S.; Jagtap, S.D.; Kulkarni, M. V. HPLC, NMR based chemical profiling and biological characterisation of Indian propolis. *Fitoterapia* **2017**, *122*, 52–60, doi:10.1016/j.fitote.2017.08.011.

29. Sadhana, N.; Lohidasan, S.; Mahadik, K.R. Marker-based standardization and investigation of nutraceutical potential of Indian propolis. *J. Integr. Med.* **2017**, *15*, 483–494, doi:10.1016/S2095-4964(17)60360-1.

30. Choudhari, M.K.; Punekar, S.A.; Ranade, R. V; Paknikar, K.M. Antimicrobial activity of stingless bee (Trigona sp .) propolis used in the folk medicine of Western Maharashtra , India. *J. Ethnopharmacol.* **2012**, *141*, 363–367, doi:10.1016/j.jep.2012.02.047.

31. Naik, D.G.; Vaidya, H.S.; Namjoshi, T.P. Essential Oil of Indian Propolis : Chemical Composition and Repellency against the Honeybee Apis florea. **2013**, *10*, 649–657.

32. Miyata, R.; Sahlan, M.; Ishikawa, Y.; Hashimoto, H.; Honda, S.; Kumazawa, S. Propolis Components from Stingless Bees Collected on South Sulawesi,

Indonesia, and Their Xanthine Oxidase Inhibitory Activity. *J. Nat. Prod.* **2019**, *82*, 205–2010, doi:10.1021/acs.jnatprod.8b00541.

33. Trusheva, B.; Todorov, I.; Ninova, M.; Najdenski, H.; Daneshmand, A.; Bankova, V. Antibacterial mono- and sesquiterpene esters of benzoic acids from Iranian propolis. **2010**, 1–4.

34. Alizadeh, A.M.; Afrouzan, H.; Dinparast-Djadid-Navid; Sawaya, A.C.H.F.; Azizian, S.; Hemmati, H.R.; Mohagheghi, M.A.; Erfani, S. Chemoprotection of MNNG-initiated Gastric Cancer in Rats Using Iranian Propolis. **2015**, *18*, 18–23.

35. Asgharpur, F.; Moghadamnia, A.A.; Kazemi, S.; Nouri, H.R.; Motallebnejad, M. Applying GC-MS analysis to identify chemical composition of Iranian propolis prepared with different solvent and evaluation of its biological activity. **2020**, *11*, 191–198, doi:10.22088/cjim.11.2.191.

36. Bazmandegan, G.; Boroushaki, M.T.; Shamsizadeh, A.; Ayoobi, F.; Hakimizadeh, E.; Allahtavakoli, M. Brown propolis attenuates cerebral ischemia-induced oxidative damage via affecting antioxidant enzyme system in mice. *Biomed. Pharmacother.* **2017**, *85*, 503–510, doi:10.1016/j.biopha.2016.11.057.

37. Mohammadzadeh, S.; Shariatpanahi, M.; Hamedi, M.; Ahmadkhaniha, R.; Samadi, N.; Ostad, S.N. Chemical composition, oral toxicity and antimicrobial activity of Iranian propolis. *Food Chem.* **2007**, *103*, 1097–1103, doi:10.1016/j.foodchem.2006.10.006.

38. Tukmechi, A.; Ownagh, A.; Mohebbat, A. In vitro antibacterial activities of ethanol extract of Iranian propolis (EEIP) against fish pathogenic bacteria (Aeromonas hydrophila, Yersinia ruckeri & Streptococcus iniae). *Brazilian J. Microbiol.* 2010, *41*, 1086–1092.

39. Sulaiman, G.M.; Sammarrae, K.W.A.; Ad'hiah, A.H.; Zucchetti, M.; Frapolli, R.; Bello, E.; Erba, E.; D'Incalci, M.; Bagnati, R. Chemical characterization of iraqi propolis samples and assessing their antioxidant potentials. *Food Chem. Toxicol.* **2011**, *49*, 2415–2421, doi:10.1016/j.fct.2011.06.060.

40. Kumazawa, S.; Ueda, R.; Hamasaka, T.; Fukumoto, S.; Fujimoto, T.; Nakayama, T. Antioxidant prenylated flavonoids from propolis collected in Okinawa, Japan. *J. Agric. Food Chem.* **2007**, *55*, 7722–7725, doi:10.1021/jf071187h.

41. Kumazawa, S.; Nakamura, J.; Murase, M.; Miyagawa, M.; Ahn, M.R.; Fukumoto, S. Plant origin of Okinawan propolis: Honeybee behavior observation and phytochemical analysis. *Naturwissenschaften* **2008**, *95*, 781–786, doi:10.1007/s00114-008-0383-y.

42. Shaheen, S.A.; Zarga, M.H.A.; Nazer, I.K.; Darwish, R.M.; Al-Jaber, H.I. Chemical constituents of Jordanian propolis. *Nat. Prod. Res.* **2011**, *25*, 1312–1318,

doi:10.1080/14786419.2010.509060.

43. Abutaha, N. Apoptotic Potential and Chemical Composition of Jordanian Propolis Extract against Different Cancer Cell Lines. *J. Microbiol. Biotechnol.* **2019**, *30*, 893–902, doi:10.4014/jmb.1905.05027.

44. Noureddine, H.; Hage-Sleiman, R.; Wehbi, B.; Fayyad-Kazan, A.H.; Hayar, S.; Traboulssi, M.; Alyamani, O.A.; Faour, W.H.; ElMakhour, Y. Chemical characterization and cytotoxic activity evaluation of Lebanese propolis. *Biomed. Pharmacother.* **2017**, *95*, 298–307, doi:10.1016/j.biopha.2017.08.067.

45. Ong, T.H.; Chitra, E.; Ramamurthy, S.; Siddalingam, R.P.; Yuen, K.H.; Ambu, S.P.; Davamani, F. Chitosan-propolis nanoparticle formulation demonstrates anti-bacterial activity against Enterococcus faecalis biofilms. *PLoS One* **2017**, *12*, 1–22, doi:10.1371/journal.pone.0174888.

46. Usman, U.Z.; Bakar, A.B.A.; Mohamed, M. Phytochemical composition and activity against hyperglycaemia of Malaysian propolis in diabetic rats. *Biomed. Res.* **2016**, *27*, 46–51.

47. Li, F.; Awale, S.; Zhang, H.; Tezuka, Y.; Esumi, H.; Kadota, S. Chemical constituents of propolis from Myanmar and their preferential cytotoxicity against a human pancreatic cancer cell line. *J. Nat. Prod.* **2009**, *72*, 1283–1287, doi:10.1021/np9002433.

48. Li, F.; Awale, S.; Tezuka, Y.; Kadota, S. Cytotoxic constituents of propolis from Myanmar and their structure-activity relationship. *Biol. Pharm. Bull.* **2009**, *32*, 2075–2078, doi:10.1248/bpb.32.2075.

49. Awale, S.; Shrestha, S.P.; Tezuka, Y.; Ueda, J.Y.; Matsushige, K.; Kadota, S. Neoflavonoids and related constituents from nepalese propolis and their nitric oxide production inhibitory activity. *J. Nat. Prod.* **2005**, *68*, 858–864, doi:10.1021/np050009k.

50. Funakoshi-Tago, M.; Okamoto, K.; Izumi, R.; Tago, K.; Yanagisawa, K.; Narukawa, Y.; Kiuchi, F.; Kasahara, T.; Tamura, H. Anti-inflammatory activity of flavonoids in Nepalese propolis is attributed to inhibition of the IL-33 signaling pathway. *Int. Immunopharmacol.* **2015**, *25*, 189–198, doi:10.1016/j.intimp.2015.01.012.

51. Funakoshi-Tago, M.; Ohsawa, K.; Ishikawa, T.; Nakamura, F.; Ueda, F.; Narukawa, Y.; Kiuchi, F.; Tamura, H.; Tago, K.; Kasahara, T. Inhibitory effects of flavonoids extracted from Nepalese propolis on the LPS signaling pathway. *Int. Immunopharmacol.* **2016**, *40*, 550–560, doi:10.1016/j.intimp.2016.10.008.

52. Okińczyc, P.; Paluch, E.; Franiczek, R.; Widelski, J.; Wojtanowski, K.K.; Mroczek, T.; Krzyżanowska, B.; Skalicka-Woźniak, K.; Sroka, Z. Antimicrobial activity of Apis mellifera L. and Trigona sp. propolis from Nepal and its phytochemical analysis. *Biomed. Pharmacother.* **2020**, *129*, doi:10.1016/j.biopha.2020.110435.

53. Shrestha, S.P.; Narukawa, Y.; Takeda, T. Chemical constituents of Nepalese propolis (II). *Chem. Pharm. Bull.* **2007**, *55*, 926–929, doi:10.1248/cpb.55.926.

54. Popova, M.; Dimitrova, R.; Al-Lawati, H.T.; Tsvetkova, I.; Najdenski, H.; Bankova, V. Omani propolis: Chemical profiling, antibacterial activity and new propolis plant sources. *Chem. Cent. J.* **2013**, *7*, 1–8, doi:10.1186/1752-153X-7-158.

55. Desamero, M.J.; Kakuta, S.; Tang, Y.; Chambers, J.K.; Uchida, K.; Estacio, M.A.; Cervancia, C.; Kominami, Y.; Ushio, H.; Nakayama, J.; et al. Tumor-suppressing potential of stingless bee propolis in in vitro and in vivo models of differentiated-type gastric adenocarcinoma. *Sci. Rep.* **2019**, *9*, 1–13, doi:10.1038/s41598-019-55465-4.

56. Ragasa, C.Y.; Galian, R.A.F.; Ebajo, V.D.; Aguda, R.M.; Cervancia, C.R.; Shen, C.C. Propolins and glyasperin a from stingless bee nests. *Rev. Bras. Farmacogn.* **2015**, *25*, 177–179, doi:10.1016/j.bjp.2015.03.006.

57. Almutairi, S.; Edrada-Ebel, R.; Fearnley, J.; Igoli, J.O.; Alotaibi, W.; Clements, C.J.; Gray, A.I.; Watson, D.G. Isolation of diterpenes and flavonoids from a new type of propolis from Saudi Arabia. *Phytochem. Lett.* **2014**, *10*, 160–163, doi:10.1016/j.phytol.2014.08.022.

58. Alqarni, A.S.; Rushdi, A.I.; Owayss, A.A.; Raweh, H.S.; El-Mubarak, A.H.; Simoneit, B.R.T. Organic tracers from asphalt in propolis produced by urban honey bees, Apis mellifera Linn. *PLoS One* **2015**, *10*, 1–18, doi:10.1371/journal.pone.0128311.

59. Elnakady, Y.A.; Rushdi, A.I.; Franke, R.; Abutaha, N.; Ebaid, H.; Baabbad, M.; Omar, M.O.M.; Al Ghamdi, A.A. Characteristics, chemical compositions and biological activities of propolis from Al-Bahah, Saudi Arabia. *Sci. Rep.* **2017**, *7*, 1–13, doi:10.1038/srep41453.

60. Jerz, G.; Elnakady, Y.A.; Braun, A.; Jäckel, K.; Sasse, F.; Al Ghamdi, A.A.; Omar, M.O.M.; Winterhalter, P. Preparative mass-spectrometry profiling of bioactive metabolites in Saudi-Arabian propolis fractionated by high-speed countercurrent chromatography and off-line atmospheric pressure chemical ionization mass-spectrometry injection. *J. Chromatogr. A* **2014**, *1347*, 17–29, doi:10.1016/j.chroma.2014.04.068.

61. Said, S.A.; Khan, S.A.; Ahmad, I.; Ali, H.S. Chemical composition of Egyptian and UAE propolis. *Pak. J. Pharm. Sci.* **2006**, *19*, 58–61.

62. Seyhan, M.F.; Yılmaz, E.; Timirci-Kahraman, Ö.; Saygılı, N.; Kısakesen, H.İ.; Gazioğlu, S.; Gören, A.C.; Eronat, A.P.; Begüm Ceviz, A.; Öztürk, T.; et al. Different propolis samples, phenolic content, and breast cancer cell lines: Variable cytotoxicity ranging from ineffective to potent. *IUBMB Life* **2019**, *71*, 619–631, doi:10.1002/iub.1996.

63. Aru, B.; Güzelmeric, E.; Akgül, A.; Demirel, G.Y.; Kırmızıbekmez, H. Antiproliferative Activity of Chemically Characterized Propolis from Turkey and Its Mechanisms of Action. *Chem. Biodivers.* **2019**, *16*, doi:10.1002/cbdv.201900189.

64. Çelemli, Ö.G.; Hatjina, F.; Charistos, L.; Schiesser, A.; Özkirim, A. More insight into the chemical composition of Greek propolis; differences and similarities with Turkish propolis. *Zeitschrift fur Naturforsch. - Sect. C J. Biosci.* **2013**, *68 C*, 429–438, doi:10.1515/znc-2013-11-1201.

65. Tugba Degirmencioglu, H.; Guzelmeric, E.; Yuksel, P.I.; Kırmızıbekmez, H.; Deniz, I.; Yesilada, E. A New Type of Anatolian Propolis: Evaluation of Its Chemical Composition, Activity Profile and Botanical Origin. *Chem. Biodivers.* **2019**, *16*, doi:10.1002/cbdv.201900492.

66. Duran, N.; Muz, M.; Culha, G.; Duran, G.; Ozer, B. GC-MS analysis and antileishmanial activities of two Turkish propolis types. *Parasitol. Res.* **2011**, *108*, 95–105, doi:10.1007/s00436-010-2039-z.

67. Gülçin, I.; Bursal, E.; Şehitoĝlu, M.H.; Bilsel, M.; Gören, A.C. Polyphenol contents and antioxidant activity of lyophilized aqueous extract of propolis from Erzurum, Turkey. *Food Chem. Toxicol.* **2010**, *48*, 2227–2238, doi:10.1016/j.fct.2010.05.053.

68. Guzelmeric, E.; Ristivojević, P.; Trifković, J.; Dastan, T.; Yilmaz, O.; Cengiz, O.; Yesilada, E. Authentication of Turkish propolis through HPTLC fingerprints combined with multivariate analysis and palynological data and their comparative antioxidant activity. *LWT - Food Sci. Technol.* **2018**, *87*, 23–32, doi:10.1016/j.lwt.2017.08.060.

69. Kargar, N.; Matin, G.; Matin, A.A.; Buyukisik, H.B. Biomonitoring, status and source risk assessment of polycyclic aromatic hydrocarbons (PAHs) using honeybees, pine tree leaves, and propolis. *Chemosphere* **2017**, *186*, 140–150, doi:10.1016/j.chemosphere.2017.07.127.

70. Kartal, M.; Kaya, S.; Kurucu, S. GC-MS analysis of propolis samples from two different regions of Turkey. *Zeitschrift fur Naturforsch. - Sect. C J. Biosci.* **2002**, *57*, 905–909, doi:10.1515/znc-2002-9-1025.

71. Keskin, N.; Hazir, S.; Baser, H. can; Kürkçüoglub, M. Antibacterial activity of Turkish propolis and its qualitative and quantitative chemical composition.

Phytomedicine **2005**, *12*, 221–228, doi:10.1016/j.phymed.2003.09.007.

72. Ahn, M.R.; Kumazawa, S.; Usui, Y.; Nakamura, J.; Matsuka, M.; Zhu, F.; Nakayama, T. Antioxidant activity and constituents of propolis collected in various areas of China. *Food Chem.* **2007**, *101*, 1383–1392, doi:10.1016/j.foodchem.2006.03.045.

73. Lee, I.K.; Han, M.S.; Kim, D.W.; Yun, B.S. Phenylpropanoid acid esters from Korean propolis and their antioxidant activities. *Bioorganic Med. Chem. Lett.* **2014**, *24*, 3503–3505, doi:10.1016/j.bmcl.2014.05.065.

74. Shimomura, K.; Sugiyama, Y.; Nakamura, J.; Ahn, M.R.; Kumazawa, S. Component analysis of propolis collected on Jeju Island, Korea. *Phytochemistry* **2013**, *93*, 222–229, doi:10.1016/j.phytochem.2012.02.018.

75. Ho Jin, Un, Tae-Wook Chung, Sung-Koo Kang, Seok-Jong Suh, J.-K.K.; Kang-Hyun Chung, Yeun-Hwa Gu, Ikukatsu Suzuki, C.-H.K. Caffeic acid phenyl ester in propolis is a strong inhibitor of matrix metalloproteinase-9 and invasion inhibitor: Isolation and identification. *Clin. Chim. Acta* **2005**, *362*, 57–64.

76. SHIMOMURA, Kohsuke, Saori INUI, Yasumasa SUGIYAMA, M.K.; Jun NAKAMURA, Su-Jin CHOI, M.-R.A.& S.K. Identification of the Plant Origin of Propolis from Jeju Island, Korea, by Observation of Honeybee Behavior and Phytochemical Analysis. *Biosci. Biotechnol. Biochem* **2012**, *76*, 2135–2138, doi:https://doi.org/10.1271/bbb.120580.

77. Popova, M.; Chen, C.N.; Chen, P.Y.; Huang, C.Y.; Bankova, V. A validated spectrophotometric method for quantification of prenylated flavanones in pacific propolis from Taiwan. *Phytochem. Anal.* **2010**, *21*, 186–191, doi:10.1002/pca.1176.

78. Chen, Y.W.; Ye, S.R.; Ting, C.; Yu, Y.H. Antibacterial activity of propolins from Taiwanese green propolis. *J. Food Drug Anal.* **2018**, *26*, 761–768, doi:10.1016/j.jfda.2017.10.002.

79. Athikomkulchai, S.; Awale, S.; Ruangrungsi, N.; Ruchirawat, S.; Kadota, S. Chemical constituents of Thai propolis. *Fitoterapia* **2013**, *88*, 96–100, doi:10.1016/j.fitote.2013.04.008.

80. Boonsai, P.; Phuwapraisirisan, P.; Chanchao, C. Antibacterial activity of a cardanol from thai apis mellifera propolis. *Int. J. Med. Sci.* **2014**, *11*, 327–336, doi:10.7150/ijms.7373.

81. Sanpa, S.; Popova, M.; Bankova, V.; Tunkasiri, T.; Eitssayeam, S.; Chantawannakul, P. Antibacterial compounds from propolis of Tetragonula laeviceps and Tetrigona melanoleuca (Hymenoptera: Apidae) from Thailand. *PLoS One* **2015**, *10*, 1–8, doi:10.1371/journal.pone.0126886.

82. Teerasripreecha, D.; Phuwapraisirisan, P.; Puthong, S.; Kimura, K.; Okuyama, M.; Mori, H.; Kimura, A.; Chanchao, C. In vitro antiproliferative/cytotoxic activity on cancer cell lines of a cardanol and a cardol enriched from Thai Apis mellifera propolis. *BMC Complement. Altern. Med.* **2012**, *12*, 1–17, doi:10.1186/1472-6882-12-27.

83. Chewchinda, S.; Vongsak, B. Development and validation of a high-performance thin layer chromatography method for the simultaneous quantitation of α- and γ-mangostins in Thai stingless bee propolis. *Rev. Bras. Farmacogn.* **2019**, *29*, 333–338, doi:10.1016/j.bjp.2018.12.004.

84. Kumazawa, S.; Hamasaka, T.; Nakayama, T. Antioxidant activity of propolis of various geographic origins. *Food Chem.* **2004**, *84*, 329–339, doi:10.1016/S0308-8146(03)00216-4.

85. Georgieva, K.; Popova, M.; Dimitrova, L.; Trusheva, B.; Thanh, L.N.; Lan Phuong, D.T.; Lien, N.T.P.; Najdenski, H.; Bankova, V. Phytochemical analysis of Vietnamese propolis produced by the stingless bee Lisotrigona cacciae. *PLoS One* **2019**, *14*, 1–13, doi:10.1371/journal.pone.0216074.

86. Popova, M.; Trusheva, B.; Bankova, V. Propolis of stingless bees: A phytochemist's guide through the jungle of tropical biodiversity. *Phytomedicine* **2019**, 153098, doi:10.1016/j.phymed.2019.153098.

87. Nguyen, H.X.; Nguyen, M.T.T.; Nguyen, N.T.; Awale, S. Chemical Constituents of Propolis from Vietnamese Trigona minor and Their Antiausterity Activity against the PANC-1 Human Pancreatic Cancer Cell Line. *J. Nat. Prod.* **2017**, *80*, 2345–2352, doi:10.1021/acs.jnatprod.7b00375.

88. Al-Ghamdi, A.A.; Bayaqoob, N.I.M.; Rushdi, A.I.; Alattal, Y.; Simoneit, B.R.T.; El-Mubarak, A.H.; Al-Mutlaq, K.F. Chemical compositions and characteristics of organic compounds in propolis from Yemen. *Saudi J. Biol. Sci.* **2017**, *24*, 1094–1103, doi:10.1016/j.sjbs.2016.12.012.

89. Ozdal, T.; Ceylan, F.D.; Eroglu, N.; Kaplan, M.; Olgun, E.O.; Capanoglu, E. Investigation of antioxidant capacity, bioaccessibility and LC-MS/MS phenolic profile of Turkish propolis. *Food Res. Int.* **2019**, *122*, 528–536, doi:10.1016/j.foodres.2019.05.028.

90. Popova, M.; Silici, S.; Kaftanoglu, O.; Bankova, V. Antibacterial activity of Turkish propolis and its qualitative and quantitative chemical composition. *Phytomedicine* **2005**, *12*, 221–228, doi:10.1016/j.phymed.2003.09.007.

91. Silici, S.; Kutluca, S. Chemical composition and antibacterial activity of propolis collected by three different races of honeybees in the same region. *J. Ethnopharmacol.* **2005**, *99*, 69–73, doi:10.1016/j.jep.2005.01.046.

92. Silici, S.; Ünlü, M.; Vardar-Ünlü, G. Antibacterial activity and phytochemical evidence for the plant origin of Turkish propolis from different regions. *World J. Microbiol. Biotechnol.* **2007**, *23*, 1797–1803, doi:10.1007/s11274-007-9430-7.

93. Sorkun, Kadriye, B.S. and B.S. Determination of Chemical Composition of Turkish Propolis. *Verlag der Zeitschrift für Naturforsch.* **2001**, 48–50.

94. Velikova, M.; Bankova, V.; Sorkun, K.; Houcine, S.; Tsvetkova, I.; Kujumgiev, A. Propolis from the Mediterranean region: Chemical composition and antimicrobial activity. *Zeitschrift fur Naturforsch. - Sect. C J. Biosci.* **2000**, *55*, 790–793, doi:10.1515/znc-2000-9-1019.

95. Koru, O.; Toksoy, F.; Acikel, C.H.; Tunca, Y.M.; Baysallar, M.; Uskudar Guclu, A.; Akca, E.; Ozkok Tuylu, A.; Sorkun, K.; Tanyuksel, M.; et al. In vitro antimicrobial activity of propolis samples from different geographical origins against certain oral pathogens. *Anaerobe* **2007**, *13*, 140–145, doi:10.1016/j.anaerobe.2007.02.001.

96. Hanif, A.; Nurdiansyah, R.; Hawa, P.; Wahyu, D.; Eko, K.; Dwi, C.; Taufiqu, N.; Novan, N.; Noviyanto, A.; Mardliyati, E. In silico investigation of potential inhibitors to main protease and spike protein of SARS-CoV-2 in propolis. *Biochem. Biophys. Reports* **2021**, *26*, 100969, doi:10.1016/j.bbrep.2021.100969.

97. Nakamura, R.; Nakamura, R.; Watanabe, K.; Oka, K.; Ohta, S. International Immunopharmacology Effects of propolis from different areas on mast cell degranulation and identi fi cation of the effective components in propolis. *Int. Immunopharmacol.* **2010**, *10*, 1107–1112, doi:10.1016/j.intimp.2010.06.013.

98. Sun, C.; Wu, Z.; Wang, Z.; Zhang, H. Effect of ethanol/water solvents on phenolic profiles and antioxidant properties of Beijing propolis extracts. *Evidence-based Complement. Altern. Med.* **2015**, *2015*, doi:10.1155/2015/595393.
